# Annotation and comparative analysis of the glycoside hydrolase genes in *Brachypodium distachyon*

**DOI:** 10.1186/1471-2164-11-600

**Published:** 2010-10-25

**Authors:** Ludmila Tyler, Jennifer N Bragg, Jiajie Wu, Xiaohan Yang, Gerald A Tuskan, John P Vogel

**Affiliations:** 1 USDA-ARS Western Regional Research Center, Albany, CA 94710, USA; 2 Department of Plant and Microbial Biology, University of California, Berkeley, CA 94720, USA; 3 Department of Plant Sciences, University of California, Davis, CA 95616, USA; 4 Biosciences Division and BioEnergy Science Center, Oak Ridge National Laboratory, Oak Ridge, TN 37831, USA

## Abstract

**Background:**

Glycoside hydrolases cleave the bond between a carbohydrate and another carbohydrate, a protein, lipid or other moiety. Genes encoding glycoside hydrolases are found in a wide range of organisms, from archea to animals, and are relatively abundant in plant genomes. In plants, these enzymes are involved in diverse processes, including starch metabolism, defense, and cell-wall remodeling. Glycoside hydrolase genes have been previously cataloged for *Oryza sativa *(rice), the model dicotyledonous plant *Arabidopsis thaliana*, and the fast-growing tree *Populus trichocarpa *(poplar). To improve our understanding of glycoside hydrolases in plants generally and in grasses specifically, we annotated the glycoside hydrolase genes in the grasses *Brachypodium distachyon *(an emerging monocotyledonous model) and *Sorghum bicolor *(sorghum). We then compared the glycoside hydrolases across species, at the levels of the whole genome and individual glycoside hydrolase families.

**Results:**

We identified 356 glycoside hydrolase genes in *Brachypodium *and 404 in sorghum. The corresponding proteins fell into the same 34 families that are represented in rice, *Arabidopsis*, and poplar, helping to define a glycoside hydrolase family profile which may be common to flowering plants. For several glycoside hydrolase familes (GH5, GH13, GH18, GH19, GH28, and GH51), we present a detailed literature review together with an examination of the family structures. This analysis of individual families revealed both similarities and distinctions between monocots and eudicots, as well as between species. Shared evolutionary histories appear to be modified by lineage-specific expansions or deletions. Within GH families, the *Brachypodium *and sorghum proteins generally cluster with those from other monocots.

**Conclusions:**

This work provides the foundation for further comparative and functional analyses of plant glycoside hydrolases. Defining the *Brachypodium *glycoside hydrolases sets the stage for *Brachypodium *to be a grass model for investigations of these enzymes and their diverse roles *in planta*. Insights gained from *Brachypodium *will inform translational research studies, with applications for the improvement of cereal crops and bioenergy grasses.

## Background

Glycoside hydrolases (GHs) are enzymes that hydrolyze the bond between a carbohydrate and another compound, such as a second carbohydrate, a protein, or a lipid [1]. The Carbohydrate-Active Enzymes (CAZy) database categorizes GHs into at least 108 different families, defined by sequence similarity [1,2]. GH genes are present in a wide range of organisms from archaea and bacteria to animals and plants. Not surprisingly, given plants' photosynthetic capacity and their carbohydrate-rich cell walls, plants contain a relative abundance of genes for carbohydrate-active enzymes, including GHs [3]. Although experimental characterizations of plant GHs are limited, these enzymes have been assigned a broad array of functions. They are implicated in the defense against pathogens through attacks on the carbohydrate components of microbial cell walls, the mobilization of energy reserves through the degradation of starch, and hormone signaling through the cleavage of inactivating glycosyl groups from hormone conjugates, among many other processes [4]. Some plant GHs are thought to function in the synthesis, remodeling, and degradation of plant cell walls [4-6]. Developmental events involving cell-wall loosening or degradation include cell expansion, seed germination, lateral root emergence, stomatal formation, xylem differentiation, pollen tube growth, and fruit ripening [7-10]. The recalcitrance, or resistance to degradation, of cell walls is a major obstacle to the efficient conversion of plant feedstocks into biofuels [11]. Therefore, in addition to their roles *in planta*, GHs capable of modifying cell walls are also of interest for biofuel applications [5].

Here, we present the annotation and analysis of GH genes in the model grass species *Brachypodium distachyon *(referred to hereafter as *Brachypodium*). *Brachypodium *is a small annual in the grass subfamily the Pooideae. *Brachypodium*'s short stature; simple growth requirements; amenability to genetic transformation; and compact, sequenced genome make *Brachypodium *a suitable research model for its less-tractable grass relatives [12-17]. Members of the grass family, the Poaceae, provide the majority of the world's food and feed. Key crops are included in the subfamilies Ehrhartoideae (rice), Panicoideae (maize and sorghum), and Pooideae (wheat, oat, and barley) [18]. Increasingly, grasses are also being exploited for fuel: Species such as *Miscanthus *(*Miscanthus × giganteus*) and switchgrass (*Panicum virgatum*) are being investigated as dedicated energy crops for the production of biofuels [19,20], and crop residues from maize, rice, and wheat may also be utilized as biomass feedstocks [21]. With carbohydrates as major components of grains, fodder, and cellulosic biofuel feedstocks, a better understanding of carbohydrate-active enzymes in the grasses is needed.

Genome-wide analyses of GH genes have been previously published for one grass, *Oryza sativa *(rice) [22], and two dicotyledonous plants, the model species *Arabidopsis thaliana *(hereafter *Arabidopsis*) [23] and the fast-growing tree *Populus trichocarpa *(poplar) [24]. Comparisons between *Arabidopsis *and rice or *Arabidopsis *and poplar have been used to draw conclusions about the evolutionary history of GHs or differences in the GH profiles of large plant groups. For example, *Arabidopsis *and rice GH28 family members were compared to estimate the number of GH28 genes in the common ancestor of these divergent species [25]. Also, differences in the number of GHs between *Arabidopsis *and rice or poplar have been hypothesized to reflect differences in the GH profiles of dicotyledonous and monocotyledonous plants (*Arabidopsis *versus rice) [22] or herbaceous and woody plants (*Arabidopsis *versus poplar) [24]. The sequencing of additional plant genomes allows such comparisons to be extended to more species, increasing the robustness of the analyses by reinforcing the conclusions or by identifying over-generalizations from pairwise comparisons. To improve our understanding of plant GHs generally and grass GHs specifically, we have annotated both the *Brachypodium *and *Sorghum bicolor *(sorghum) GHs and compared them to the GHs from rice, *Arabidopsis*, and poplar. When significant differences between the grasses and eudicots were identified, we broadened the analysis to include GHs from other species (maize, wheat, soybean, *Medicago*, castor bean, tomato, *etc*.) with significant, but variable, available sequence resources. This large-scale analysis will help guide research into this important group of enzymes.

## Methods

### Identification of *Brachypodium *GH genes

Rice and *Arabidopsis *GH protein sequences were retrieved from the CAZy database [1,2] and used as queries in BLASTp searches [26] of the version 1.0 predicted proteome of *Brachypodium*, including splice variants [27]. Additional files 1 and 2 list rice and *Arabidopsis *GH sequences, respectively. The *E*-value cut-off was set to 10^-10^. For GH families with no known rice or *Arabidopsis *representatives, the *Brachypodium *predicted proteome was searched using another, usually microbial, GH sequence selected from the CAZy list. Using the Pfam database [28,29], each candidate *Brachypodium *GH was analyzed for the presence of a predicted GH domain. The Pfam domain predictions are listed in additional file 3. To further confirm GH family assignments, *Brachypodium *GH sequences were used as queries in tBLASTn searches against GenBank entries, March to May, 2009 [30]. Each gene model was then individually examined using expressed sequence tag (EST), Illumina transcriptome, and splice-junction data, as well as predicted alternative transcripts, for *Brachypodium *(available at http://www.brachypodium.org) [27,31]; relevant gene models from *Arabidopsis *and rice (accessible at http://mips.helmholtz-muenchen.de/proj/plant/jsf/brachypodium/index.jsp) [27,32]; and Pfam domain predictions, to decide whether and how a *Brachypodium *gene model could be improved through manual modifications. The few *Brachypodium *GH models which were modified are indicated with an "m" beside the gene name in additional file 3. The modifications and modified sequences are listed in additional file 4.

To search for GH genes possibly omitted from the version 1.0 annotation, *Brachypodium *EST sequences, including Sanger and 454 sequencing reads as well as the TAU models built from Illumina short reads [27], were mapped onto the unmasked *Brachypodium *genome sequence [27] using BLAT [33] with a minimum identity of 92%. Only the "best match" position was selected as the genomic location for each query EST sequence. Gene models were then predicted using Augustus [34], with the genomic locations of the *Brachypodium *ESTs as extrinsic evidence. The protein sequences of predicted *Brachypodium *gene models were compared with *Arabidopsis *(version 8 [35]) and rice genome annotations (version 6 [36]) using BLASTp [37]. For motif analysis, protein sequences were scanned for domains using blastprodom, coils, gene3d, hmmpanther, hmmpir, hmmpfam, hmmsmart, hmmtigr, fprintscan, patternscan, profilescan, and superfamily implemented in InterPro [38-41]. The resulting candidate GHs were individually evaluated, as described above, for possible improvements to the gene models. The GH genes identified in this analysis of the unmasked genome are noted in additional file 4. Protein sequences for all the *Brachypodium *GHs are listed in additional file 5.

### Identification of sorghum GH genes

Sorghum GHs were identified in the same way, except that rice, *Arabidopsis*, and *Brachypodium *GH protein sequences were used as queries in BLASTp searches of the Sbi1_4 version (Sorbi1_GeneModels_Sbi1_4_aa.fasta.gz) [42] of the predicted proteome of sorghum [43]. In contrast to the analysis performed for *Brachypodium*, sorghum GH gene models were not systematically evaluated for potential errors, nor did we search for GH genes not contained in the annotation. Additional files 6 and 7 list the sorghum GHs and their protein sequences.

### Construction of phylogenetic trees

Full-length GH protein sequences from *Arabidopsis*, rice, *Brachypodium*, sorghum, and poplar were used as the basis for constructing phylogenetic trees. *Arabidopsis *and rice sequences were accessed through the CAZy database [1,2]. *Brachypodium *and sorghum GH sequences were identified in this study, and poplar GH sequences were identified via BLASTp searches of the version 1.1 *Populus *proteome (proteins.Poptr1_1.JamboreeModels) [44,45], using *Arabidopsis *and rice GH proteins as queries. GH sequences from additional species (maize, wheat, soybean, castor bean, grape, tomato, *Medicago*, strawberry, *etc*.) were later incorporated into selected trees. These additional sequences were either downloaded from the CAZy database, identified by querying GenBank [30,46] from November of 2009 through May of 2010 with known GH sequences, or retrieved from the research literature. Sequences were aligned by ClustalW [47] using default parameters (a Gonnet protein weight matrix and gap-opening penalties of 10 and gap-extending penalties of 0.1 and 0.2 for pair-wise and multiple alignments, respectively) implemented in the MEGA4 program [48]. The ClustalW alignments were manually examined and found to be highly accurate. Thus, no manual adjustments were made except for the elimination of entire proteins that appeared to be truncated or otherwise incorrectly annotated. Phylogenetic analyses were performed in MEGA4, using the Neighbor-Joining method [49] and 1,000 bootstrap replicates [50] for each analysis. Pairwise deletion was employed to address alignment gaps and missing data.

## Results and discussion

### Identification of *Brachypodium *and sorghum GHs

To identify candidate *Brachypodium *GHs, BLASTp searches [26] of the version 1.0 *Brachypodium *predicted proteome [27] were performed using rice and *Arabidopsis *GH sequences as queries. The resulting candidates were compared against the Pfam protein families database [28] to detect protein domains. *Brachypodium *proteins without predicted GH domains were removed from consideration, with the following exceptions: one *Brachypodium *GH33 and two GH95 members were considered to be GHs because the Pfam database does not contain a specific entry for either a GH33 or a GH95 domain. In these cases, we relied on the *Brachypodium *proteins' high sequence similarity to rice and *Arabidopsis *family members. Two of the five *Brachypodium *GH27 family members lacked a significant match to a Pfam GH domain but were nevertheless considered to be GHs because they are highly similar to rice and *Arabidopsis *GH27 family members which also lack a predicted Pfam GH domain. After modification of the gene model, one additional gene (Bradi1g27870) was determined to encode a GH16 protein. These analyses identified 340 *Brachypodium *GH genes. Since the version 1.0 annotation, based on a repeat-masked *Brachypodium *genomic sequence, was missing genes in other families, such as the F-box family [27], we also searched for *Brachypodium *GH genes in the unmasked genome, using an annotation pipeline based on transcriptome expression evidence as well as a protein domain search. This secondary search yielded an additional 16 *Brachypodium *GHs. In total, 356 *Brachypodium *genes in 34 GH families were identified; the full list is given in additional file 3. Protein sequences for the *Brachypodium *GHs are listed in additional file 5.

The gene models for all of the *Brachypodium *GHs were examined for possible improvements. Of the 356 GH gene models, 14 (3.9%) were modified based on criteria such as EST data and gene models from other species. Additional file 4 details the modifications and additions made to version 1.0 of the *Brachypodium *genome annotation. Nearly 80% of the *Brachypodium *GH genes were supported by EST and/or Illumina transcriptome data (additional file 3) [27]. The limited changes compared to the version 1.0 annotation and the large proportion of genes with expression support testify to the high quality of the initial genome annotation.

Approximately 84% of identified *Brachypodium *GHs had good matches (*E*-value ≤ 10^-100^) in both rice and *Arabidopsis *(additional file 3). Another 14% of *Brachypodium *GHs matched both rice and *Arabidopsis *GH sequences with an *E*-value ≤ 10^-10^. Only 7 *Brachypodium *GHs - in the GH5, GH16, and GH18 families - were good matches to rice GHs but lacked clear *Arabidopsis *orthologs (additional file 3). As discussed below, these GH5 and GH18 sequences represent major clades which are missing in *Arabidopsis*. No *Brachypodium *GHs were found outside the families represented in rice and *Arabidopsis*, despite our queries using GHs from other organisms.

**Figure 1:**
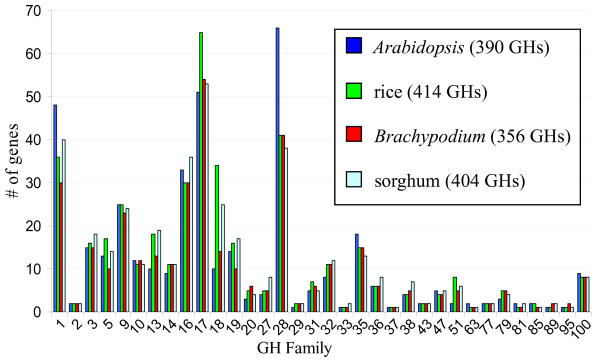
**Number of GH family members in *Arabidopsis*, rice, *Brachypodium*, and sorghum**. GH families are indicated on the x-axis, with the number of family members in each species indicated on the y-axis. Numbers in parentheses in the figure key indicate the total number of GH genes in each species.

A similar approach was used to identify sorghum GHs: rice, *Arabidopsis*, and *Brachypodium *GH protein sequences were used as queries in BLASTp searches of the sorghum predicted proteome [43], and the resulting candidates were analyzed for the presence of Pfam-predicted GH domains [28]. Sorghum was found to have 404 GHs in the same 34 families that are represented in *Arabidopsis*, rice, and *Brachypodium *(Figure 1 and additional file 8). Additional files 6 and 7 include the full list of sorghum GHs and their corresponding protein sequences.

### Comparisons to *Arabidopsis *and rice GHs

To compare GH profiles across plant species, we retrieved the numbers of GH family members in both *Arabidopsis *and rice from the CAZy database [1,2]. The number of gene family members was updated as follows: one putative GH28 gene (At1g23470) was removed from the *Arabidopsis *list, because At1g23470 is annotated as a pseudogene in the most recent, TAIR9, release of the *Arabidopsis *genome [35]. The number of rice genes in four families was reduced because several genes were listed multiple times. The GH5 gene Os10g0370800 was listed three times, the GH16 gene Os08g0240500 twice, and the GH17 gene Os01g0947000 twice. Two CAZy entries for the rice GH32 family [GenBank: AAD10239.1 and AAK72492.2] correspond to genes already included in the list; we therefore considered these entries to be duplicates. Also, two fragmentary rice sequences [GenBank: BAA01617.1 and BAG87724.1] were removed from the GH13 and GH36 families, respectively.

Overall, *Brachypodium *and sorghum have similar numbers of GHs as rice and *Arabidopsis*. The 356 GHs in *Brachypodium *represent 1.4% of the 25,532 predicted protein-coding genes [27], while the 404 GHs in sorghum correspond to 1.5% of the 27,640 protein-coding genes [43]. This is in comparison to 390 GHs in *Arabidopsis *(1.4% of the 27,379 TAIR9 protein-coding genes) [35] and 414 GHs in rice (1.4% of the 30,192 RAP2 protein-coding genes) [51,52].

Despite the global similarity in the total number of GHs, the number of members in individual GH families varies widely. For *Arabidopsis*, rice, *Brachypodium*, and sorghum, the number of GHs in each family is shown graphically in Figure 1 and numerically in additional file 8. Some families - *e.g. *GH2, 9, 10, 37, 43, 77, and 100 - contain similar numbers of members in each of the four species. In other families - *e.g. *GH1, 5, 13, 18, 19, 28, and 51 - the number of members differs by up to four-fold (Figure 1 and additional file 8). Previous studies have detected some of these differences. For example, the GH28 family of polygalacturonases is larger in *Arabidopsis *(66 genes) than rice (41 genes); conversely, the GH18 family of chitinases is much larger in rice (34 genes) than *Arabidopsis *(10 genes) [22] (Figure 1 and additional file 8). However, with such pairwise comparisons of species, it has been difficult to evaluate whether differences represent variation between major groups, such as dicots and monocots, or merely between the two species examined. For many GH families, the *Brachypodium *and sorghum data are largely consistent with those for rice. The identification of 41 GH28 members in *Brachypodium *and 38 in sorghum supports the idea that grasses contain fewer polygalacturonases than eudicots do. In other cases, such as the GH18 family, the trend breaks down: *Arabidopsis*, rice, *Brachypodium*, and sorghum have 10, 34, 14, and 25 GH18 genes, respectively (Figure 1 and additional file 8). This observation highlights the danger of making large taxonomic generalizations based on pairwise comparisons.

### Poplar GHs

The unusually high number of GH genes reported for poplar (600 genes) [24] complicates global comparisons with other species; some of the poplar gene models may actually be annotation artifacts arising from the heterozygous nature of the large, complex, and duplicated poplar genome. Whereas multiple rounds of computational and manual improvements have resulted in high-quality *Arabidopsis *and rice gene models [35,36,52], the sorghum and poplar models are "first drafts" derived from computational predictions, gene homology, and expression data [43,45]. Future refinements of the sorghum and poplar gene models may therefore alter the number of GHs, as well as the corresponding protein sequences, in these species. In fact, while cataloging carbohydrate-active enzymes in poplar, Geisler-Lee *et al. *found that some models were fragmentary and should be merged into larger genes [24].

For detailed analyses of specific GH families, poplar sequences were retrieved via BLASTp searches of the version 1.1 *Populus *proteome with *Arabidopsis *and rice GH proteins as queries. The searches yielded non-identical, although largely overlapping, sets of poplar GH candidates. For example, poplar reportedly has 22 GH5 family members, identified via BLAST searches using all entries in the CAZy database [24]. Our searches of the poplar proteome using the *Arabidopsis *GH sequences identified 17 poplar GH5s; searches using the rice GHs identified an additional 5 poplar GH5 proteins, for a total of 22. These results are consistent with the finding of Tuskan *et al*. that almost 12% of predicted poplar genes did not have clear orthologs in *Arabidopsis *[45].

Poplar was not known to have any members in the GH33 and GH85 families [24]. Although the GH33 and GH85 families are small, with one to two members each in *Arabidopsis*, rice, *Brachypodium*, and sorghum (Figure 1 and additional file 8), it was surprising that poplar would completely lack representatives of these families. Interestingly, our searches identified one poplar GH33 gene (Poptr825914) and three poplar GH85 genes (Poptr226914, Poptr226918, and Poptr419935). (See the additional file 9 for the full gene names and sequences.) The poplar GH33 was an especially good match - with an *E-*value of 10^-147 ^- to the rice GH33, Os07g0516000. The Pfam database does not list a specific GH33 domain, and, correspondingly, Poptr825914 and the GH33 family members in *Arabidopsis*, rice, *Brachypodium*, and sorghum do not have any significant matches to Pfam GH domains. However, analyzing the Poptr825914 protein with the InterProScan feature of the InterPro database [40] identified a sialidase domain, which is characteristic of the GH33 family [2]. The three poplar GH85 proteins, Poptr226914, Poptr226918, and Poptr419935, were all retrieved as matches to *Arabidopsis *and rice GH85 sequences, and all contain a characteristic, Pfam-predicted Glycosyl hydrolase family 85 domain.

The identification of GH33 and GH85 members in poplar means that poplar has the same GH families which are present in *Arabidopsis*, rice, *Brachypodium*, and sorghum. The presence of these families in five, diverse flowering plant species, combined with the apparent absence of plant sequences from other families, suggests that these 34 GH families are common to angiosperms.

### Phylogenetic analyses

To further elucidate the relationships between plant GHs, we selected several families for phylogenetic analyses. Full-length protein sequences from five species - *Arabidopsis*, rice, *Brachypodium*, sorghum, and poplar - served as the basis for building phylogenetic trees. Making the trees with full-length sequences allowed all the information contained in the protein sequences to contribute to the phylogenetic placement of the genes. To be sure that this overall evolutionary history agreed with the GH domain alone, we also constructed trees based only on the GH domains (not shown). For the GH18, GH19, GH5, GH28, and GH13 families, the domain-only trees had the same structure as the trees based on the full-length sequences. For the GH51 family, the bootstrap values in the domain-only tree were too low to be informative for distinguishing the highly-similar sequences. Thus, in this case, the sequence outside the GH domain was crucial for teasing out the relationship between the proteins. To enrich the investigation, sequences from other organisms were included for some of the GH families. These evolutionary analyses, performed with the MEGA4 program [48], emphasize comparisons between eudicots and grasses, especially the model plants *Arabidopsis *and *Brachypodium*.

### The GH18 and 19 families

Chitin, a long-chain polymer of beta-1,4-N-acetyl-D-glucosamine (GlcNAc) linkages, is the second-most-abundant carbohydrate in nature after cellulose. It forms the major component of fungal cell walls and is also found in the exoskeletons of insects and shells of mollusks [53]. Chitinases are enzymes that break down chitin by hydrolyzing this polysaccharide into simple sugars, and chitinolytic enzymes have been identified in viruses, bacteria, fungi, protozoan parasites, insects, animals, and plants [1,30]. Chitin is not synthesized in plants. However, expression of several plant chitinases is induced by pathogen challenge, and these proteins make up five of the seventeen families of plant pathogenesis-related (PR) proteins: PR2, PR3, and PR4 are GH19 family members, and PR8 and PR11 are GH18 family members [4,54]. This implicates chitinases as key plant-defense proteins. Numerous studies have demonstrated that chitinases have both antifungal and antibacterial activities [55-61]. Environmental stresses such as drought, salinity, frost, wounding and osmotic pressure also can induce chitinase expression in plants. Other studies suggest that chitinases likely play a role in growth, development, and the generation or degradation of signaling molecules [62-66]. Nod factors produced by nitrogen-fixing soil bacteria include chitin oligomers of four or five N-acetyl glucosamine residues that can be cleaved and inactivated by specific plant chitinases, revealing a role for these proteins in symbiosis. It is not surprising, then, that plant genomes contain a large number of chitinase genes, the majority of which are classified in the GH18 and GH19 families. Together these two families comprise 24 genes in *Brachypodium*, 42 in sorghum, 50 in rice, 24 in *Arabidopsis *and 56 in poplar (additional files 8 and 9).

### The GH18 family

Despite shared chitinolytic activity, the GH18 and GH19 families do not share sequence similarity. The two families are clearly distinguished by their sequences and three-dimensional structures, indicating they are derived from different ancestral genes. The GH18 and GH19 plant chitinases are further divided into seven classes (I-VII) based on amino acid sequence and the presence or absence of auxiliary domains flanking a catalytic domain [67,68]. The GH18 family includes the class III and class V chitinases that are more closely related to fungal enzymes involved in morphogenesis (class III) and bacterial exochitinases (class V) than they are to GH19 proteins [67]. The GH18 domain is an eight-stranded β/α barrel with a pronounced active-site cleft at the C-terminal end of the β-barrel and a conserved DXXDXDXE motif [69,70]. GH18 class III chitinases can have dual lysozyme and chitinase functions, and these dual-function proteins tend to be better-targeted at murein in bacterial cell walls than the other classes of chitinases [4]. The GH18 family also includes a number of "inactivated" chitinases which represent evolutionary adaptations that recruit the ancient and stable GH18 scaffold to novel functions. These include GH18 xylanase inhibitor proteins (XIPs) that lack chitinolytic activity but have adapted a new defense mechanism targeting the pathogen-produced GH10 and GH11 xylanases that degrade arabinoxylans in plant cell walls [69,71]. Nodulins, involved in interactions with symbiotic bacteria, as well as narbonins and concanavalin B, seed proteins lacking conserved catalytic residues, also group within the GH18 family [67,69].

**Figure 2:**
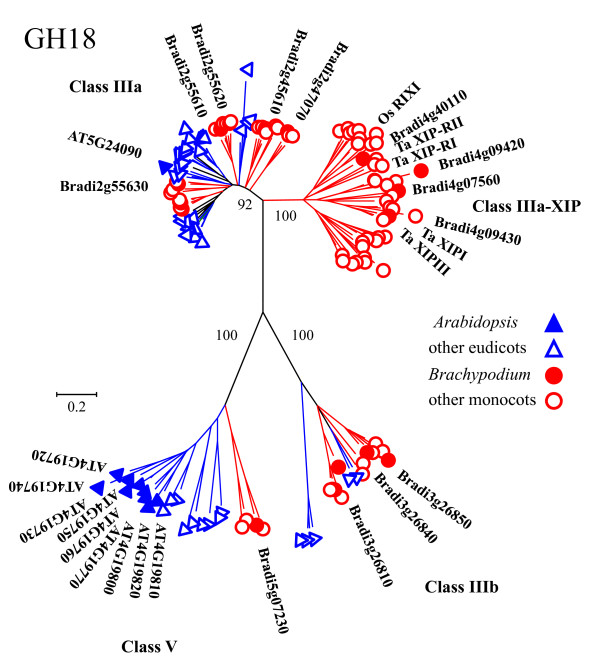
**The GH18 chitinase family**. The tree includes GH18 proteins from 23 species: *Arabidopsis *(AT), rice, *Brachypodium *(Bradi), sorghum, and poplar, as well as sequences from 13 other eudicots (sugar beet, longleaf ironwood, jelly fig, strawberry, soybean, white lupine, *Medicago*, a legume, winged bean, ginseng, adzuki bean, cowpea, and grape) and 5 other monocots (bamboo, bread wheat, wild tulip, durum wheat, and maize). The tree was constructed using the Neighbor-Joining method and 1,000 bootstrap replicates. The percent of bootstrap replicates supporting each major branch is indicated. Distances are proportional to the number of amino-acid substitutions per site. Sequences from eudicots are indicated in blue (*Arabidopsis *with filled triangles, other eudicots with open triangles); sequences from monocots are indicated in red (*Brachypodium *with filled circles, other monocots with open circles). For clarity, gene names or accession numbers are shown only for *Arabidopsis *and *Brachypodium*. Chitinase class designations are listed by each clade and specific XIPs are labeled. For complete branch labels and bootstrap values, see additional file 10.

A phylogenetic analysis of the GH18 proteins from 23 plant species is presented in Figure 2 and additional file 10. The Neighbor-Joining tree was generated using 1000 bootstrap replicates and includes 8 monocot (*Brachypodium*, rice, sorghum, bamboo, tulip, bread wheat, durum wheat, and maize) and 15 eudicot (*Arabidopsis*, poplar, sugar beet, longleaf ironwood, jelly fig, strawberry, soy bean, white lupine, *Medicago*, a legume, winged bean, ginseng, adzuki bean, cowpea, and grape) species. For the scientific names of species in this tree, see additional file 11. Sequences within this family resolve into four distinct, well-supported clades. The class V chitinases group into a single clade that shares little sequence similarity with the class III proteins. Monocot and eudicot sequences group separately within the class V clade. *Brachypodium *and rice each have one representative in this group and sorghum has two; interestingly, class V chitinases have not been reported in maize [55]. Compared to these monocots, the eudicots *Arabidopsis *and poplar have greater representation in the class V clade. This is explained by local duplications resulting in expansion of class V chitinases in these species.

GH18 class III chitinases are divided between the remaining three clades of the tree. Initially, we aligned sequences from *Brachypodium*, rice, sorghum, *Arabidopsis*, and poplar, and in the analysis of these data, two clades comprised only grass sequences (data not shown). These sequences were used to query the NCBI (National Center for Biotechnology Information) non-redundant protein sequence database [30] in order to expand the number of species represented. The retrieved proteins from both monocot and eudicot species were used in combination with the original set to construct a new tree. The majority of the retrieved eudicot sequences aligned with the sequences in the class IIIa group that contains the only *Arabidopsis *class III protein. In this clade, the monocot and dicot sequences are mixed, rather than forming two distinct groupings as seen for the class V proteins (Figure 2).

The class IIIb clade in Figure 2 was proposed by Suzukawa *et al. *(2003) [72] in studies of a tulip-bulb chitinase and was also used by Shoresh and Harmon (2008) [55] to describe a group of maize GH18 proteins. In our original tree, this group contained only monocot sequences; yet it is closely related to the GH18 narbonin and nodulin-like proteins. Narbonin is a globulin protein from the eudicot *Vicia narbonensis *that lacks conserved chitinase catalytic residues and enzymatic activity. In legumes such as fava bean and soybean, nodulins are induced in response to signals generated by symbiotic bacteria. These are eudicot species, and therefore, it was apparent that we needed to expand our dataset to get a better understanding of the proteins that cluster with the narbonin and nodulin proteins and the neighboring class IIIb group. A BLASTp search retrieved a variety of eudicot sequences that group within the IIIb clade. Soresh and Harmon had compared maize sequences only to rice, tulip, and *Arabidopsis *and concluded that class IIIb is monocot-specific, because the only dicot considered, *Arabidopsis*, did not have a representative in the IIIb group. Our original tree included poplar, which also lacks a class IIIb protein. However, the presence of several eudicot class IIIb sequences in our expanded tree (Figure 2 and additional file 10) illustrates the need for caution when drawing conclusions based on data from a few species. The narbonin and nodulin nucleotide sequences do not encode predicted signal peptides. In their analysis of one tulip, two rice, and three maize class IIIb proteins, Soresh and Harmon reported that these sequences also lack signal peptides. We used Signal P and Sig-Pred software to evaluate signal peptide predictions for the *Brachypodium *and sorghum GH18 proteins and found that the Bradi3g26840, Bradi3g26850, and Sb5g006880 sequences in the class IIIb clade lack predicted signal peptides. However, the class IIIb proteins Bradi3g26810 and Sb01g21920, as well as all of the other *Brachypodium *and sorghum GH18 proteins, do contain predicted signal peptides.

**Figure 3:**
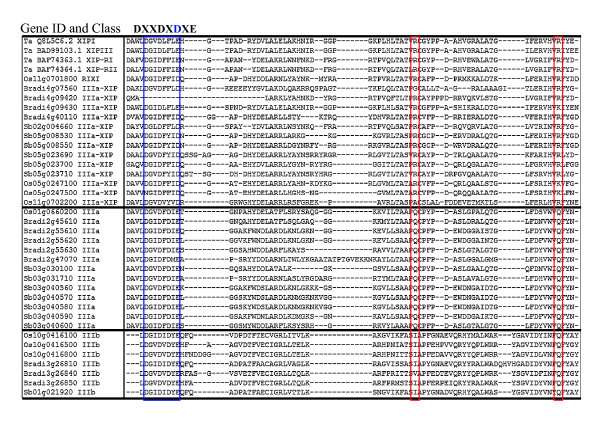
**Alignment of GH18 class III chitinase sequences**. Forty sequences were selected from the one hundred fourteen class IIIa, IIIa-XIP, and IIIb sequences used to construct the GH18 family phylogenetic tree. The selected proteins include the experimentally verified wheat (Ta) and rice (Os) proteins. All of the *Brachypodium *(Bradi) class III proteins were included in the alignment along with sorghum (Sb) and rice proteins selected to represent branches of the class III clades. The sequences were aligned using the Clustal W method in MegAlign 5.05 (DNAStar software package). The region presented includes the DXXDXDXE motif (within the blue box) required for chitinolytic activity and two residues (within the red boxes) that in most of the IIIa-XIP sequences contain amino acids (R or K) capable of forming salt bridges with the E in the DXXDXDXE motif. The class IIIa-XIP protein Bradi4g09429 contains a large N-proximal deletion, and the first amino acid shown is at position 33 in the sequence. The position of the first amino acid shown ranges from positions 139 to 159 for the other class IIIa-XIP proteins, from positions 139 to150 for class IIIa, and from positions 125 to 149 for class IIIb.

The remainder of the class III chitinases form a distinct clade that remains monocot-specific even after the addition of BLASTp-retrieved monocot and eudicot sequences to the phylogenetic analysis. These proteins have been grouped with the class IIIa chitinases; however, they can be clearly distinguished from the class IIIa chitinases by sequence and functional characteristics. This distinct clade represents a group of xylanase inhibitor proteins (XIPs) that have lost chitinolytic activity and have gained the ability to inhibit the GH10 and GH11 xylanases used by pathogens to attack plant cell walls [69,71]. XIPs for which reports have been published, wheat XIP-I and XIP-III [73], wheat XIP-RI and XIP-RII [56], and rice RIXI [69], are labeled in Figure 2. In our analysis, the clade containing these sequences is referred to as class IIIa-XIP. The class IIIa-XIP chitinases have a modified version of the DXXDXDXE motif that is required for catalytic activity. In XIPs, the third D is usually mutated to an aromatic residue (F or Y). In addition, these proteins contain two mutations introducing R residues at positions C-terminal to the DXXDXDXE motif that can form salt bridges with the conserved E residue. This interaction blocks the chitin-binding site, preventing chitinase activity [56,69]. The sorghum and maize proteins within the clusters containing RIXI, XIP-RI, XIP-RII, and XIPI all contain the characteristic XIP mutations. All of the *Brachypodium *sequences within the class IIIa-XIP clade also have these changes, suggesting that they may function as XIPs, whereas the *Brachypodium *class IIIa and IIIb sequences do not contain these mutations (Figure 3). Bradi4g09420 contains the two R mutations, but in place of the active site mutation, it has a large deletion through the chitin-binding domain, entirely eliminating the DXXDXDXE motif in this protein. This deletion is unique among the proteins included in the class IIIa-XIP clade. The cluster of the class IIIa-XIP clade that does not contain a *Brachypodium *representative also appears to consist of XIP proteins. Most of the rice, sorghum, and maize sequences within this group have the three described XIP substitutions; however, a few sequences have variations. The Os05g0247100, Os05g0247500, Os05g0248200 sequences have an additional N substitution in place of the first D in the catalytic motif. Os11g002200 has an A instead of the first R, and Os05g0247100, Os05g0247500, Os05g0248200 each have a K instead of the second R (Figure 3 and data not shown). The considerable number of sequences for each species represented in this clade is mainly explained by a large number of local duplications of class IIIa-XIP genes. The evolution of XIP proteins from class III chitinases appears to represent a successful functional adaptation specific to monocots, and, in this analysis, particularly for grasses.

The functional adaptations within the GH18 family highlight the challenges of assigning protein functions based solely on sequence similarities. Numerous studies have been performed to identify the targets of class III chitinases. Chitinases have different substrate specificities, activities, reaction mechanisms, and expression patterns. Activity and substrate specificity are diverse even among identical classes of enzymes. For example, two of the rice class IIIb proteins, Os10g0416500 and Os10g0416800, have highly similar sequences. Yet Os10g0416500 is expressed in response to pathogen challenge and has substantial antifungal activity, whereas Os10g0416800 is expressed in response to environmental stresses [59]. Through the comparison of increasing numbers of chitinase sequences, however, new groups emerge, such as the class IIIa-XIP proteins, and functional predictions based on sequence patterns begin to become possible.

### The GH19 family

The GH19 family is found primarily in plants, but members have been identified in a number of bacteria and in nematodes [1]. Analyses of GH19 proteins reveal structural similarities with lysozymes, despite a lack of significant sequence similarity, and suggest that these two enzyme groups arose from a common ancestor originating before the divergence of prokaryotes and eukaryotes [30]. The chitinase classes represented in the GH19 family (I, II, IV, VI, and VII) are distinguished by characteristic small deletions in the sequence and by the presence of auxiliary domains flanking the main catalytic domain, including a cysteine-rich chitin-binding domain (CRD or CBD), a proline- and glycine-rich hinge region, and a carboxy-terminal extension (CTE) [55,67,74].

**Figure 4:**
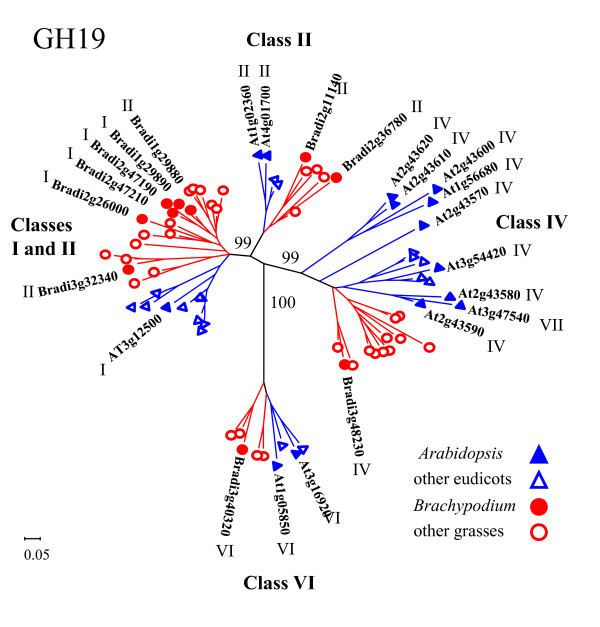
**The GH19 chitinase family**. The tree includes GH19 proteins from five species: *Arabidopsis *(AT), rice, *Brachypodium *(Bradi), sorghum, and poplar. Tree construction is as in Figure 2. Sequences from eudicots are indicated in blue (*Arabidopsis *with filled triangles, poplar with open triangles); sequences from grasses are indicated in red (*Brachypodium *with filled circles, rice and sorghum with open circles). For clarity, gene names or accession numbers are shown only for *Arabidopsis *and *Brachypodium*. Chitinase class designations are listed by each clade and are indicated next each of the *Arabidopsis *and *Brachypodium *proteins. For complete branch labels and bootstrap values, see additional file 12.

A phylogenetic analysis of the GH19 proteins from five plant species is presented in Figure 4 and additional file 12. The Neighbor-Joining tree includes three grass (*Brachypodium*, rice, and sorghum) and two eudicot (*Arabidopsis *and poplar) species. The sequences within this family are resolved into four distinct, well-supported clades (Figure 4). We used the designated chitinase classes for the *Arabidopsis *GH19 proteins [35,75] and the sequence alignment generated for this tree to assign chitinase classes to *Brachypodium *proteins with similar arrangements of auxiliary domains and deletion patterns. Class designations for the *Brachypodium *and *Arabidopsis *sequences are indicated by Roman numerals within Figure 4. Three of the clades contain sequences primarily from a single chitinase class, II, IV, or VI, whereas one clade contains sequences mixed between classes I and II. Sequences for classes I and II, although similar, are distinguished by the absence of the CRD and the CTE and a small deletion in the catalytic domain in the class II proteins. Placement in previous phylogenetic analyses also suggests that class II proteins have polyphyletic origins, arising multiple times within class I lineages [67]. Two of the *Brachypodium *sequences in this clade have notable differences from the classic representatives of their assigned classes. Bradi2g26000 has a large CRD containing 16 cysteine residues, twice the usual number, and may represent two tandem CRDs. Bradi1g29880 looks like a class II protein but lacks the signal peptide usually found in this class. One class VII protein, At3g47540, resides within the class IV clade. In addition to the two deletions in the catalytic domain that are characteristic of the class IV proteins, this protein also lacks CRD and CTE regions. Previously, classes II and IV were reported to be found mainly in dicots [67]. However, the three grass species included in this analysis, *Brachypodium*, rice, and sorghum, each have representatives in the clades containing these class II and IV chitinases, suggesting that monocot sequences have been heretofore under-represented in these classes. There are few examples of class VI and VII proteins in the literature, and we identified only one class VI and no class VII proteins amongst the *Brachypodium *chitinases. Class VI chitinases have an arrangement of auxiliary domains similar to class I proteins but contain a CRD with fewer cysteine residues than the class I domain, as well as variations in the hinge and CTE regions. One of the *Arabidopsis *proteins designated class VI in this analysis, At1g05850, earlier was reported to be a class VII protein [75]. However, At1g05850 lacks the catalytic-domain deletions characteristic of the class IV and VII proteins. As more sequences from additional species are added to such analyses, a clearer picture of the profiles of these families emerges.

Each clade of the GH19 family contains at least one representative from all five species analyzed; however, the grass and eudicot sequences group as separate clusters within the clades (Figure 4 and additional file 12). The difference in the number of genes between species is primarily due to localized, tandem duplications of sequences within the two larger clades. Three good examples are the five *Arabidopsis *genes (At2g43580, At2g43590, At2g43600, At2g43610, and At2g43620); six sorghum genes (Sb06g021210, Sb06g021220, Sb06g021230, Sb06g021240, Sb06g021250, and Sb06g021260); and four poplar genes (Poptr249950, Poptr547380, Poptr249966, and Poptr826290) present in the class IV clade. Additionally, the clade with mixed class I and II sequences shows expansions in two *Brachypodium *regions (Bradi1g29880 and Bradi1g29890, as well as Bradi2g47190 and Bradi2g47210); one poplar region (Poptr557015, Poptr557013, Poptr649160, Poptr72170, Poptr 72160, Poptr649163, and Poptr200449); and two rice regions (Os05g0399300, Os05g0399400, and Os05g0399700, as well as Os06g726100 and Os06g726200). One possible explanation for this observation is proposed by Bishop *et al. *[76] as a result of their analyses of the PR proteins represented by the GH19 class I chitinases [67]. These researchers observed that the GH19 proteins disproportionately accumulate adaptive mutations in the active-site cleft. This unusual pattern of mutation is not shared by chitinases of the GH18 family, suggesting that adaptive functional modifications rapidly emerge as a result of direct pathogen defense against plant chitinolytic activity. This plant-pathogen coevolution of GH19 genes could be facilitated by the observed gene duplications: Mutations in the additional gene copies could confer adaptive advantages in the face of attacks by a variety of pathogens.

### The GH5 family

The GH5 family, previously named cellulase family A, includes plant-cell-wall-modifying enzymes such as cellulases, mannanases, and *β*-glucosidases [1,2,4,5]. The enzymatic activities of a few plant GH5 members have been characterized. One of these is HvMAN1, a mannanase (EC 3.2.1.78) from barley (*Hordeum vulgare*) [77]. Purified from 10-day-old seedlings, HvMAN1 exhibited relatively high rates of hydrolysis on moderately substituted galactomannan and unsubstituted glucomannan substrates [77]. Another mannanase, LeMAN4a, expressed in ripenning tomato (*Solanum lycopersicon*, syn. *Lycopersicon esculentum*), was also cloned, its endo- *β*-D-mannanase activity confirmed in an *in vitro *assay, and its structure solved [78,79]. RNA-mediated suppression of *LeMAN4a *expression slightly increased the firmness of ripening tomato fruits, suggesting that LeMAN4a plays a supporting role in fruit softening [80]. The rice *GH5BG *gene (Os10g0370500) encodes a GH5 family *β*-glucosidase that is expressed in the shoots of seedlings and leaf sheaths of adult plants [81]. Salt stress, submergence, and the stress hormones methyl jasmonate and abscisic acid induced the expression of *GH5BG*, hinting at a possible connection between GH5BG-mediated cell-wall remodeling and responses to environmental conditions [81].

**Figure 5:**
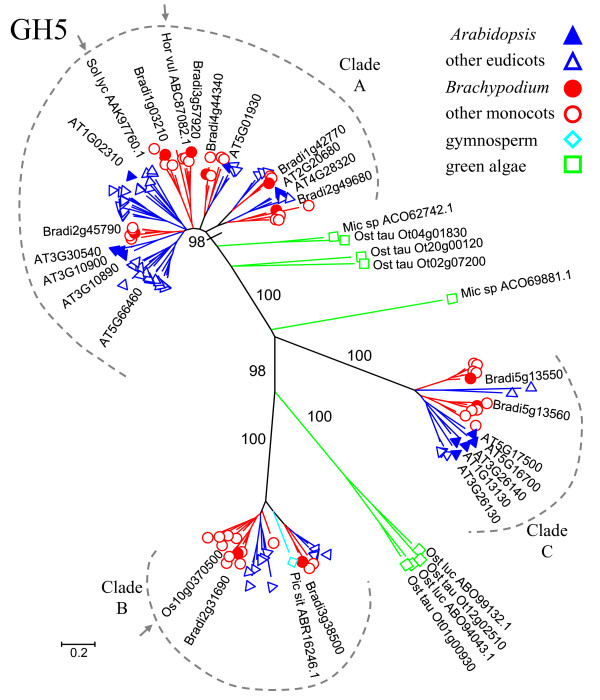
**The GH5 family of putative cell-wall-modifying enzymes**. The tree includes GH5 proteins from 28 species: *Arabidopsis *(AT), rice, *Brachypodium *(Bradi), sorghum, and poplar, as well as 14 additional eudicot species, five additional monocot species, a gymnosperm, and three species of green algae. Tree construction and color-coding (blue for eudicots and red for monocots) are as in Figure 2. A light blue diamond indicates the single gymnosperm sequence; green squares indicate green algal sequences. The three major plant clades, A, B, and C, are marked with dotted, gray lines. Small gray arrows indicate HvMAN1 (Hor vul ABC87082.1), LeMAN4a (Sol lyc AAK97760.1), and GH5BG (Os10g0370500). For clarity, gene names or accession numbers are shown only for *Arabidopsis*, *Brachypodium*, the gymnosperm, and algae. Mic sp: *Micromonas *sp. RCC299; Ost luc: *Ostreococcus lucimarinus*; Ost tau: *Ostreococcus tauri*; Pic sit: *Picea sitchensis*. For complete branch labels and bootstrap values, see additional file 13.

To obtain a more complete picture of the plant GH5 family, GH5 sequences from other plants (barley, maize, wheat, tomato, coffee, soybean, apple, peach, tomato, grape, *etc*.) and from green algae (*Micromonas *and *Ostreococcus *species) were retrieved from the CAZy database or, in the case of HvMAN1, from the research literature. (For a full list of species, see additional file 11.) These protein sequences were combined with the *Arabidopsis*, rice, *Brachypodium*, sorghum, and poplar GH5 sequences to build an evolutionary tree. The plant GH5 proteins formed three major clades, A, B, and C, with high bootstrap support (Figure 5 and additional file 13). The mannanases HvMAN1 and LeMAN4a are in clade A, while the *β*-glucosidase GH5BG is in clade B (Figure 5). Initially, clade B appeared to contain only monocot sequences and one sequence from a gymnosperm, sitka spruce (*Picea sitchensis*). However, tBLASTn searches of GenBank using the two *Brachypodium *clade B representatives yielded additional members from dicotyledonous species: poplar, castor bean, tomato, and grape (Figure 5). For complete information on species included in the tree and branch labels, see additional files 11 and 13. Strikingly, the *Arabidopsis *GH5 proteins fall within Clades A and C, but not B (Figure 5). Performing tBLASTn searches of the *Arabidopsis *transcriptome (TAIR9) [35] using the *Brachypodium *and eudicot Clade B members as queries yielded no Clade B matches, confirming the lack of an *Arabidopsis *Clade B representative. Sequences from green algae (*Ostreococcus lucimarinus*, *Ostreococcus tauri*, and *Micromonas*) cluster with clades A and B with 100% and 98% bootstrap support, respectively (Figure 5). This finding suggests that both the A and B clades arose early in evolution. The absence of *Arabidopsis *from clade B, a group spanning diverse plant species, underscores the point that, although *Arabidopsis *is a powerful tool, it is not sufficient for studying all aspects of plants. Multi-species comparisons are needed to form a comprehensive picture.

### The GH28 family

The GH28 family includes polygalacturonases, which act on pectin [1,2,4]. Pectin consists of carbohydrate polymers (*e.g. *homogalacturonans and rhamnogalacturonans) rich in galacturonic acid [82]. Pectin is a major component of the middle lamella connecting adjacent plant cells to each other [9,82]. In eudicots such as *Arabidopsis*, pectin is also abundant in primary cell walls, where it forms a matrix surrounding the network of cellulose and hemicellulose [82,83]. GH28 polygalacturonases have been implicated in the reduction of cell-to-cell adhesion and the remodeling of cell walls, contributing to developmental processes such as pollen development, organ abscission, and fruit ripening [4,9,84]. For instance, loss-of-function mutations in the *Arabidopsis *GH28 family member At3g07970 - also known as *QUARTET2 *(*QRT2*) - result in the production of tetrad pollen, caused by the failure of the four microspores to separate following meiosis [84,85]. *QRT2 *and the related genes *ARABIDOPSIS DEHISCENCE ZONE POLYGALACTURONASE1 *and *2 *(*ADPG1 *and *ADPG2*) are all involved in anther dehiscence, a cell-separation process which allows for pollen release [84]. *QRT2 *and *ADPG2 *(At2g41850) both contribute to floral-organ shedding [84,86]; *ADPG1 *(At3g57510) and *ADPG2 *together promote the dehiscence of seed pods [84]. *In vitro *biochemical assays have confirmed the polygalacturonase activity of ADPG1, ADPG2, and the protein encoded by another GH28 family member, At1g48100 [84]. Twenty years ago, in tomato, it was shown that suppressing the expression of an endogenous polygalacturonase with antisense RNA inhibited the degradation of pectin during fruit ripening [87,88]. Since then, the importance of polygalacturonase activity in the ripening of many other fruits has also been demonstrated [9].

Both tandem and whole-genome duplications have contributed to the presence of a relatively large number of GH28 genes in *Arabidopsis *compared to rice [22,25]. The type I cell walls of dicots contain much higher levels of pectin than the type II walls of grasses [89], and it has been proposed that the increased number of GH28s in *Arabidopsis *reflects a greater need for pectin-active enzymes [22]. Consistent with this hypothesis, maize (*Zea mays*) was recently reported to have 16 fewer GH28 genes than *Arabidopsis *[90], even though the maize genome is an order of magnitude larger and contains ~19% more protein-coding genes [91,92]. Our identification of 41 GH28 family members in *Brachypodium *and 38 in sorghum reinforces the conclusion that grasses have smaller numbers of polygalacturonase genes (Figure 1 and additional file 8). Although small compared to *Arabidopsis*, the GH28 family in each of the grasses still consists of a substantial number of genes, possibly reflecting enzymatic functions associated with the pectin-rich middle lamella and the role of pectin during cell division.

**Figure 6:**
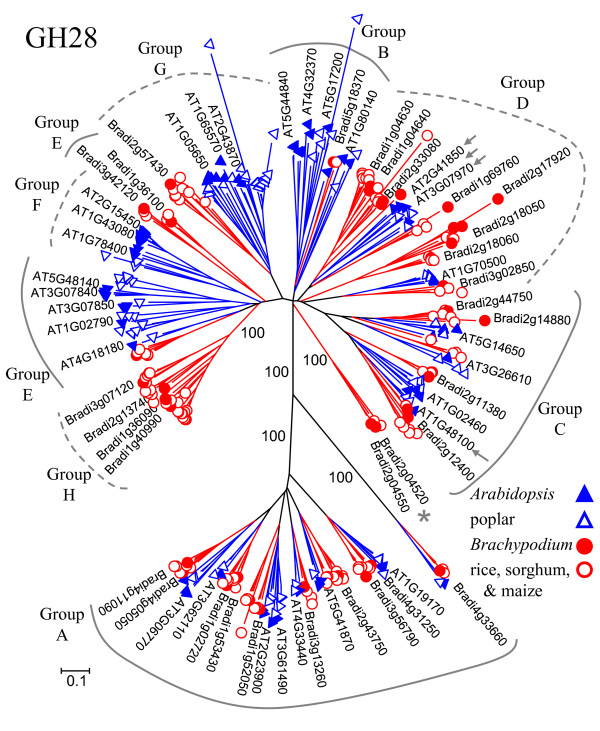
**The GH28 family of polygalacturonases**. The tree includes GH28 proteins from *Arabidopsis *(AT), rice, *Brachypodium *(Bradi), sorghum, poplar, and maize. Tree construction and color-coding (blue for eudicots and red for grasses) are as in Figure 2. For several branches, the percent of bootstrap replicates supporting the branch is indicated. Dotted and solid gray lines mark groups according to the designations of Penning *et al. *[90]. Small gray arrows indicate proteins corresponding to genes - *QRT2*, *ADPG1 *and *ADPG2 *in group D and At1g48100 in group C - characterized by Ogawa *et al. *[84]. A gray asterisk marks a clade of grass sequences that does not fall into any of the other groups. For clarity, names are shown only for selected *Arabidopsis *and *Brachypodium *genes. For complete branch labels and bootstrap values, see additional file 14.

To investigate the evolutionary history of the GH28 family, we constructed a phylogenetic tree based on protein sequences from *Arabidopsis*, rice, *Brachypodium*, sorghum, poplar, and maize (Figure 6 and additional file 14). The maize sequences were as identified by Penning *et al. *[90], except that the proteins encoded by AC210013.4 and AC231180.2 were omitted from the analysis, because they did not contain Pfam-predicted GH domains. For ease of reference, groups of GH28 proteins are labeled with the designations used by Penning *et al*. for *Arabidopsis*, rice, and maize [90]. These labels are included here to simplify comparisons across studies and do not necessarily correlate precisely with the clades of the six-species tree shown in Figure 6. For instance, while Group A has 100% bootstrap support, Group E as identified by Penning *et al. *[90] is split into two clades in our phylogenetic tree and is more appropriately regarded as part of the larger E/F/H clade (Figure 6 and additional file 14). The proteins encoded by *QRT2*, *ADPG1 *and *ADPG2 *fall into group D and the At1g48100-encoded protein into group C (Figure 6).

As has been noted previously [90], the increased number of GH28 genes in *Arabidopsis *appears to be due to expansion within groups, rather than the creation of entirely novel, eudicot-specific groups (Figure 6). For example, Group G and the less-cohesive Group E contain noticeably more *Arabidopsis *than *Brachypodium *members. There are, nevertheless, eudicot- and grass-specific clades; these include the eudicot-specific Group F and the grass-specific Group H recognized by Penning *et al. *[90] (Figure 6). In our analysis, Groups F and H have strong bootstrap support, 99 and 100%, respectively. These two groups also form part of a larger Group E/F/H, which is supported by 96% of 1,000 bootstrap replicates (Figure 6 and additional file 14).

A tree constructed from *Arabidopsis*, rice, and maize sequences had indicated that Group B is "*Arabidopsis *only" [90]. In our larger tree with additional sequences from *Brachypodium*, sorghum, and poplar, Group B was loosely clustered (50% bootstrap support) and contained one GH28 each from *Brachypodium *and sorghum (Figure 6 and additional file 14). Additionally, a tBLASTn search with the *Brachypodium *Group B member, Bradi5g18370, identified a wheat cDNA [GenBank: AK330487.1, *E*-value = 2^-119^] predicted to encode a protein with a GH28 domain. The presence of *Brachypodium *and sorghum sequences in Group B and the identification of a related wheat sequence suggest that monocots are under-represented in, rather than absent from, the GH28 Group B.

A further distinction between the three-species GH28 tree of Penning *et al. *[90] and the six-species tree presented here is the presence - in the larger tree - of a small, but very-well-supported, grass-specific clade comprised of two *Brachypodium*, one rice, and one sorghum sequence (indicated with an asterisk in Figure 6). Both of the *Brachypodium *genes, Bradi2g04520 and Bradi2g04550, have EST and Illumina transcriptome data supporting their expression. When the four grass sequences in this clade were used as queries in tBLASTn searches of GenBank, matches from five additional species (grape, castor bean, oilseed rape, avacado, and white spruce) were retrieved. However, none of these additional sequences fell into the same clade as the query sequences; this result is consistent with the apparent absence of the clade from species outside the Poaceae. As more genomes are sequenced and analyzed, it will be interesting to determine how widely distributed this clade actually is and whether it has a specialized function.

### The GH51 family

The GH51 family includes α-L-arabinofuranosidases, which cleave terminal, non-reducing α-L-arabinofuranose residues from arabinose-containing compounds [1]. The pectic polysaccharide rhamnogalacturonan I, found in dicot primary cell walls, and glucuronoarabinoxylan (GAX), the predominant hemicellulose in grass primary cell walls, both contain terminal arabinose residues in their side chains [82,83,89]. Correspondingly, GH51 family members are implicated in plant-cell-wall remodeling [4]. The barley GH51 protein AXAH-I, for example, released arabinose from sugar beet arabinan, wheat arabinoxylan, and larch wood arabinogalactan [93]. In contrast to barley AXAH-I, some GH51 members are bi-functional enzymes, exhibiting both α-L-arabinofuranosidase and β-D-xylosidase activities. *Arabidopsis *ARAF1, for instance, exhibits a preference for arabinose-containing substrates but can release both L-arabinose and D-xylose from wheat and rye arabinoxylan [94].

Arabinosidases have received particular attention for their contributions to pectin degradation during fruit ripening: while GH28 polygalacturonases cleave the pectin backbone, arabinosidases degrade pectin side chains [9]. During strawberry (*Fragaria × ananassa*) fruit development, α-L-arabinofuranosidase activity is prominent; in a comparison of two strawberry cultivars, the softer fruit of one cultivar also had higher α-L-arabinofuranosidase specific activity and higher transcript levels for three arabinofuranosidase genes, *FraAra1*, *2*, and *3 *[95]. Expression of GH51 family members is not, however, limited to fruits. The peach *ARF1 *gene, although initially identified based on its activity in fruit, is also expressed in leaves and roots [96].

*Arabidopsis ARAF1 *(At3g10740 or *ASD1*) is similarly broadly expressed, with *ARAF1 *transcripts detectable in roots, rosettes, stems, flowers, and siliques [97]. Analyses of plants transformed with an *ARAF1*-promoter-driven reporter indicated that *ARAF1 *is specifically expressed in tissues such as emerging lateral roots; the primary and developing secondary xylem of mature roots; the vasculature of cotyledons and leaves; and the phloem, cambium, and guard cells of the stem [97,98]. ARAF1 enzymatic activity is higher in young, growing stems than in mature stems, consistent with a possible cell-wall-remodeling function for ARAF1 [94]. Inmmunolocalization assays with the LM6 antibody, which binds arabinan epitopes, revealed localized increases in signal intensity in mutant *araf1 *stem and root tissues compared to the wild-type [98]. Conversely, wild-type stem sections exhibited markedly reduced signal intensity upon treatment with partially purified ARAF1 [98]. Together, these results suggest that ARAF1 acts on endogenous arabinose-containing polysaccharides in these tissues [98].

**Figure 7:**
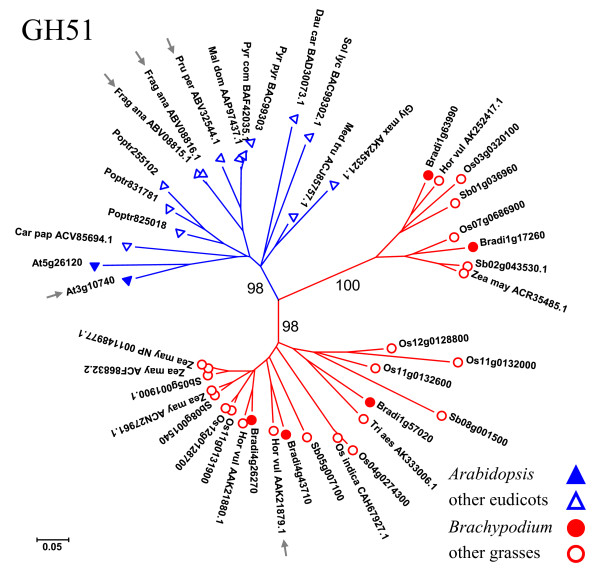
**The GH51 family of arabinofuranosidases**. The tree includes GH51 proteins from the following plants: *Arabidopsis *(AT), rice (Os), *Brachypodium *(Bradi), sorghum (Sb), and poplar (Poptr), as well as sequences from 10 other eudicots and 4 other grasses (labeled with a genus-plus-species abbreviation and the GenBank accession number). Tree construction and color-coding (blue for eudicots and red for grasses) are as in Figure 2. Small gray arrows indicate barley AXAH-I (Hor vul AAK21879.1), *Arabidopsis *ARAF1 (At3g10740), strawberry Ara1 and 2 (Frag ana ABV08815.1 and ABV08816.1), and peach ARF1 (Pru per ABV32544.1). *FraAra3 *appears to encode a truncated protein and was therefore omitted from our analysis. Car pap: *Carica papaya*; Dau car: *Daucus carota*; Fra ana: *Fragaria × ananassa*; Gly max: *Glycine max*; Hor vul: *Hordeum vulgare*; Mal dom: *Malus × domestica*; Med tru: *Medicago truncatula*; Os indica: *Oryza sativa *Indica Group; Pru per: *Prunus persica*; Pyr com: *Pyrus communis*; Pyr pyr: *Pyrus pyrifolia*; Sol lyc: *Solanum lycopersicum*; Tri aes: *Triticum aestivum*; Zea may: *Zea mays*. Common names for these species can be found in additional file 12. Poplar gene names are abbreviated; for the full names, see additional file 9. This tree is displayed in a rectangular format, with additional bootstrap values, in additional file 15.

In contrast to the large GH28 family, which is over-represented in eudicots, the smaller GH51 family is over-represented in grasses. Whereas *Arabidopsis *has two GH51 family members and poplar three, each of the grasses examined has approximately double this number: Rice has eight GH51 genes, *Brachypodium *five, and sorghum six (Figure 1 and additional file 8). A phylogenetic tree constructed from *Arabidopsis*, poplar, rice, *Brachypodium*, and sorghum GH51 proteins, as well as additional eudicot and grass GH51 sequences retrieved from the CAZy database and GenBank, is shown in Figure 7 and additional file 15. The grass sequences form two clades, both distinct from the single eudicot clade. This well-supported family structure (≥98% bootstrap values for the three major clades) suggests that there has been duplication and diversification within the grass lineage. The clear separation between eudicot and grass sequences likely reflects the differing architectures of eudicot and grass cell walls, underscoring the need for a grass model. The similarity between *Brachypodium *and other grasses with regard to the cell-wall-related GH5, GH28, and GH51 families illustrates that *Brachypodium *can meet this need.

### The GH13 family

The GH13 family is well-known as the α-amylase family [99]. It encompasses most of the starch-modifying enzymes with a wide range of substrate specificities and catalytic activities, such as α-amylases, pullulanases, isoamylases, cyclomaltodextrin glucanotransferases (CGTases), and branching enzymes [99,100]. Starch is the main component of cereal seeds and provides up to 80% of the calories consumed by humans. In addition, ethanol produced from starch is used as a transportation fuel [101]. Based on an analysis of catalytic domains, the GH13 family has been divided into 35 subfamilies, most of which represent a single catalytic activity; in those subfamilies with more than one catalytic activity, the activities are closely related [100]. Crystal structures have been reported for many GH13 family proteins. They have three conserved domains: domain A is the N-terminal, catalytic, (β/α)_8 _-barrel domain; domain B is a loop inserted in domain A; and domain C is a C-terminal, β-sandwich domain [99,102,103].

**Figure 8:**
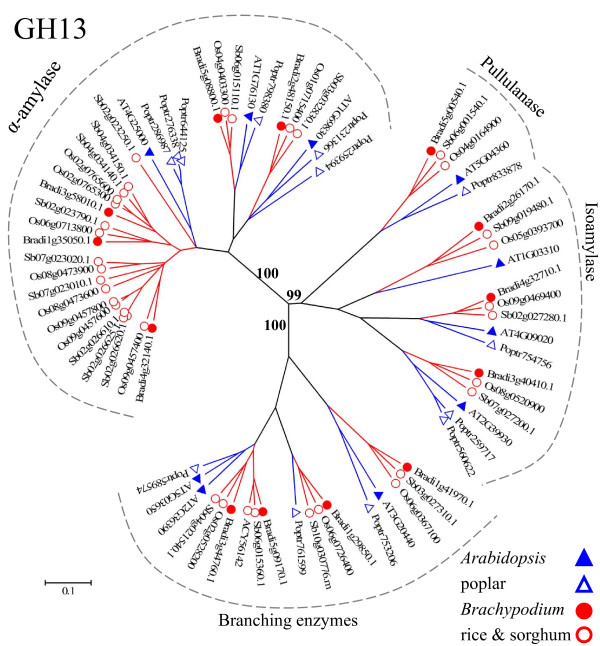
**The GH13 family of α-amylases, pullulanases, isoamylases, and branching enzymes**. The tree includes GH13 proteins from *Arabidopsis *(AT), rice (Os), *Brachypodium *(Bradi), sorghum (Sb), and poplar (Poptr). Tree construction and color-coding (blue for eudicots and red for grasses) are as in Figure 2. The three major clades are marked with dotted, gray lines and labeled with enzymatic activities. For complete bootstrap values, see additional file 16.

We identified 13 GH13 genes from *Brachypodium*, 19 from sorghum and 13 from poplar (additional files 3, 6, and 9). There are 10 and 18 members in *Arabidopsis *and rice, respectively [2]. A phylogenetic tree based on protein sequences was constructed to investigate the evolutionary history of the GH13 family (Figure 8 and additional file 16). A typical feature of this tree is that most of the branches have representatives from all species, suggesting broad conservation of the GH13 family in plants. The tree consists of three clades that are correlated with enzymatic activities: α-amylases (EC 3.2.1.1), branching enzymes (EC 2.4.1.18), and debranching enzymes including pullulanases (EC 3.2.1.41) and isoamylases (EC 3.2.1.68). All of the major branches of the tree are well-supported by bootstrap analysis (Figure 8 and additional file 16).

α-amylases (EC 3.2.1.1) are the best-studied of the GH13 enzymes, due to their wide industrial use. α-amylases catalyze the hydrolysis of internal α-D-(1,4)-glucosidic linkages in starch (amylose and amylopectin), glycogen, and related oligo- and polysaccharides, releasing maltodextrins, maltooligosaccharides and glucose [102]. There are three α-amylase genes (AtAmy1-3) in *Arabidopsis*, and they represented three groups in previous phylogenetic analyses of α-amylase proteins from multiple species [104,105]. The AtAmy1 (At4g25000) protein contains a signal sequence and was predicted to enter the secretory pathway, AtAmy3 (At1g69830) was identified as plastid-targeted, and AtAmy2 (At1g76130) does not appear to be targeted to any particular compartment of the cell [104]. Each enzyme is believed to have a different role in plants, given the putative subcellular localizations [105]. In our phylogenetic tree (Figure 8), the α-amylase clade is divided into two groups. One group containing AtAmy1 (At4g25000) includes a relatively large number of grass sequences. It has only 1 *Arabidopsis *and 3 poplar members, but 3 *Brachypodium*, 8 rice, and 9 sorghum members. Most of the additional rice and sorghum sequences are in clusters within the genome, indicating that the gene expansion was due to recent, local duplication, which is common for plant glycosidase- and glycosyltransferase-related genes [3]. The other group clusters AtAmy2 and AtAmy3 together with representatives from other species into two separate subgroups. Based on predictions of the TargetP program [106] (data not shown), these α-amylase genes, except two sorghum genes (Sb02g026625 and Sb03g032830) and two poplar genes (Poptr259394 and Poptr231366), encode proteins with the same subcellular location as the *Arabidopsis *member in the same group or subgroup.

Branching enzymes catalyze the formation of branch points by cleaving the α-1,4 linkage in polyglucans and reattaching the chain via an α-1,6-glucan linkage; conversely, debranching enzymes directly hydrolyze the α-1,6-glucosic linkages of polyglucans [107]. They are involved in starch biosynthesis in the cereal endosperm [107] and affect the eating and cooking quality of rice [108]. In the phylogenetic tree (Figure 8 and additional file 16), the branching and debranching enzymes form two clades. Each member is well-conserved between different species, except that poplar lacks one isoamylase and *Arabidopsis *lacks one branching enzyme representative (Figure 8). The presence of both grass and eudicot sequences in each of the major clades and most of the subclades of the GH13 family likely reflects the key roles of starch-modifying enzymes in plants.

## Conclusions

A decade ago, phylogenetic trees for plant GHs primarily showed that a handful of plant enzymes were more closely related to each other than to their bacterial counterparts. Since then, genome sequencing efforts have uncovered many more plant GH genes, whose cataloging can build the foundation for detailed functional studies. Now, with the availability of genome-wide analyses of GHs in *Arabidopsis*, rice, poplar, *Brachypodium*, and sorghum, it is possible to examine evolutionary histories and hypothesize about orthologous relationships within plant GH families. Our analysis showed that, while all angiosperms likely possess members of the same 34 GH families, there are significant differences between monocots and eudicots in the relationships within these families. These differences probably arose in part because of the compositional differences between grass and eudicot cell walls. However, by including additional species in our comparisons, we determined that several clades of GHs previously thought to contain only monocot or dicot proteins do, in fact, contain GHs from both eudicots and monocots. This highlights the importance of examining several species before making broad generalizations.

By defining the complement of *Brachypodium *GH genes, we set the stage for *Brachypodium *to be used as a grass model for investigations of the GHs and their diverse, associated functions. As with the eudicot model *Arabidopsis*, forward and reverse genetics combined with the phenotypic characterizations possible in a small, rapidly growing plant will help elucidate the *in planta *roles of GHs. Insights gained from *Brachypodium *will inform translational research studies, with applications for the improvement of cereal crops and bioenergy grasses.

## Abbreviations

CAZy: Carbohydrate-Active Enzymes; CBM: carbohydrate-binding module; CRD: cysteine-rich chitin-binding domain; CTE: carboxy-terminal extension; EST: expressed sequence tag; GH: glycoside hydrolase; NCBI: National Center for Biotechnology Information; PR: pathogenesis-related; XIP: xylanase inhibitor protein

## Authors' contributions

LT coordinated the overall analysis; compiled the overviews for the *Arabidopsis*, rice, poplar, *Brachypodium*, and sorghums GHs; retrieved poplar GH sequences; analyzed 90 GH families; performed phylogenetic analyses of the GH5, 28, and 51 families; wrote the accompanying text and edited the manuscript. JNB analyzed 8 GH families, performed phylogenetic analyses of the GH18 and 19 families, and contributed to the writing of the manuscript. JW analyzed 10 GH families, was instrumental in identifying sorghum GHs, performed phylogenetic analysis of the GH13 family, and contributed to the writing of the manuscript. XY performed searches for additional GH genes and contributed to the writing of the manuscript. GAT provided advice on the domain-based search and edited the manuscript. JPV conceived and designed the study, guided data analysis, and helped draft the manuscript. All authors read and approved the final manuscript.

## Supplementary Material

Additional file 1 **Rice GHs**. Rice GHs This file contains a FASTA-formatted list of protein sequences for rice GHs. Click here for file

Additional file 2 ***Arabidopsis *GHs**. *Arabidopsis *GHs This file contains a FASTA-formatted list of protein sequences for *Arabidopsis *GHs. Click here for file

Additional file 3 **Identification of *Brachypodium *GHs**. Identification of *Brachypodium *GHs This table contains the following information for each *Brachypodium *GH: the gene name, GH family assignment, Pfam-predicted domains, whether the *Brachypodium *protein matches a GH in rice or *Arabidopsis*, and the extent to which expression and splice-junction data support the gene model. Click here for file

Additional file 4 **Modified *Brachypodium *GH models**. Modified *Brachypodium *GH gene models This table lists the gene names, changes, and sequences associated with GH gene models which were modified or added relative to the v1.0 annotation. Click here for file

Additional file 5 ***Brachypodium *GHs**. *Brachypodium *GHs This file contains a FASTA-formatted list of protein sequences for *Brachypodium *GHs. Click here for file

Additional file 6 **Identification of Sorghum GHs**. Identification of Sorghum GHs This table contains the following information for each sorghum GH: the gene name; the GH family assignment; the best *E*-value for a match to a *Brachypodium*, rice, or *Arabidopsis *GH; whether there is a Pfam-predicted GH domain; and the protein sequence. Click here for file

Additional file 7 **Sorghum GHs**. Sorghum GHs This file contains a FASTA-formatted list of protein sequences for sorghum GHs. Click here for file

Additional file 8 **GH Summary**. GH Summary This table lists the number of GHs in each family in *Arabidopsis*, rice, *Brachypodium*, and sorghum. Click here for file

Additional file 9 **Poplar GHs**. Poplar GHs This table lists the abbreviated and full-length gene names, as well as the protein sequences, for selected poplar GHs analyzed in this study. Click here for file

Additional file 10 **GH18 Rectangular Tree**. GH18 Rectangular Tree This figure presents the same phylogenetic tree as Figure 3, but in a rectangular format, with complete bootstrap information and branch labels. The tree includes GH18 proteins from *Arabidopsis*, poplar, rice, *Brachypodium*, sorghum, and 18 other plants. Click here for file

Additional file 11 **Other Species**. Other Species Included in Trees This table lists the abbreviated names, full scientific names, common names, and classifications of species included in the phylogenetic trees. Click here for file

Additional file 12 **GH19 Rectangular Tree**. GH19 Rectangular Tree This figure presents the same phylogenetic tree as Figure 4, but in a rectangular format, with complete bootstrap information and branch labels. The tree includes GH19 proteins from *Arabidopsis*, poplar, rice, *Brachypodium*, and sorghum. Click here for file

Additional file 13 **GH5 Rectangular Tree**. GH5 Rectangular Tree This figure presents the same phylogenetic tree as Figure 5, but in a rectangular format, with complete bootstrap information and branch labels. The tree includes GH5 proteins from *Arabidopsis*, poplar, rice, *Brachypodium*, sorghum, and 23 other species. Click here for file

Additional file 14 **GH28 Rectangular Tree**. GH28 Rectangular Tree This figure presents the same phylogenetic tree as Figure 6, but in a rectangular format, with complete bootstrap information and branch labels. The tree includes GH28 proteins from *Arabidopsis*, poplar, rice, *Brachypodium*, sorghum, and maize. Click here for file

Additional file 15 **GH51 Rectangular Tree**. GH51 Rectangular Tree This figure presents the same phylogenetic tree as Figure 7, but in a rectangular format, with complete bootstrap information. The tree includes GH51 proteins from *Arabidopsis*, poplar, rice, *Brachypodium*, sorghum, and 14 other plants. Click here for file

Additional file 16 **GH13 Rectangular Tree**. GH13 Rectangular Tree This figure presents the same phylogenetic tree as Figure 8, but in a rectangular format, with complete bootstrap information. The tree includes GH13 proteins from *Arabidopsis*, poplar, rice, *Brachypodium*, and sorghum. Click here for file
