# Supplementary material for: Annotation and comparative analysis of the glycoside hydrolase genes in Brachypodium distachyon
Source: BMC Genomics. 2010 Oct 25;11:600. doi: 10.1186/1471-2164-11-600 (PMC3091745; doi:10.1186/1471-2164-11-600)
Supplement: Additional file 3 — Identification of Brachypodium GHs. Identification of Brachypodium GHs This table contains the following information for each Brachypodium GH: the gene name, GH family assignment, Pfam-predicted domains, whether the Brachypodium protein matches a GH in rice or Arabidopsis, and the extent to which expression and splice-junction data support the gene model. [file 1471-2164-11-600-S3.DOC]

| **Family #** | ***Brachypodium*,**  **# of members and assigned GH names** | **Gene** | **Match in rice? ^a^** | **Match in *Arabidopsis*? ^a^** | **Pfam GH domain** | ***E*-value for GH domain ^b^** | **Pfam carbohydrate binding module (CBM )** | ***E*-value for CBM ^b^** | **Expression support? ^c^** | **Splice junctions supported? ^d^** |
| --- | --- | --- | --- | --- | --- | --- | --- | --- | --- | --- |
| **1** | 30 |  |  |  |  |  |  |  |  |  |
|  | GH1_1 | Bradi1g10890 | yes | yes | Glyco_hydro_1: Glycosyl hydrolase family 1 | 1.50E-128 | ----- | ----- | yes | yes |
|  | GH1_2 | Bradi1g10920m **^e^** | yes | yes | Glyco_hydro_1 | 3.80E-185 | ----- | ----- | no | no |
|  | GH1_3 | Bradi1g10930 | yes | yes | Glyco_hydro_1 | 2.40E-204 | ----- | ----- | yes | yes |
|  | GH1_4 | Bradi1g10940 | yes | yes | Glyco_hydro_1 | 5.30E-207 | ----- | ----- | yes | yes |
|  | GH1_5 | Bradi1g19270 | yes | yes | Glyco_hydro_1 | 1.20E-207 | ----- | ----- | yes | yes |
|  | GH1_6 | Bradi1g33040 | yes | yes | Glyco_hydro_1 | 7.30E-211 | ----- | ----- | yes | yes |
|  | GH1_7 | Bradi1g42690 | yes | yes | Glyco_hydro_1 | 5.00E-217 | ----- | ----- | yes | yes |
|  | GH1_8 | Bradi1g70170 | yes | yes | Glyco_hydro_1 | 2.00E-215 | ----- | ----- | yes | partial |
|  | GH1_9 | Bradi2g09190 | yes | yes | Glyco_hydro_1;  Glyco_hydro_1 (2^nd^ of 2 such domains) | 5.20E-06;  8.50E-117 | ----- | ----- | some | partial |
|  | GH1_10 | Bradi2g09200 | yes | yes | Glyco_hydro_1 | 2.80E-137 | ----- | ----- | yes | yes |
|  | GH1_11 | Bradi2g27770 | yes | yes | Glyco_hydro_1 | 9.40E-177 | ----- | ----- | yes | yes |
|  | GH1_12 | Bradi2g57640 | yes | yes | Glyco_hydro_1 | 1.50E-210 | ----- | ----- | yes | yes |
|  | GH1_13 | Bradi2g59650 | yes | yes | Glyco_hydro_1 | 1.30E-165 | ----- | ----- | yes | yes |
|  | GH1_14 | Bradi2g59660 | yes | yes | Glyco_hydro_1 | 1.20E-172 | ----- | ----- | yes | yes |
|  | GH1_15 | Bradi3g00650 | yes | yes | Glyco_hydro_1 | 1.50E-206 | ----- | ----- | yes | yes |
|  | GH1_16 | Bradi3g40000 | yes | yes | Glyco_hydro_1 | 1.10E-172 | ----- | ----- | yes | yes |
|  | GH1_17 | Bradi3g40010 | yes | yes | Glyco_hydro_1 | 8.80E-180 | ----- | ----- | yes | yes |
|  | GH1_18 | Bradi3g45610 | yes | yes | Glyco_hydro_1 | 2.70E-145 | ----- | ----- | no | no |
|  | GH1_19 | Bradi3g45630 | yes | yes | Glyco_hydro_1 | 1.60E-148 | ----- | ----- | no | no |
|  | GH1_20 | Bradi3g45640 | yes | yes | Glyco_hydro_1 | 2.60E-150 | ----- | ----- | yes | yes |
|  | GH1_21 | Bradi3g45650 | yes | yes | Glyco_hydro_1 | 1.20E-181 | ----- | ----- | yes | yes |
|  | GH1_22 | Bradi4g08040 | yes | yes | Glyco_hydro_1 | 5.70E-178 | ----- | ----- | yes | none |
|  | GH1_23 | Bradi4g09920 | yes | yes | Glyco_hydro_1;  Glyco_hydro_1 (2^nd^ of 2 such domains) | 1.70E-08;  6.20E-20 | ----- | ----- | yes | yes |
|  | GH1_24 | Bradi4g34930 | yes | yes | Glyco_hydro_1 | 2.50E-152 | ----- | ----- | yes | yes |
|  | GH1_25 | Bradi4g34940 | yes | yes | Glyco_hydro_1 | 8.60E-171 | ----- | ----- | yes | yes |
|  | GH1_26 | Bradi4g34950 | yes | yes | Glyco_hydro_1 | 4.50E-163 | ----- | ----- | yes | yes |
|  | GH1_27 | Bradi5g13260 | yes | yes | Glyco_hydro_1 | 5.40E-218 | ----- | ----- | yes | partial |
|  | GH1_28 | Bradi5g13270 | yes | yes | Glyco_hydro_1 | 4.70E-200 | ----- | ----- | some | partial |
|  | GH1_29 | Bradi5g15530 | yes | yes | Glyco_hydro_1 | 9.40E-164 | ----- | ----- | no | no |
|  | GH1_30 | Bradi5g15540 | yes | yes | Glyco_hydro_1 | 3.60E-191 | ----- | ----- | yes | yes |
|  |  |  |  |  |  |  |  |  |  |  |
| **2** | 2 |  |  |  |  |  |  |  |  |  |
|  | GH2_1 | Bradi2g61010 | yes | yes | Glyco_hydro_2: Glycosyl hydrolases family 2, immunoglobulin-like beta-sandwich domain;  Glyco_hydro_2_C: Glycosyl hydrolases family 2, TIM barrel domain (E522, E587);  Bgal_small_N: Beta galactosidase small chain | 4.9E-96  6.4E-135  5.8E-77 | Glyco_hydro_2_N: Glycosyl hydrolases family 2, sugar binding domain | 4.9E-96 | yes | yes |
|  | GH2_2 | Bradi2g26600 | yes | yes | Glyco_hydro_2 | 0.001 | Glyco_hydro_2_N | 2.8E-08 | yes | all but 1 |
|  |  |  |  |  |  |  |  |  |  |  |
| **3** | 15 |  |  |  |  |  |  |  | yes | most |
|  | GH3_1 | Bradi1g08550 | yes | yes | Glyco_hydro_3: Glycosyl hydrolase family 3 N-terminal domain;  Glyco_hydro_3_C: Glycosyl hydrolase family 3 C-terminal domain | 4.2E-86;  1.5E-50 | ----- | ----- | yes | yes |
|  | GH3_2 | Bradi1g08560 | yes | yes | Glyco_hydro_3;  Glyco_hydro_3_C | 1.3E-85;  3.1E-63 | ----- | ----- | yes | most |
|  | GH3_3 | Bradi1g08570 | yes | yes | Glyco_hydro_3;  Glyco_hydro_3_C | 1.3E-88;  2.2E-63 | ----- | ----- | min. | no |
|  | GH3_4 | Bradi1g08580 | yes | yes | Glyco_hydro_3;  Glyco_hydro_3_C | 4.2E-89;  1.5E-66 | ----- | ----- | yes | most |
|  | GH3_5 | Bradi2g11410 | **yes** | yes | Glyco_hydro_3;  Glyco_hydro_3_C | 6.9E-45;  1.9E-72 | ----- | ----- | yes | most |
|  | GH3_6 | Bradi2g23720 | yes | yes | Glyco_hydro_3;  Glyco_hydro_3_C | 1.3E-85;  1.3E-52, fragment match | ----- | ----- | yes | some |
|  | GH3_7 | Bradi2g48460 | **yes** | yes | Glyco_hydro_3;  Glyco_hydro_3_C | 1.5E-45;  4.6E-80 | ----- | ----- | yes | most |
|  | GH3_8 | Bradi2g51280 | **yes** | yes | Glyco_hydro_3;  Glyco_hydro_3_C | 3E-90;  9.2E-60 | ----- | ----- | yes | yes |
|  | GH3_9 | Bradi3g59020 | **yes** | yes | Glyco_hydro_3;  Glyco_hydro_3_C | 5.5E-63;  8.9E-81 | ----- | ----- | min. | no |
|  | GH3_10 | Bradi4g16030 | yes | yes | Glyco_hydro_3;  Glyco_hydro_3_C | 1.1E-37;  3.4E-77 | ----- | ----- | yes | yes |
|  | GH3_11 | Bradi4g20200 | **yes** | yes | Glyco_hydro_3;  Glyco_hydro_3_C | 1.7E-57;  2.5E-79 | ----- | ----- | some | no |
|  | GH3_12 | Bradi4g20240 | **yes** | yes | Glyco_hydro_3;  Glyco_hydro_3_C | 4.4E-59;  3.1E-82 | ----- | ----- | no | no |
|  | GH3_13 | Bradi4g27700 | **yes** | yes | Glyco_hydro_3;  Glyco_hydro_3_C | 1.2E-43, fragment match;  2.5E-59 | ----- | ----- | yes | yes |
|  | GH3_14 | Bradi5g16560 | **yes** | yes | Glyco_hydro_3;  Glyco_hydro_3_C | 2E-60;  2.8E-71 | ----- | ----- | yes | yes |
|  | GH3_15 | Bradi5g23470 | **yes** | yes | Glyco_hydro_3;  Glyco_hydro_3_C | 4.2E-59;  4.8E-80 | ----- | ----- | yes | most |
|  |  |  |  |  |  |  |  |  |  |  |
| **4** | --- |  |  |  |  |  |  |  |  |  |
|  |  |  |  |  |  |  |  |  |  |  |
| **5** | 10 |  |  |  |  |  |  |  |  |  |
|  | GH5_1 | Bradi1g03210 | yes | 1.90E-99 | Cellulase: Cellulase (glycosyl hydrolase family 5); predicted active sites: E203, E322 | 9.2E-05 | ----- | ----- | yes | yes |
|  | GH5_2 | Bradi1g42770 | yes | yes | *Cellulase; predicted active sites: E221, E341* | *0.024 (not significant!)* | ----- | ----- | yes | yes |
|  | GH5_3 | Bradi2g31690 | yes | no | Cellulase | 2.1E-08, fragment match | ----- | ----- | yes | yes |
|  | GH5_4 | Bradi2g45790 | yes | yes | *Cellulase* | *0.002 (not significant!), fragment match* | ----- | ----- | yes | yes |
|  | GH5_5 | Bradi2g49680 | yes | yes | Cellulase; predicted active sites: E228, E349 | 0.00017 | ----- | ----- | yes | yes |
|  | GH5_6 | Bradi3g38500 | yes | no | Cellulase | 1.7E-06, fragment match | ----- | ----- | yes | most |
|  | GH5_7 | Bradi3g57920 | yes | yes | Cellulase; predicted active sites: E213, E330 | 7.9E-05 | ----- | ----- | no | no |
|  | GH5_8 | Bradi4g44340 | 9.30E-91 | 9.00E-93 | *Cellulase* | *0.023 (not significant!)* | ----- | ----- | yes | most |
|  | GH5_9 | Bradi5g13550 | yes | yes | Cellulase; predicted active sites: E209, E316 | 2.4e-06 | ----- | ----- | yes | most |
|  | GH5_10 | Bradi5g13560 | yes | yes | Cellulase; predicted active sites: E208, E315 | 1E-11, fragment match | Ricin-type beta-trefoil lectin domain | 2.8E-05, fragment match | yes | no |
|  |  |  |  |  |  |  |  |  |  |  |
| **6** | --- |  |  |  |  |  |  |  |  |  |
| **7** | --- |  |  |  |  |  |  |  |  |  |
| **8** | --- |  |  |  |  |  |  |  |  |  |
|  |  |  |  |  |  |  |  |  |  |  |
| **9** | 23 |  |  |  |  |  |  |  |  |  |
|  | GH9_1 | Bradi1g09460 | **yes** | yes | Glyco_hydro_9: Glycosyl hydrolase family 9 | 7.50E-219 | ----- | ----- | yes | yes |
|  | GH9_2 | Bradi1g35220 | yes | yes | Glyco_hydro_9 | 2.50E-197 | ----- | ----- | yes | yes |
|  | GH9_3 | Bradi1g44110 | yes | yes | Glyco_hydro_9 | 3.70E-256 | ----- | ----- | yes | yes |
|  | GH9_4 | Bradi1g44430 | yes | yes | Glyco_hydro_9 | 1.00E-243 | ----- | ----- | yes | yes |
|  | GH9_5 | Bradi1g54580 | yes | yes | Glyco_hydro_9 | 1.70E-247 | ----- | ----- | yes | none |
|  | GH9_6 | Bradi1g63560 | yes | yes | Glyco_hydro_9 | 4.30E-208 | ----- | ----- | yes | yes |
|  | GH9_7 | Bradi2g07150 | yes | yes | Glyco_hydro_9 | 6.70E-261 | CBM49: Carbohydrate binding domain CBM49 | 5.70E-21 | yes | yes |
|  | GH9_8 | Bradi2g11870 | yes | yes | Glyco_hydro_9 | 2.10E-281 | ----- | ----- | yes | min. |
|  | GH9_9 | Bradi2g32270 | yes | yes | Glyco_hydro_9 | 2.60E-256 | CBM49 | 2.20E-28 | yes | min. |
|  | GH9_10 | Bradi2g38360 | yes | yes | Glyco_hydro_9 | 5.30E-217 | ----- | ----- | min. | none |
|  | GH9_11 | Bradi3g04080 | yes | yes | Glyco_hydro_9 | 3.60E-225 | ----- | ----- | no | min. |
|  | GH9_12 | Bradi3g34790 | yes | yes | Glyco_hydro_9 | 4.40E-245 | ----- | ----- | yes | none |
|  | GH9_13 | Bradi3g36210 | yes | yes | Glyco_hydro_9 | 8.40E-239 | ----- | ----- | yes | none |
|  | GH9_14 | Bradi3g56870 | 6.90E-47 | 2.90E-39 | Glyco_hydro_9 | 1.70E-48 | ----- | ----- | no | none |
|  | GH9_15 | Bradi3g57190 | yes | yes | Glyco_hydro_9 | 1.30E-212 | ----- | ----- | yes | none |
|  | GH9_16 | Bradi3g59810 | yes | yes | Glyco_hydro_9 | 1.20E-244 | ----- | ----- | yes | yes |
|  | GH9_17 | Bradi4g29640 | yes | yes | Glyco_hydro_9 | 9.90E-246 | ----- | ----- | yes | yes |
|  | GH9_18 | Bradi4g33760 | yes | yes | Glyco_hydro_9 | 1.90E-256 | ----- | ----- | yes | none |
|  | GH9_19 | Bradi4g35970 | yes | yes | Glyco_hydro_9 | 4.70E-248 | ----- | ----- | yes | min. |
|  | GH9_20 | Bradi4g36240m | yes | yes | Glyco_hydro_9 | 1.40E-162 | ----- | ----- | yes | yes |
|  | GH9_21 | Bradi5g11310 | yes | yes | Glyco_hydro_9 | 3.20E-234 | ----- | ----- | yes | none |
|  | GH9_22 | Bradi5g14580 | yes | yes | Glyco_hydro_9 | 8.50E-189 | ----- | ----- | yes | yes |
|  | GH9_23 | Bradi5g26010 | yes | yes | Glyco_hydro_9 | 6.10E-233 | CBM49 | 2.50E-14 | yes | yes |
|  |  |  |  |  |  |  |  |  |  |  |
| **10** | 12 |  |  |  |  |  |  |  |  |  |
|  | GH10_1 | Bradi1g12710 | yes | yes | Glyco_hydro_10: Glycosyl hydrolase family 10 | 4.40E-65 | CBM_4_9: Carbohydrate binding domain;  CBM_4_9 | 9.3E-27;  1.1E-21 | yes | none |
|  | GH10_2 | Bradi1g27260 | yes | yes | Glyco_hydro_10 | 1.20E-41 | ----- | ----- | no | no |
|  | GH10_3 | Bradi1g28630 | yes | yes | Glyco_hydro_10 | 1.6E-63 | CBM_4_9; CBM_4_9; CBM_4_9; CBM_4_9; | 1.1E-22; 5E-22; 8.5E-20; 2.9E-19; | yes | yes |
|  | GH10_4 | Bradi1g68310 | yes | yes | Glyco_hydro_10 | 1.70E-44 | ----- | ----- | yes | yes |
|  | GH10_5 | Bradi1g71040 | yes | yes | Glyco_hydro_10 | 2.50E-31 | CBM_4_9 | 1.20E-08 | yes | yes |
|  | GH10_6 | Bradi1g71050 | yes | yes | Glyco_hydro_10 | 5.90E-11 | ----- | ----- | min. | none |
|  | GH10_7 | Bradi1g71080m | yes | yes | Glyco_hydro_10 | 3.90E-30 | CBM_4_9 | 1.70E-05 | no | no |
|  | GH10_8 | Bradi2g02320 | yes | yes | Glyco_hydro_10 | 2.10E-54 | ----- | ----- | yes | yes |
|  | GH10_9 | Bradi2g02330 | yes | yes | Glyco_hydro_10 | 5.60E-62 | ----- | ----- | min. | none |
|  | GH10_10 | Bradi3g24410m | yes | yes | Glyco_hydro_10 | 4.90E-22 | ----- | ----- | no | no |
|  | GH10_11 | Bradi5g04640 | yes | yes | Glyco_hydro_10 | 1.20E-37 | ----- | ----- | yes | yes |
|  | GH10_12 | Bradi1g56595m | **yes** | yes | Glyco_hydro_10 | 5.1E-29, fragment match | ----- | ----- | no | no |
|  |  |  |  |  |  |  |  |  |  |  |
| **11** | --- |  |  |  |  |  |  |  |  |  |
| **12** | --- |  |  |  |  |  |  |  |  |  |
|  |  |  |  |  |  |  |  |  |  |  |
| **13** | 13 |  |  |  |  |  |  |  |  |  |
|  | GH13_1 | Bradi1g29850 | **yes** | yes | Alpha-amylase: Alpha amylase, catalytic domain; predicted active sites: Y303, D338 | 5.5E-09, fragment match | CBM_48: Carbohydrate-binding module 48 (Isoamylase N-terminal domain);  *other domain:* Alpha-amylase_C: Alpha amylase, C-terminal all-beta domain | 1.6E-30, fragment match  1.3E-27, fragment match | yes | yes |
|  | GH13_2 | Bradi1g35050 | yes | yes | Alpha-amylase; predicted active sites: D206, E231, D317 | 4.4E-87 | *other domain:* Alpha-amyl_C2: Alpha-amylase C-terminal beta-sheet domain | 1.2E-35 | yes | yes |
|  | GH13_3 | Bradi1g41970 | **yes** | yes | Alpha-amylase; predicted active sites: Y466, D501 | 1.3E-09, fragment match | CBM_48;  *other domain:*  Alpha-amylase_C | 3.3E-06, fragment match;  3.5E-20, fragment match | yes | yes |
|  | GH13_4 | Bradi2g26170 | yes | yes | Alpha-amylase | 0.00013 | CBM_48 | 2E-12, fragment match | yes | none |
|  | GH13_5 | Bradi2g48150 | **yes** | yes | Alpha-amylase; predicted active sites: D647, E672, D755, Y526, D561 | 6.8E-49 | *other domain:* Alpha-amyl_C2 | 1.7E-30 | yes | yes |
|  | GH13_6 | Bradi3g40410 | **yes** | yes | Alpha-amylase; predicted active sites: Y303, D348 | 3.5E-19, fragment match | CBM_48 | 1.6E-33, fragment match | yes | yes |
|  | GH13_7 | Bradi3g44760 | **yes** | **yes** | Alpha-amylase; predicted active sites: Y377, D412 | 9.3E-15, fragment match | CBM_48;  *other domain:*  Alpha-amylase_C | 7E-30, fragment match;  6.4E-29, fragment match | yes | yes |
|  | GH13_8 | Bradi3g58010 | yes | yes | Alpha-amylase; predicted active sites: D203, E228, D314 | 2.1E-88 | *other domain*: Alpha-amyl_C2 | 7.8E-35 | yes | yes |
|  | GH13_9 | Bradi4g32140 | yes | yes | Alpha-amylase; predicted active sites: D208, E233, D316 | 1.2E-90 | *other domain*: Alpha-amyl_C2 | 2E-38 | yes | yes |
|  | GH13_10 | Bradi4g32710 | **yes** | yes | Alpha-amylase; predicted active sites: D413, Y292, D335 | 1.6E-20, fragment match | CBM_48 | 2.7E-18, fragment match | yes | yes |
|  | GH13_11 | Bradi5g00540 | **yes** | **yes** | Alpha-amylase; predicted active sites: D489, E526, D658 | 1.3E-05 | CBM_48 | 3E-27, fragment match | yes | yes |
|  | GH13_12 | Bradi5g08800 | yes | yes | Alpha-amylase; predicted active sites: D193, E218, D302, Y74, D109 | 2.1E-49 | *other domain:* Alpha-amyl_C2 | 1.8E-32 | yes | yes |
|  | GH13_13 | Bradi5g09170 | **yes** | **yes** | Alpha-amylase; predicted active sites: Y390, D425 | 1.8E-14, fragment match | CBM_48;  *other domain:* Alpha-amylase_C | 5.7E-31, fragment match;  3.9E-32, fragment match | yes | yes |
|  |  |  |  |  |  |  |  |  |  |  |
| **14** | 11 |  |  |  |  |  |  |  |  |  |
|  | GH14_1 | Bradi1g18390 | yes | yes | Glyco_hydro_14: Glycosyl hydrolase family 14 | 3.70E-44, fragment match | ----- | ----- | no | no |
|  | GH14_2 | Bradi1g25440 | yes | yes | Glyco_hydro_14; predicted active sites: E184, E378 | 8.60E-137 | ----- | ----- | yes | yes |
|  | GH14_3 | Bradi1g25450 | yes | yes | Glyco_hydro_14; predicted active sites: E316, E515 | 6.40E-141 | ----- | ----- | no | 2 of 4 |
|  | GH14_4 | Bradi1g62400 | yes | yes | Glyco_hydro_14 | 3.10E-31, fragment match | ----- | ----- | yes | yes |
|  | GH14_5 | Bradi1g75610 | yes | yes | Glyco_hydro_14; predicted active sites: E283, E481 | 3.30E-199 | ----- | ----- | yes | yes |
|  | GH14_6 | Bradi2g00820 | yes | yes | Glyco_hydro_14; predicted active sites: E633, E795 | 7.8E-101 | *other domain:*  Hydrolase: haloacid dehalogenase-like hydrolase | 8.8E-19, fragment match | yes | all but introns 2, 5, and 6 |
|  | GH14_7 | Bradi2g08190 | yes | yes | Glyco_hydro_14; predicted active sites: E151, E350 | 9.80E-102, fragment match | ----- | ----- | yes | yes |
|  | GH14_8 | Bradi3g02800 | yes | yes | Glyco_hydro_14; predicted active sites: E397, E591 | 1.3E-68, fragment match | *other domain:*  Plant protein of unknown function (DUF822) | 3.8E-65 | yes | all but 1 and 5 |
|  | GH14_9 | Bradi3g28210 | yes | yes | Glyco_hydro_14; predicted active sites: E241, E445 | 4.80E-178 | ----- | ----- | no | 1 |
|  | GH14_10 | Bradi3g33730 | yes | yes | Glyco_hydro_14; E253, E450 | 7.50E-245 | ----- | ----- | yes | yes |
|  | GH14_11 | Bradi4g38630 | yes | yes | Glyco_hydro_14; predicted active sites: E414, E610 | 3E-135 | *other domain:*  Plant protein of unknown function (DUF822) | 3.8E-38, fragment match | yes | all but intron 2 |
|  |  |  |  |  |  |  |  |  |  |  |
| **15** | --- |  |  |  |  |  |  |  |  |  |
|  |  |  |  |  |  |  |  |  |  |  |
| **16** | 30 |  |  |  |  |  |  |  |  |  |
|  | GH16_1 | Bradi1g01150 | yes | yes | Glyco_hydro_16: Glycosyl hydrolases family 16; predicted active sites: E120, E124 | 2.00E-75, fragment match | ----- | ----- | yes | none predicted |
|  | GH16_2 | Bradi1g09690 | yes | yes | Glyco_hydro_16; predicted active sites: E104, E108;  XET_C: Xyloglucan endo-transglycosylase (XET) C-terminus | 1.6E-92, fragment match; 4.5E-17 | ----- | ----- | yes | yes |
|  | GH16_3 | Bradi1g09700 | yes | yes | Glyco_hydro_16; predicted active sites: E104, E108;  XET_C | 1.6E-92, fragment match;  4.5E-17 | ----- | ----- | yes | yes |
|  | GH16_4 | Bradi1g25850 | yes | yes | Glyco_hydro_16; predicted active sites: E115, E119;  XET_C | 1.3E-88, fragment match; 3.3E-23 | ----- | ----- | yes | Junction 3 is covered; 1 and 2 are not. |
|  | GH16_5 | Bradi1g33810 | yes | yes | Glyco_hydro_16; predicted active sites: E97, E101;  XET_C | 2.6E-106, fragment match;  1E-30 | ----- | ----- | yes | yes |
|  | GH16_6 | Bradi1g33820 | yes | yes | Glyco_hydro_16;  XET_C | 4.4E-87, fragment match; 1.7E-16 | ----- | ----- | yes | yes |
|  | GH16_7 | Bradi1g33830 | yes | yes | Glyco_hydro_16; predicted active sites: E97, E101;  XET_C | 2.6E-99, fragment match; 3.1E-27 | ----- | ----- | yes | yes |
|  | GH16_8 | Bradi1g33840 | yes | yes | Glyco_hydro_16; predicted active sites: E100, E104;  XET_C | 9.5E-111, fragment match;  7E-24 | ----- | ----- | yes | yes |
|  | GH16_9 | Bradi1g44780 | 3E-35 | 1E-29 | Glyco_hydro_16;  XET_C | 0.00063;  2.6E-22 | ----- | ----- | no | no |
|  | GH16_10 | Bradi1g68590 | yes | yes | Glyco_hydro_16; predicted active sites: E121, E125;  XET_C | 4.5E-88, fragment match; 6.7E-13 | ----- | ----- | yes | yes |
|  | GH16_11 | Bradi1g71940 | 3E-29 | 1E-26 | Glyco_hydro_16;  XET_C | 0.00006;  7.9E-10 | ----- | ----- | no | no |
|  | GH16_12 | Bradi1g77360 | yes | yes | Glyco_hydro_16; predicted active sites: E95, E99 | 3.80E-31, fragment match | ----- | ----- | yes | none predicted |
|  | GH16_13 | Bradi1g77990 | yes | yes | Glyco_hydro_16; predicted active sites: E137, E141;  XET_C | 6.7E-86, fragment match; 4.9E-13 | ----- | ----- | yes | yes |
|  | GH16_14 | Bradi3g02700 | yes | yes | Glyco_hydro_16; predicted active sites: E108, E112;  XET_C | 1.4E-84, fragment match; 1.8E-14 | ----- | ----- | no | no |
|  | GH16_15 | Bradi3g10290 | yes | yes | Glyco_hydro_16; predicted active sites: E99, E103;  XET_C | 4.4E-97, fragment match;  4E-10 | ----- | ----- | yes | yes |
|  | GH16_16 | Bradi3g10310 | yes | yes | Glyco_hydro_16; predicted active sites: E102, E106 | 8.10E-88, fragment match | ----- | ----- | yes | yes |
|  | GH16_17 | Bradi3g18590 | yes | yes | Glyco_hydro_16; predicted active sites: E104, E108;  XET_C | 8.4E-89, fragment match; 9.1E-23 | ----- | ----- | yes | yes |
|  | GH16_18 | Bradi3g18600 | yes | yes | Glyco_hydro_16; predicted active sites: E105, E109;  XET_C | 3.5E-86, fragment match; 4.3E-19 | ----- | ----- | yes | yes |
|  | GH16_19 | Bradi3g18610 | yes | yes | Glyco_hydro_16;  XET_C | 3.2E-72, fragment match;  2E-10 | ----- | ----- | yes | 1st no, 2nd yes |
|  | GH16_20 | Bradi3g18690 | yes | yes | Glyco_hydro_16; predicted active sites: E106, E110;  XET_C | 5.7E-94, fragment match;  6E-13 | ----- | ----- | yes | yes |
|  | GH16_21 | Bradi3g21340m | yes | yes | Glyco_hydro_16;  XET_C | 0.0000000000016, fragment match; 6.3E-13 | ----- | ----- | yes | only 3rd |
|  | GH16_22 | Bradi3g31770 | 5E-41 | 2E-26 | Glyco_hydro_16;  XET_C | 0.00000000000069, fragment match; 8.1E-05 | ----- | ----- | yes | yes |
|  | GH16_23 | Bradi3g34230 | yes | no | Glyco_hydro_16 | 4.10E-23, fragment match | ----- | ----- | yes | 1st but not 2^nd^ |
|  | GH16_24 | Bradi4g16990 | yes | yes | Glyco_hydro_16; predicted active sites: E107, E111;  XET_C | 8.9E-99, fragment match; 7.4E-27 | ----- | ----- | yes | yes |
|  | GH16_25 | Bradi4g29710 | yes | no | *Glycosyl hydrolases family 16* | *0.43 (not significant!)* | ----- | ----- | yes | yes |
|  | GH16_26 | Bradi5g20720 | yes | yes | Glyco_hydro_16; predicted active sites: E184, E188;  XET_C | 6.1E-87, fragment match;  5E-14 | ----- | ----- | yes | yes |
|  | GH16_27 | Bradi5g20730 | 6E-50 | 4E-29 | Glyco_hydro_16;  XET_C | 0.0000000013, fragment match; 8.1E-14 | ----- | ----- | yes | no |
|  | GH16_28 | Bradi5g20740 | yes | yes | Glyco_hydro_16;  XET_C | 6.4E-21, fragment match; 1.3E-17 | ----- | ----- | no | no |
|  | GH16_29 | Bradi5g22910 | yes | yes | Glyco_hydro_16;  XET_C | 0.000000071, fragment match;  5E-15 | ----- | ----- | yes | yes |
|  | GH16_30 | Bradi1g27870m | yes | yes | Glyco_hydro_16, predicted active sites: 122,124,126;  XET_C | 3.3E-52;  8.1E-12 | ----- | ----- | yes | modified |
|  |  |  |  |  |  |  |  |  |  |  |
| **17** | 54 |  |  |  |  |  |  |  |  |  |
|  | GH17_1 | Bradi1g06050 | **yes** | yes | Glyco_hydro_17: Glycosyl hydrolases family 17 | 4.20E-75 | X8: X8 domain (possibly involved in carbohydrate binding) | 8.70E-34 | yes | yes |
|  | GH17_2 | Bradi1g12810 | **yes** | yes | Glyco_hydro_17 | 5.10E-111 | X8 | 5.80E-44 | yes | yes |
|  | GH17_3 | Bradi1g13230m | **yes** | yes | Glyco_hydro_17 | 1.90E-74 | X8 | 2.90E-37 | yes | yes |
|  | GH17_4 | Bradi1g23640 | **yes** | yes | Glyco_hydro_17 | 3.80E-85 | X8 | 6.70E-39 | yes | yes |
|  | GH17_5 | Bradi1g25520m | yes | yes | Glyco_hydro_17 | 2.8-86 | X8;  X8 (2^nd^ of 2 such domains) | 1.1E-28, fragment match;  1.0E-30, fragment match | yes | yes |
|  | GH17_6 | Bradi1g25530 | yes | yes | Glyco_hydro_17 | 2.80E-102, fragment match | X8;  X8 (2^nd^ of 2 such domains) | 5.90E-48, fragment match;  4.80E-47, fragment match | yes | yes |
|  | GH17_7 | Bradi1g26510 | **yes** | yes | Glyco_hydro_17 | 2.40E-79 | X8 | 1.60E-50 | yes | yes |
|  | GH17_8 | Bradi1g36460 | yes | yes | Glyco_hydro_17 | 4.10E-102 | ----- | ----- | yes | yes |
|  | GH17_9 | Bradi1g37160 | **yes** | yes | Glyco_hydro_17 | 1.50E-72, fragment match | X8 | 2.20E-32 | no | no |
|  | GH17_10 | Bradi1g50080 | yes | 1.00E-99 | Glyco_hydro_17 | 7.60E-109 | ----- | ----- | yes | yes |
|  | GH17_11 | Bradi1g53590 | yes | yes | Glyco_hydro_17 | 2.20E-105 | ----- | ----- | yes | no |
|  | GH17_12 | Bradi1g56270 | **yes** | yes | Glyco_hydro_17 | 2.20E-81 | X8 | 3.40E-38 | yes | no |
|  | GH17_13 | Bradi1g60410 | yes | yes | Glyco_hydro_17 | 5.80E-78, fragment match | X8 | 1.20E-32 | no | none predicted |
|  | GH17_14 | Bradi1g61320 | yes | 6.00E-43 | Glyco_hydro_17 | 1.20E-20, fragment match | X8 | 1.20E-41 | yes | yes |
|  | GH17_15 | Bradi1g65200 | yes | 3.00E-94 | Glyco_hydro_17 | 3.80E-66 | ----- | ----- | yes | no |
|  | GH17_16 | Bradi1g68450 | yes | yes | Glyco_hydro_17 | 4.90E-117 | ----- | ----- | yes | no |
|  | GH17_17 | Bradi1g69020 | yes | yes | Glyco_hydro_17 | 3.50E-104 | ----- | ----- | yes | yes |
|  | GH17_18 | Bradi1g69610 | **yes** | **yes** | Glyco_hydro_17 | 6.40E-101 | X8 | 2.70E-46 | yes | yes |
|  | GH17_19 | Bradi2g18420 | **yes** | yes | Glyco_hydro_17 | 1.30E-83 | X8 | 3.20E-34 | yes | no |
|  | GH17_20 | Bradi2g18700 | **yes** | yes | Glyco_hydro_17 | 4.80E-74 | X8 | 8.80E-35 | yes | no |
|  | GH17_21 | Bradi2g23940 | yes | yes | Glyco_hydro_17 | 1.30E-110 | ----- | ----- | yes | yes |
|  | GH17_22 | Bradi2g27140 | yes | 3.00E-73 | Glyco_hydro_17 | 1.60E-186 | ----- | ----- | yes | yes |
|  | GH17_23 | Bradi2g49330 | **yes** | yes | Glyco_hydro_17 | 3.00E-129 | X8 | 3.20E-49 | yes | yes |
|  | GH17_24 | Bradi2g55690 | yes | yes | Glyco_hydro_17 | 1.20E-115 | ----- | ----- | yes | yes |
|  | GH17_25 | Bradi2g60490 | yes | 3.00E-69 | Glyco_hydro_17 | 3.10E-206 | ----- | ----- | yes | yes |
|  | GH17_26 | Bradi2g60500 | yes | 3.00E-68 | Glyco_hydro_17 | 1.30E-156 | ----- | ----- | yes | none predicted |
|  | GH17_27 | Bradi2g60560 | yes | 4.00E-65 | Glyco_hydro_17 | 3.00E-175 | ----- | ----- | no | no |
|  | GH17_28 | Bradi3g03520 | **yes** | yes | Glyco_hydro_17 | 8.90E-85 | X8 | 2.90E-37 | yes | yes |
|  | GH17_29 | Bradi3g18220 | **yes** | yes | Glyco_hydro_17 | 2.50E-96 | X8 | 2.10E-38 | yes | no |
|  | GH17_30 | Bradi3g20770 | yes | yes | Glyco_hydro_17 | 1.10E-118 | ----- | ----- | yes | yes |
|  | GH17_31 | Bradi3g40910 | yes | yes | Glyco_hydro_17 | 3.20E-116 | X8;  X8 | 6.10E-49;  8.60E-43, fragment match | no | no |
|  | GH17_32 | Bradi3g44910 | yes | yes | Glyco_hydro_17 | 5.10E-114 | ----- | ----- | yes | yes |
|  | GH17_33 | Bradi3g57610 | **yes** | yes | Glyco_hydro_17 | 6.00E-131 | X8 | 1.10E-48 | yes | yes |
|  | GH17_34 | Bradi4g09230 | yes | yes | Glyco_hydro_17 | 8.20E-82 | X8 | 2.20E-49 | yes | yes |
|  | GH17_35 | Bradi4g15460 | yes | yes | Glyco_hydro_17 | 8.10E-69 | X8 | 6.20E-32 | yes | yes |
|  | GH17_36 | Bradi4g34390 | **yes** | yes | Glyco_hydro_17 | 1.90E-113 | X8 | 2.60E-41 | yes | yes |
|  | GH17_37 | Bradi4g36190 | yes | yes | Glyco_hydro_17 | 5.80E-110 | ----- | ----- | yes | none predicted |
|  | GH17_38 | Bradi5g09330 | yes | yes | Glyco_hydro_17 | 2.00E-111 | ----- | ----- | yes | only 3^rd^ and 4^th^ junctions |
|  | GH17_39 | Bradi5g12140 | **yes** | yes | Glyco_hydro_17 | 2.90E-102 | X8 | 3.20E-20, fragment match | none | none |
|  | GH17_40 | Bradi5g26470m | yes | 1.00E-87 | Glyco_hydro_17 | 1.00E-62, fragment match | X8 | 2.6E-24 | yes | yes |
|  | GH17_41 | Bradi1g15295m | yes | 8.60E-73 | Glyco_hydro_17 | 1.10E-158, fragment match | ----- | ----- | some | no |
|  | GH17_42 | Bradi1g55205m | 5.00E-85 | 8.00E-57 | Glyco_hydro_17 | 4.80E-61, fragment match | ----- | ----- | some | no |
|  | GH17_43 | Bradi2g22222m | yes | 3.10E-68 | Glyco_hydro_17 | 2.50E-172, fragment match | ----- | ----- | yes | no |
|  | GH17_44 | Bradi2g22223m | yes | 1.30E-67 | Glyco_hydro_17 | 1.50E-173, fragment match | ----- | ----- | yes | yes,  2 splices |
|  | GH17_45 | Bradi2g22225m | yes | 5.50E-71 | Glyco_hydro_17 | 2.60E-174, fragment match | ----- | ----- | yes | yes |
|  | GH17_46 | Bradi2g22228m | 2.00E-96 | 1.30E-50 | Glyco_hydro_17 | 2.10E-110 | ----- | ----- | some | no |
|  | GH17_47 | Bradi2g43055m | yes | 3.00 E-70 | Glyco_hydro_17 | 1.50E-180, fragment match | ----- | ----- | yes | no |
|  | GH17_48 | Bradi2g52569m | yes | 2.50E-66 | Glyco_hydro_17 | 2.10E-169, fragment match | ----- | ----- | yes | no |
|  | GH17_49 | Bradi2g60445m | yes | 2.10E-71 | Glyco_hydro_17 | 6.50E-195, fragment match | ----- | ----- | yes | yes |
|  | GH17_50 | Bradi2g60532m | yes | 8.90E-71 | Glyco_hydro_17; predicted active sites: E263, E321 | 3.20E-179, fragment match | ----- | ----- | some | no |
|  | GH17_51 | Bradi2g60536m | 5.00E-82 | 3.60E-51 | Glyco_hydro_17; predicted active sites: E189, E246 | 1.20E-145, fragment match | ----- | ----- | min. | no |
|  | GH17_52 | Bradi2g60538m | yes | 3.70E-65 | Glyco_hydro_17; predicted active sites: | 1.60E-180, fragment match | ----- | ----- | min. | no |
|  | GH17_53 | Bradi3g07385m | yes | yes | Glyco_hydro_17; predicted active sites: E321, E384 | 3.70E-114, fragment match | ----- | ----- | yes | no |
|  | GH17_54 | Bradi3g33279m | 2.00E-95 | 1.90E-68 | Glyco_hydro_17; predicted active sites: E219, E282 | 1.00E-58, fragment match | ----- | ----- | yes | no |
|  |  |  |  |  |  |  |  |  |  |  |
| **18** | 14 |  |  |  |  |  |  |  |  |  |
|  | GH18_1 | Bradi2g45610 | yes | yes | Glyco_hydro_18: Glycosyl hydrolases family 18 | 8.40E-52 | ----- | ----- | min. | none |
|  | GH18_2 | Bradi2g47070 | yes | yes | Glyco_hydro_18 | 3.8E-36 | *other domain:*  Protein tyrosine kinase | 5.1e-17, fragment match | none | none |
|  | GH18_3 | Bradi2g55610 | yes | yes | Glyco_hydro_18 | 9.40E-51 | ----- | ----- | none | none |
|  | GH18_4 | Bradi2g55620 | yes | yes | Glyco_hydro_18 | 1.90E-49 | ----- | ----- | none | none |
|  | GH18_5 | Bradi2g55630 | yes | yes | Glyco_hydro_18 | 4.60E-48 | ----- | ----- | none | none |
|  | GH18_6 | Bradi3g26810 | yes | no | Glyco_hydro_18 | 1.20E-12, fragment match | ----- | ----- | no | none |
|  | GH18_7 | Bradi3g26840 | yes | no | Glyco_hydro_18 | 0.0001, fragment match | ----- | ----- | yes | none |
|  | GH18_8 | Bradi3g26850 | yes | no | Glyco_hydro_18 | 4.70E-08, fragment match | ----- | ----- | yes | none |
|  | GH18_9 | Bradi4g07560 | yes | yes | Glyco_hydro_18;  Glyco_hydro_18 (2^nd^ of 2 fragments) | 2.1E-09, fragment match; 0.0002, fragment match | ----- | ----- | none | none |
|  | GH18_10 | Bradi4g09420 | yes | yes | Glyco_hydro_18 | 0.0004, fragment match | ----- | ----- | none | yes |
|  | GH18_11 | Bradi4g09430 | yes | yes | Glyco_hydro_18 | 1.60E-24 | ----- | ----- | yes | none |
|  | GH18_12 | Bradi4g40110 | yes | yes | Glyco_hydro_18 | 9.40E-24 | ----- | ----- | yes | none |
|  | GH18_13 | Bradi5g07230 | yes | yes | Glyco_hydro_18 | 7.70E-51 | ----- | ----- | min. | yes |
|  | GH18_14 | Bradi2g43755m | yes | 2.10E-85 | Glyco_hydro_18 | 3.2E-51, fragment match | ----- | ----- | min. | none |
|  |  |  |  |  |  |  |  |  |  |  |
| **19** | 10 |  |  |  |  |  |  |  |  |  |
|  | GH19_1 | Bradi1g29880 | yes | yes | Glyco_hydro_19: Chitinase class I | 1.90E-174 | ----- | ----- | yes | none |
|  | GH19_2 | Bradi1g29890 | yes | yes | Glyco_hydro_19 | 5.7E-183 | Chitin_bind_1: Chitin recognition protein | 9.6E-21 | yes | none |
|  | GH19_3 | Bradi2g11140 | yes | yes | Glyco_hydro_19 | 1.50E-133 | ----- | ----- | yes | none |
|  | GH19_4 | Bradi2g26000 | yes | yes | Glyco_hydro_19 | 1.3E-158 | Chitin_bind_1;  Chitin_bind_1 (2^nd^ of 2 such domains) | 9.8E-20;  3.9E-17 | yes | none |
|  | GH19_5 | Bradi2g36780 | yes | yes | Glyco_hydro_19 | 7.60E-135 | ----- | ----- | no | none |
|  | GH19_6 | Bradi2g47190 | yes | yes | Glyco_hydro_19 | 3.3E-20 | Chitin_bind_1 | 3.3E-20 | no | none |
|  | GH19_7 | Bradi2g47210 | yes | yes | Glyco_hydro_19 | 6.8E-183 | Chitin_bind_1 | 3.5E-21 | yes | none |
|  | GH19_8 | Bradi3g32340 | yes | yes | Glyco_hydro_19 | 4.30E-141 | ----- | ----- | yes | yes |
|  | GH19_9 | Bradi3g40320 | yes | yes | Glyco_hydro_19 | 8.50E-67 | ----- | ----- | yes | yes |
|  | GH19_10 | Bradi3g48230 | yes | yes | Glyco_hydro_19 | 3.1E-106 | Chitin_bind_1 | 1.5E-11 | yes | yes |
|  |  |  |  |  |  |  |  |  |  |  |
| **20** | 6 |  |  |  |  |  |  |  |  |  |
|  | GH20_1 | Bradi1g23740 | yes | yes | Glyco_hydro_20: Glycosyl hydrolase family 20, catalytic domain (contains a TIM barrel fold) | 5.5E-129 | *other domain:*  Glyco_hydro_ 20b: Glycosyl hydrolase family 20, domain 2 | 1.2E-13, fragment match | yes | none |
|  | GH20_2 | Bradi1g69660 | yes | yes | Glyco_hydro_20 | 1.4E-126 | *other domain:*  Glyco_hydro_ 20b | 5.8E-15, fragment match | yes | yes |
|  | GH20_3 | Bradi2g25310 | yes | yes | Glyco_hydro_20 | 1.3E-123 | *other domain:*  Glyco_hydro_ 20b | 3.3E-16, fragment match | yes | yes |
|  | GH20_4 | Bradi2g37460 | yes | yes | Glyco_hydro_20 | 3.6E-104 | *other domain:*  Glyco_hydro_ 20b | 1.8E-20, fragment match | yes | yes |
|  | GH20_5 | Bradi2g57420 | yes | yes | Glyco_hydro_20 | 6.2E-117 | *other domain:*  Glyco_hydro_ 20b | 1.5E-16, fragment match | yes | yes |
|  | GH20_6 | Bradi3g44610 | yes | yes | Glyco_hydro_20 | 3.3E-112 | *other domain:*  Glyco_hydro_ 20b | 3.7E-16, fragment match | yes | yes |
|  |  |  |  |  |  |  |  |  |  |  |
| **(21) ^f^** | --- |  |  |  |  |  |  |  |  |  |
| **22** | --- |  |  |  |  |  |  |  |  |  |
| **23** | --- |  |  |  |  |  |  |  |  |  |
| **24** | --- |  |  |  |  |  |  |  |  |  |
| **25** | --- |  |  |  |  |  |  |  |  |  |
| **26** | --- |  |  |  |  |  |  |  |  |  |
|  |  |  |  |  |  |  |  |  |  |  |
| **27** | 5 |  |  |  |  |  |  |  |  |  |
|  | GH27_1 | Bradi1g17730 | yes | yes | Melibiase | 7.50E-14 | ----- | ----- | yes | yes |
|  | GH27_2 | Bradi2g13520 | yes | yes | -----  Note: Bradi2g13520 and Bradi2g13530 were considered to be GHs because, although they lack a significant match to a Pfam GH domain, they are highly similar to rice and *Arabidopsis* GH27 family members which also lack a predicted Pfam GH domain. | ----- | Ricin_B_lectin | 2.90E-05, fragment match | yes | yes |
|  | GH27_3 | Bradi2g13530 | yes | yes | ----- | ----- | ----- | ----- | yes | yes |
|  | GH27_4 | Bradi3g29800 | yes | yes | Melibiase | 1.90E-06 | ----- | ----- | yes | all but 3 and 5 |
|  | GH27_5 | Bradi3g29810 | yes | yes | Melibiase | 7.50E-14 | ----- | ----- | yes | yes |
|  |  |  |  |  |  |  |  |  |  |  |
| **28** | 41 |  |  |  |  |  |  |  |  |  |
|  | GH28_1 | Bradi1g02720 | yes | yes | Glyco_hydro_28: Glycosyl hydrolases family 28 | 1.8E-26, fragment match | ----- | ----- | yes | most |
|  | GH28_2 | Bradi1g04610 | yes | yes | Glyco_hydro_28;  predicted active sites: D229, H252 | 3.7E-145 | ----- | ----- | min. | none predicted |
|  | GH28_3 | Bradi1g04630 | yes | yes | Glyco_hydro_28;  predicted active sites: D233, H256 | 9E-136 | ----- | ----- | yes | none predicted |
|  | GH28_4 | Bradi1g04640 | yes | 4.50E-80 | Glyco_hydro_28;  Glyco_hydro_28 (2nd of 2 adjacent GH28 fragments) | 1.7E-59, fragment match;  1.9E-28, fragment match | ----- | ----- | min. | no |
|  | GH28_5 | Bradi1g36090  (The predicted protein sequence is identical for Bradi1g36090  and Bradi1g36110.) | yes | 1.80E-85 | Glyco_hydro_28;  predicted active sites: D233, H256 | 1.1E-149 | ----- | ----- | yes | no |
|  | GH28_6 | Bradi1g36100 | yes | 1.40E-85 | Glyco_hydro_28;  predicted active sites: D220, H243 | 3.8E-116 | ----- | ----- | no | no |
|  | GH28_7 | Bradi1g36110  (The predicted protein sequence is identical for Bradi1g36090  and Bradi1g36110.) | yes | 1.80E-85 | Glyco_hydro_28;  predicted active sites: D233, H256 | 1.1E-149 | ----- | ----- | min. | no |
|  | GH28_8 | Bradi1g40990 | yes | 1.80E-85 | Glyco_hydro_28;  predicted active sites: D250, H273 | 8.2E-165 | ----- | ----- | yes | no |
|  | GH28_9 | Bradi1g51310 | yes | 1.10E-96 | Glyco_hydro_28;  predicted active sites: D281, H304 | 6.1E-133 | ----- | ----- | no | none predicted |
|  | GH28_10 | Bradi1g52050 | yes | yes | Glyco_hydro_28 | 1.1E-19, fragment match | ----- | ----- | yes | yes |
|  | GH28_11 | Bradi1g53430 | yes | yes | Glyco_hydro_28 | 5E-15, fragment match | ----- | ----- | yes | yes |
|  | GH28_12 | Bradi1g69760 | yes | 1.30E-84 | Glyco_hydro_28;  predicted active sites: D291, H314 | 2.1E-109, fragment match | ----- | ----- | min. | min. |
|  | GH28_13 | Bradi1g76890 | yes | yes | Glyco_hydro_28 | 9.9E-22, fragment match | ----- | ----- | yes | yes |
|  | GH28_14 | Bradi2g04520 | yes | 2.60E-72 | Glyco_hydro_28;  predicted active sites: D232, H255 | 3.6E-90 | ----- | ----- | yes | most |
|  | GH28_15 | Bradi2g04550 | yes | 6.60E-74 | Glyco_hydro_28;  predicted active sites: D231, H254 | 3.7E-97 | ----- | ----- | yes | most |
|  | GH28_16 | Bradi2g11380 | yes | yes | Glyco_hydro_28;  predicted active sites: D306, H329 | 2.3E-107, fragment match | ----- | ----- | yes | most |
|  | GH28_17 | Bradi2g12400 | yes | yes | Glyco_hydro_28;  predicted active sites: D419, H442 | 1.7E-102, fragment match | ----- | ----- | min. | no |
|  | GH28_18 | Bradi2g13740 | yes | 7.40E-82 | Glyco_hydro_28 | 6.5E-132 | ----- | ----- | no | no |
|  | GH28_19 | Bradi2g14880 | yes | 1.50E-81 | Glyco_hydro_28;  predicted active sites: D125, H148 | 9E-60, fragment match | ----- | ----- | min. | some |
|  | GH28_20 | Bradi2g17920 | 2.30E-56 | 1.10E-20 | Glyco_hydro_28 | 1.1E-13, fragment match | ----- | ----- | min. | some |
|  | GH28_21 | Bradi2g18030 | yes | 2.20E-66 | Glyco_hydro_28 | 4.8E-66, fragment match | ----- | ----- | yes | yes |
|  | GH28_22 | Bradi2g18040 | yes | 1.10E-80 | Glyco_hydro_28 | 2.8E-79 | ----- | ----- | yes | yes |
|  | GH28_23 | Bradi2g18050 | yes | 5.50E-86 | Glyco_hydro_28 | 5.2E-77 | ----- | ----- | yes | most |
|  | GH28_24 | Bradi2g18060 | yes | yes | Glyco_hydro_28;  predicted active sites: D268, H291 | 3.7E-116 | ----- | ----- | yes | yes |
|  | GH28_25 | Bradi2g33080 | yes | yes | Glyco_hydro_28;  predicted active sites: D245, H268 | 1.4E-135 | ----- | ----- | min. | no |
|  | GH28_26 | Bradi2g33440 | yes | yes | Glyco_hydro_28;  predicted active sites: D293, H316 | 8.6E-98, fragment match | ----- | ----- | yes | some |
|  | GH28_27 | Bradi2g43750 | yes | yes | Glyco_hydro_28 | 1.5E-29, fragment match | ----- | ----- | yes | yes |
|  | GH28_28 | Bradi2g44750 | yes | yes | Glyco_hydro_28;  predicted active sites: D351, H374 | 1.5E-92 | ----- | ----- | yes | yes |
|  | GH28_29 | Bradi2g46400 | yes | yes | Glyco_hydro_28;  predicted active sites: D219, H242 | 9.5E-136 | ----- | ----- | yes | some |
|  | GH28_30 | Bradi2g57430 | yes | 7.70E-95 | Glyco_hydro_28;  predicted active sites: D230, H253 | 1.4E-87 | ----- | ----- | yes | some |
|  | GH28_31 | Bradi3g02850 | yes | yes | Glyco_hydro_28;  predicted active sites: D309, H332 | 8.6E-112, fragment match | ----- | ----- | yes | some |
|  | GH28_32 | Bradi3g07120 | yes | 5.60E-84 | Glyco_hydro_28;  predicted active sites: D240, H263 | 1.4E-133 | ----- | ----- | yes | no |
|  | GH28_33 | Bradi3g13260 | yes | yes | Glyco_hydro_28;  Glyco_hydro_28 (2^nd^ of 2 such domains) | 2.1E-20, fragment match;  1.9E-08, fragment match | ----- | ----- | yes | most |
|  | GH28_34 | Bradi3g20540 | yes | 5.30E-96 | Glyco_hydro_28;  predicted active sites: D262, H285 | 3.3E-102 | ----- | ----- | min. | no |
|  | GH28_35 | Bradi3g42120 | yes | 3.60E-66 | Glyco_hydro_28;  predicted active sites: D215, H238 | 2.3E-87 | ----- | ----- | no | no |
|  | GH28_36 | Bradi3g56790 | yes | yes | Glyco_hydro_28 | 1.5E-28 | ----- | ----- | yes | most |
|  | GH28_37 | Bradi4g05050 | yes | yes | Glyco_hydro_28 | 1.6E-11, fragment match | ----- | ----- | yes | most |
|  | GH28_38 | Bradi4g11090 | yes | yes | Glyco_hydro_28 | 5E-14, fragment match | ----- | ----- | yes | most |
|  | GH28_39 | Bradi4g31250 | yes | yes | Glyco_hydro_28 | 2.4E-27 | ----- | ----- | yes | yes |
|  | GH28_40 | Bradi4g33660 | yes | yes | Glyco_hydro_28 | 8.2E-07 | ----- | ----- | yes | yes |
|  | GH28_41 | Bradi5g18370 | 1.60E-69 | 1.20E-67 | Glyco_hydro_28;  predicted active sites: D172, H195 | 2.1E-78, fragment match | ----- | ----- | min. | no |
|  |  |  |  |  |  |  |  |  |  |  |
| **29** | 2 |  |  |  |  |  |  |  |  |  |
|  | GH29_1 | Bradi4g35500 | yes | yes | *Alpha_L_fucos: Alpha-L-fucosidase*  Note: Bradi4g35500 and Bradi5g18220  were considered to be GHs because, although they lack a significant match to a Pfam GH domain, they are highly similar to the rice and Arabidopsis GH 29 family members Os09g0520800, Os04g0560400, and At2g28100, all of which have only insignificant matches to a predicted Pfam Alpha-L-fucosidase  domain. | *0.00029 (not significant!)* | ----- | ----- | yes | yes |
|  | GH29_2 | Bradi5g18220 | yes | yes | *Alpha-L-fucosidase* | *5.8E-05 (not significant!)* | ----- | ----- | yes | yes |
|  |  |  |  |  |  |  |  |  |  |  |
| **30** | --- |  |  |  |  |  |  |  |  |  |
|  |  |  |  |  |  |  |  |  |  |  |
| **31** | 6 |  |  |  |  |  |  |  |  |  |
|  | GH31_1 | Bradi1g32650 | **yes** | **yes** | Glyco_hydro_31: Glycosyl hydrolases family 31 | 4.50E-241 | ----- | ----- | no | no |
|  | GH31_2 | Bradi1g52510 | **yes** | **yes** | Glyco_hydro_31 | 8.70E-190 | ----- | ----- | yes | yes |
|  | GH31_3 | Bradi1g69780 | **yes** | **yes** | Glyco_hydro_31 | 2.90E-237 | ----- | ----- | yes | yes |
|  | GH31_4 | Bradi2g02070 | **yes** | **yes** | Glyco_hydro_31 | 3.60E-261 | ----- | ----- | yes | yes |
|  | GH31_5 | Bradi2g19280m | **yes** | **yes** | Glyco_hydro_31 | 1.50E-249 | ----- | ----- | yes | all but 1^st^ intron |
|  | GH31_6 | Bradi2g10060 | yes | 5.00E-20 | Glyco_hydro_31 | 4.30E-59 | ----- | ----- | no | no |
|  |  |  |  |  |  |  |  |  |  |  |
| **32** | 11 |  |  |  |  |  |  |  |  |  |
|  | GH32_1 | Bradi1g09500 | yes | yes | Glyco_hydro_32N:Glycosyl hydrolases family 32 N terminal; Glyco_hydro_32C: Glycosyl hydrolases family 32 C terminal | 1.7E-194;  2.3E-46 | ----- | ----- | yes | none |
|  | GH32_2 | Bradi1g52210 | yes | yes | Glyco_hydro_32N; Glyco_hydro_32C | 1.6E-207;  7.8E-46 | ----- | ----- | yes | yes |
|  | GH32_3 | Bradi2g61830 | yes | yes | Glyco_hydro_32N; Glyco_hydro_32C | 4.8E-183;  1E-41 | ----- | ----- | yes | yes |
|  | GH32_4 | Bradi3g00910 | yes | yes | Glyco_hydro_32N; Glyco_hydro_32C | 2E-209;  4.4E-48 | ----- | ----- | yes | yes |
|  | GH32_5 | Bradi3g44990 | yes | yes | Glyco_hydro_32N; Glyco_hydro_32C | 2.2E-191;  1.1E-51 | ----- | ----- | yes | yes |
|  | GH32_6 | Bradi3g46600 | yes | yes | Glyco_hydro_32N; Glyco_hydro_32C | 2.2E-167;  2.1E-37 | ----- | ----- | no | none |
|  | GH32_7 | Bradi4g07850 | yes | yes | Glyco_hydro_32N;  Glyco_hydro_32C | 9.1E-138, fragment match;  7.7E-36 | *other domains:*  tRNA-synt_2b: tRNA synthetase class II core domain (G, H, P, S and T);  HGTP_anti-codon: Anticodon binding domain;  ProRS-C_1: Prolyl-tRNA synthetase, C-terminal | 2.6E-15;  5.8E-29;  5.5E-27, fragment match | yes | yes |
|  | GH32_8 | Bradi5g09420 | yes | yes | Glyco_hydro_32N; Glyco_hydro_32C | 7.8E-198;  3.7E-50 | ----- | ----- | yes | yes |
|  | GH32_9 | Bradi5g09430 | yes | yes | Glyco_hydro_32N; Glyco_hydro_32C | 9.5E-204;  1.1E-47 | ----- | ----- | yes | min. |
|  | GH32_10 | Bradi5g16900 | yes | yes | Glyco_hydro_32N; Glyco_hydro_32C | 4.7E-209;  1.7E-45 | ----- | ----- | yes | yes |
|  | GH32_11 | Bradi5g25270 | yes | yes | Glyco_hydro_32N; Glyco_hydro_32C | 5.3E-170;  6.9E-52 | ----- | ----- | yes | yes |
|  |  |  |  |  |  |  |  |  |  |  |
| **33** | 1 |  |  |  |  |  |  |  |  |  |
|  | GH33_1 | Bradi1g26290m | yes | yes | *-----*  Neither the rice (Os07g0516000) nor the Arabidopsis (At5g57700) GH33 family members have any significant matches to Pfam domains. The Pfam database does not contain a specific entry for a GH33 domain. | *-----* | ----- | ----- | yes | yes |
|  |  |  |  |  |  |  |  |  |  |  |
| **34** | --- |  |  |  |  |  |  |  |  |  |
|  |  |  |  |  |  |  |  |  |  |  |
| **35** | 15 |  |  |  |  |  |  |  |  |  |
|  | GH35_1 | Bradi1g37450 | **yes** | yes | Glyco_hydro_35: Glycosyl hydrolases family 35 | 1.9E-184, fragment match | ----- | ----- | yes | yes |
|  | GH35_2 | Bradi1g67760 | **yes** | **yes** | Glyco_hydro_35 | 1.2E-189, fragment match | [Gal_Lectin](http://pfam.sanger.ac.uk/family?acc=PF02140): Galactose binding lectin domain | 3.1E-34 | yes | yes |
|  | GH35_3 | Bradi1g74050 | **yes** | **yes** | Glyco_hydro_35 | 7.8E-192, fragment match | [Gal_Lectin](http://pfam.sanger.ac.uk/family?acc=PF02140) | 7.3E-41 | yes | most |
|  | GH35_4 | Bradi2g18440 | yes | yes | Glyco_hydro_35 | 1.5E-160, fragment match | ----- | ----- | yes | most |
|  | GH35_5 | Bradi2g24670 | yes | yes | Glyco_hydro_35 | 1.9E-104, fragment match | [Gal_Lectin](http://pfam.sanger.ac.uk/family?acc=PF02140) | 1E-30 | yes | some |
|  | GH35_6 | Bradi2g39450 | yes | yes | Glyco_hydro_35;  Glyco_hydro_35 (2^nd^ of 2 adjacent domain fragments) | 1.5E-35, fragment match;  1.1E-35, fragment match | [Gal_Lectin](http://pfam.sanger.ac.uk/family?acc=PF02140) | 1.5E-08 | min. | some |
|  | GH35_7 | Bradi2g40450 | **yes** | yes | Glyco_hydro_35 | 4.4E-185, fragment match | [Gal_Lectin](http://pfam.sanger.ac.uk/family?acc=PF02140) | 7.7E-13 | min. | some |
|  | GH35_8 | Bradi2g41830 | **yes** | **yes** | Glyco_hydro_35 | 2E-183, fragment match | [Gal_Lectin](http://pfam.sanger.ac.uk/family?acc=PF02140) | 9.1E-38 | yes | most |
|  | GH35_9 | Bradi2g56600 | yes | 3.00E-98 | Glyco_hydro_35;  Glyco_hydro_35 (2^nd^ of 2 adjacent domain fragments) | 4.7E-67, fragment match; 4.4E-09, fragment match | ----- | ----- | min. | min. |
|  | GH35_10 | Bradi2g56610 | **yes** | yes | Glyco_hydro_35 | 8.1E-164, fragment match | [Gal_Lectin](http://pfam.sanger.ac.uk/family?acc=PF02140) | 4.5E-33 | yes | most |
|  | GH35_11 | Bradi3g08180 | **yes** | yes | Glyco_hydro_35 | 5.6E-188, fragment match | ----- | ----- | yes | most |
|  | GH35_12 | Bradi3g42330 | **yes** | yes | Glyco_hydro_35 | 5.1E-156, fragment match | [Gal_Lectin](http://pfam.sanger.ac.uk/family?acc=PF02140) | 4.4E-30 | no | min. |
|  | GH35_13 | Bradi3g49460 | **yes** | yes | Glyco_hydro_35 | 3.2E-181, fragment match | [Gal_Lectin](http://pfam.sanger.ac.uk/family?acc=PF02140) | 1.8E-10 | min. | min. |
|  | GH35_14 | Bradi4g07990 | **yes** | **yes** | Glyco_hydro_35 | 2.1E-169, fragment match | [Gal_Lectin](http://pfam.sanger.ac.uk/family?acc=PF02140) | 1.2E-36 | yes | most |
|  | GH35_15 | Bradi4g36520 | yes | yes | Glyco_hydro_35 | 6.1E-155, fragment match | ----- | ----- | yes | most |
|  |  |  |  |  |  |  |  |  |  |  |
| **36** | 6 |  |  |  |  |  |  |  |  |  |
|  | GH36_1 | Bradi1g44930 | yes | yes | Raffinose_syn: Raffinose synthase | 0 | ----- | ----- | none | none |
|  | GH36_2 | Bradi1g48050 | yes | yes | Raffinose_syn | 0 | ----- | ----- | yes | yes |
|  | GH36_3 | Bradi1g54210 | **yes** | yes | Raffinose_syn | 0 | ----- | ----- | yes | yes |
|  | GH36_4 | Bradi2g04310 | **yes** | yes | Raffinose_syn | 0 | ----- | ----- | yes | yes |
|  | GH36_5 | Bradi3g39220 | **yes** | yes | Raffinose_syn | 0 | ----- | ----- | yes | yes |
|  | GH36_6 | Bradi5g13570 | **yes** | yes | Raffinose_syn | 9.50E-296 | ----- | ----- | yes | yes |
|  |  |  |  |  |  |  |  |  |  |  |
| **37** | 1 |  |  |  |  |  |  |  |  |  |
|  | GH37_1 | Bradi3g31410 | yes | yes | Trehalase | 4.90E-263 | ----- | ----- | yes | yes |
|  |  |  |  |  |  |  |  |  |  |  |
| **38** | 5 |  |  |  |  |  |  |  |  |  |
|  | GH38_1 | Bradi1g44550 | **yes** | **yes** | Glyco_hydro_38: Glycosyl hydrolases family 38 N-terminal domain;  Alpha-mann_mid: Alpha mannosidase, middle domain;  Glyco_hydro_38C: Glycosyl hydrolases family 38 C-terminal domain | 7.00E-151  2.30E-33  2.90E-97 | ----- | ----- | yes | yes |
|  | GH38_2 | Bradi3g21120 | yes | yes | Alpha-mann_mid;  Glyco_hydro_38C | 5.20E-07, fragment match;  7.40E-132 | ----- | ----- | most | most |
|  | GH38_3 | Bradi4g17310m | yes | yes | Glyco_hydro_38;  Alpha-mann_mid;  Glyco_hydro_38C | 5.9E-79;  3.2E-20, fragment match;  5.4E-79,  fragment match | ----- | ----- | min. | no |
|  | GH38_4 | Bradi4g17320m | yes | yes | Glyco_hydro_38;  Alpha-mann_mid;  Glyco_hydro_38C | 5.3E-84;  1.8E-20, fragment match;  5.4E-94,  fragment match | ----- | ----- | min. | no |
|  | GH38_5 | Bradi4g17330m | yes | yes | Glyco_hydro_38;  Alpha-mann_mid;  Glyco_hydro_38C | 8E-83;  1.3E-20, fragment match;  6.7E-94,  fragment match | ----- | ----- | min. | no |
|  |  |  |  |  |  |  |  |  |  |  |
| **39** | --- |  |  |  |  |  |  |  |  |  |
| **(40)** | --- |  |  |  |  |  |  |  |  |  |
| **(41)** | --- |  |  |  |  |  |  |  |  |  |
| **42** | --- |  |  |  |  |  |  |  |  |  |
|  |  |  |  |  |  |  |  |  |  |  |
| **43** | 2 |  |  |  |  |  |  |  |  |  |
|  | GH43_1 | Bradi1g71160 | yes | yes | Glyco_hydro_43: Glycosyl hydrolases family 43 | 1.1E-11, fragment match | ----- | ----- | yes | yes |
|  | GH43_2 | Bradi2g50980 | yes | yes | Glyco_hydro_43 | 4.9E-07, fragment match | ----- | ----- | yes | yes |
|  |  |  |  |  |  |  |  |  |  |  |
| **44** | --- |  |  |  |  |  |  |  |  |  |
| **45** | --- |  |  |  |  |  |  |  |  |  |
| **46** | --- |  |  |  |  |  |  |  |  |  |
|  |  |  |  |  |  |  |  |  |  |  |
| **47** | 4 |  |  |  |  |  |  |  |  |  |
|  | GH47_1 | Bradi2g32570 | **yes** | yes | Glyco_hydro_47: Glycosyl hydrolase family 47 | 2.9E-179 | ----- | ----- | yes | yes |
|  | GH47_2 | Bradi2g51470 | yes | yes | Glyco_hydro_47 | 2.8E-183 | ----- | ----- | yes | most |
|  | GH47_3 | Bradi3g59640 | yes | yes | Glyco_hydro_47 | 2.4E-156 | ----- | ----- | yes | yes |
|  | GH47_4 | Bradi5g20920 | yes | yes | Glyco_hydro_47 | 5.4E-195 | ----- | ----- | yes | yes |
|  |  |  |  |  |  |  |  |  |  |  |
| **48** | --- |  |  |  |  |  |  |  |  |  |
| **49** | --- |  |  |  |  |  |  |  |  |  |
| **50** | --- |  |  |  |  |  |  |  |  |  |
|  |  |  |  |  |  |  |  |  |  |  |
| **51** | 5 |  |  |  |  |  |  |  |  |  |
|  | GH51_1 | Bradi1g17260 | **yes** | **yes** | Alpha-L-AF_C: Alpha-L-arabinofuranosidase C-terminus | 1.3E-61 | ----- | ----- | yes | most |
|  | GH51_2 | Bradi1g57020 | **yes** | yes | Alpha-L-AF_C | 4.2E-57 | ----- | ----- | yes | most |
|  | GH51_3 | Bradi1g63990 | **yes** | **yes** | Alpha-L-AF_C | 4.9E-65 | CBM_4_9: carbohydrate binding domain | 6.6e-05 | yes | most |
|  | GH51_4 | Bradi4g26270 | **yes** | **yes** | Alpha-L-AF_C | 4.8E-68 | CBM_4_9 | 0.0001 | yes | most |
|  | GH51_5 | Bradi4g43710 | **yes** | **yes** | Alpha-L-AF_C | 2.8E-67 | CBM_4_9 | 0.00013 | yes | yes |
|  |  |  |  |  |  |  |  |  |  |  |
| **52** | --- |  |  |  |  |  |  |  |  |  |
| **53** | --- |  |  |  |  |  |  |  |  |  |
| **54** | --- |  |  |  |  |  |  |  |  |  |
| **55** | --- |  |  |  |  |  |  |  |  |  |
| **56** | --- |  |  |  |  |  |  |  |  |  |
| **57** | --- |  |  |  |  |  |  |  |  |  |
| **58** | --- |  |  |  |  |  |  |  |  |  |
| **59** | --- |  |  |  |  |  |  |  |  |  |
| **(60)** | --- |  |  |  |  |  |  |  |  |  |
| **61** | --- |  |  |  |  |  |  |  |  |  |
| **62** | --- |  |  |  |  |  |  |  |  |  |
|  |  |  |  |  |  |  |  |  |  |  |
| **63** | 1 |  |  |  |  |  |  |  |  |  |
|  | GH63_1 | Bradi2g59040 | **yes** | yes | Glyco_hydro_63: Mannosyl oligosaccharide glucosidase | 0 | ----- | ----- | yes | yes |
|  |  |  |  |  |  |  |  |  |  |  |
| **64** | --- |  |  |  |  |  |  |  |  |  |
| **65** | --- |  |  |  |  |  |  |  |  |  |
| **66** | --- |  |  |  |  |  |  |  |  |  |
| **67** | --- |  |  |  |  |  |  |  |  |  |
| **68** | --- |  |  |  |  |  |  |  |  |  |
| **(69)** | --- |  |  |  |  |  |  |  |  |  |
| **70** | --- |  |  |  |  |  |  |  |  |  |
| **71** | --- |  |  |  |  |  |  |  |  |  |
| **72** | --- |  |  |  |  |  |  |  |  |  |
| **73** | --- |  |  |  |  |  |  |  |  |  |
| **74** | --- |  |  |  |  |  |  |  |  |  |
| **75** | --- |  |  |  |  |  |  |  |  |  |
| **76** | --- |  |  |  |  |  |  |  |  |  |
|  |  |  |  |  |  |  |  |  |  |  |
| **77** | 2 |  |  |  |  |  |  |  |  |  |
|  | GH77_1 | Bradi1g18650 | **yes** | **yes** | Glyco_hydro_77: 4-alpha-glucanotransferase | 1.6E-178 | CBM_20: Starch binding domain;  CBM_20  (2^nd^ of 2 sequential CBMs) | 9.5e-24, fragment match;  7e-06 | yes | yes |
|  | GH77_2 | Bradi1g20790 | yes | yes | Glyco_hydro_77 | 6.1E-213 | ----- | ----- | yes | most |
|  |  |  |  |  |  |  |  |  |  |  |
| **78** | --- |  |  |  |  |  |  |  |  |  |
|  |  |  |  |  |  |  |  |  |  |  |
| **79** | 5 |  |  |  |  |  |  |  |  |  |
|  | GH79_1 | Bradi1g22580 | yes | yes | Glyco_hydro_79n: Glycosyl hydrolase family 79, N-terminal domain | 6.10E-22, fragment match | ----- | ----- | none | none |
|  | GH79_2 | Bradi1g47760 | yes | yes | Glyco_hydro_79n | 2.50E-160 | ----- | ----- | yes | yes |
|  | GH79_3 | Bradi1g70300 | yes | yes | Glyco_hydro_79n | 2.40E-157 | ----- | ----- | yes | yes |
|  | GH79_4 | Bradi3g54370 | yes | yes | Glyco_hydro_79n | 2.40E-162 | ----- | ----- | yes | yes |
|  | GH79_5 | Bradi4g03680 | yes | yes | Glyco_hydro_79n | 1.40E-169 | ----- | ----- | yes | yes |
|  |  |  |  |  |  |  |  |  |  |  |
| **80** | --- |  |  |  |  |  |  |  |  |  |
|  |  |  |  |  |  |  |  |  |  |  |
| **81** | 1 |  |  |  |  |  |  |  |  |  |
|  | GH81_1 | Bradi4g29390 | yes | yes | Glyco_hydro_81: Glycosyl hydrolase family 81 | 4.30E-250 | ----- | ----- | yes | none predicted |
|  |  |  |  |  |  |  |  |  |  |  |
| **82** | --- |  |  |  |  |  |  |  |  |  |
| **83** | --- |  |  |  |  |  |  |  |  |  |
| **84** | --- |  |  |  |  |  |  |  |  |  |
|  |  |  |  |  |  |  |  |  |  |  |
| **85** | 1 |  |  |  |  |  |  |  |  |  |
|  | GH85_1 | Bradi1g14130 | yes | yes | Glyco_hydro_85: Glycosyl hydrolase family 85 | 2.1E-135 | ----- | ----- | yes | yes |
|  |  |  |  |  |  |  |  |  |  |  |
| **86** | --- |  |  |  |  |  |  |  |  |  |
| **87** | --- |  |  |  |  |  |  |  |  |  |
| **88** | --- |  |  |  |  |  |  |  |  |  |
|  |  |  |  |  |  |  |  |  |  |  |
| **89** | 2 |  |  |  |  |  |  |  |  |  |
|  | GH89_1 | Bradi1g62010 | yes | yes | NAGLU: Alpha-N-acetylglucosamini-dase | 0 | ----- | ----- | yes | yes |
|  | GH89_2 | Bradi5g24210 | yes | yes | NAGLU | 3.2E-276 | ----- | ----- | no | yes |
|  |  |  |  |  |  |  |  |  |  |  |
| **90** | --- |  |  |  |  |  |  |  |  |  |
| **(91)** | --- |  |  |  |  |  |  |  |  |  |
| **92** | --- |  |  |  |  |  |  |  |  |  |
| **93** | --- |  |  |  |  |  |  |  |  |  |
| **94** | --- |  |  |  |  |  |  |  |  |  |
|  |  |  |  |  |  |  |  |  |  |  |
| **95** | 2 |  |  |  |  |  |  |  |  |  |
|  | GH95_1 | Bradi1g28370 | yes | yes | *Glycosyl hydrolase family 65 central catalytic domain*;  Note: Bradi1g28370 and Bradi3g25160 were considered to be GHs because, although they lack a significant match to a Pfam GH domain, they are highly similar to the rice and Arabidopsis GH95 family members Os10g0339600 and At4g34260, neither of which has a predicted Pfam GH domain. The Pfam database does not contain a specific entry for a GH95 domain. | *0.3 (not significant!), fragment match* | ----- | ----- | yes | most |
|  | GH95_2 | Bradi3g25160 | **yes** | yes | ----- | ----- | ----- | ----- | yes | yes |
|  |  |  |  |  |  |  |  |  |  |  |
| **96** | --- |  |  |  |  |  |  |  |  |  |
| **97** | --- |  |  |  |  |  |  |  |  |  |
| **98** | --- |  |  |  |  |  |  |  |  |  |
| **99** | --- |  |  |  |  |  |  |  |  |  |
|  |  |  |  |  |  |  |  |  |  |  |
| **100** | 8 |  |  |  |  |  |  |  |  |  |
|  | GH100_1 | Bradi1g64150 | yes | yes | Invertase_neut: Plant neutral invertase | 0 | ----- | ----- | yes | yes |
|  | GH100_2 | Bradi2g12430 | yes | yes | Invertase_neut | 0 | ----- | ----- | yes | yes |
|  | GH100_3 | Bradi3g02560 | yes | yes | Invertase_neut | 4.5E-290 | ----- | ----- | min. | no |
|  | GH100_4 | Bradi3g44770 | yes | yes | Invertase_neut | 0 | ----- | ----- | yes | yes |
|  | GH100_5 | Bradi3g45530 | yes | yes | Invertase_neut | 0 | ----- | ----- | yes | yes |
|  | GH100_6 | Bradi4g24470 | yes | yes | Invertase_neut | 0 | ----- | ----- | yes | yes |
|  | GH100_7 | Bradi5g09200 | yes | yes | Invertase_neut | 0 | ----- | ----- | yes | yes |
|  | GH100_8 | Bradi5g10360 | yes | yes | Invertase_neut | 0 | ----- | ----- | yes | yes |
|  |  |  |  |  |  |  |  |  |  |  |
| **101** | --- |  |  |  |  |  |  |  |  |  |
| **102** | --- |  |  |  |  |  |  |  |  |  |
| **103** | --- |  |  |  |  |  |  |  |  |  |
| **104** | --- |  |  |  |  |  |  |  |  |  |
| **105** | --- |  |  |  |  |  |  |  |  |  |
| **106** | --- |  |  |  |  |  |  |  |  |  |
| **107** | --- |  |  |  |  |  |  |  |  |  |
| **108** | --- |  |  |  |  |  |  |  |  |  |
| **109** | --- |  |  |  |  |  |  |  |  |  |
| **110** | --- |  |  |  |  |  |  |  |  |  |
| **111** | --- |  |  |  |  |  |  |  |  |  |
| **112** | --- |  |  |  |  |  |  |  |  |  |
| **113** | --- |  |  |  |  |  |  |  |  |  |
| **114** | --- |  |  |  |  |  |  |  |  |  |
| **115** | --- |  |  |  |  |  |  |  |  |  |

**^a^** with an *E*-value of 10E-100 or less; If there is a match, but the threshold of *E* = 10E-100 is not met, the best *E*-value is given. Bold type indicates a match with *E*-value = 0.0 .

**^b^** Matches are for full-length domain models, unless otherwise indicated.

**^c^** A “yes” in this column means there is Brachypodium EST and/or Illumina transcriptome support for the corresponding gene model and its expression. The note “min.” indicates there is minimal support.

**^d^** Splice junctions were checked against the HTS SuperSplat Splice Junctions information available at www.brachybase.org .

**^e^** The designation “m” indicates that the gene model has been modified relative to the v1.0 annotation.

**^f^** Parentheses indicate that the GH family has been deleted from the CAZy database (www.cazy.org) .
