# Supplementary material for: Annotation and comparative analysis of the glycoside hydrolase genes in Brachypodium distachyon
Source: BMC Genomics. 2010 Oct 25;11:600. doi: 10.1186/1471-2164-11-600 (PMC3091745; doi:10.1186/1471-2164-11-600)
Supplement: Additional file 10 — GH18 Rectangular Tree. GH18 Rectangular Tree This figure presents the same phylogenetic tree as Figure 3, but in a rectangular format, with complete bootstrap information and branch labels. The tree includes GH18 proteins from Arabidopsis, poplar, rice, Brachypodium, sorghum, and 18 other plants. [file 1471-2164-11-600-S10.PDF]

GH18

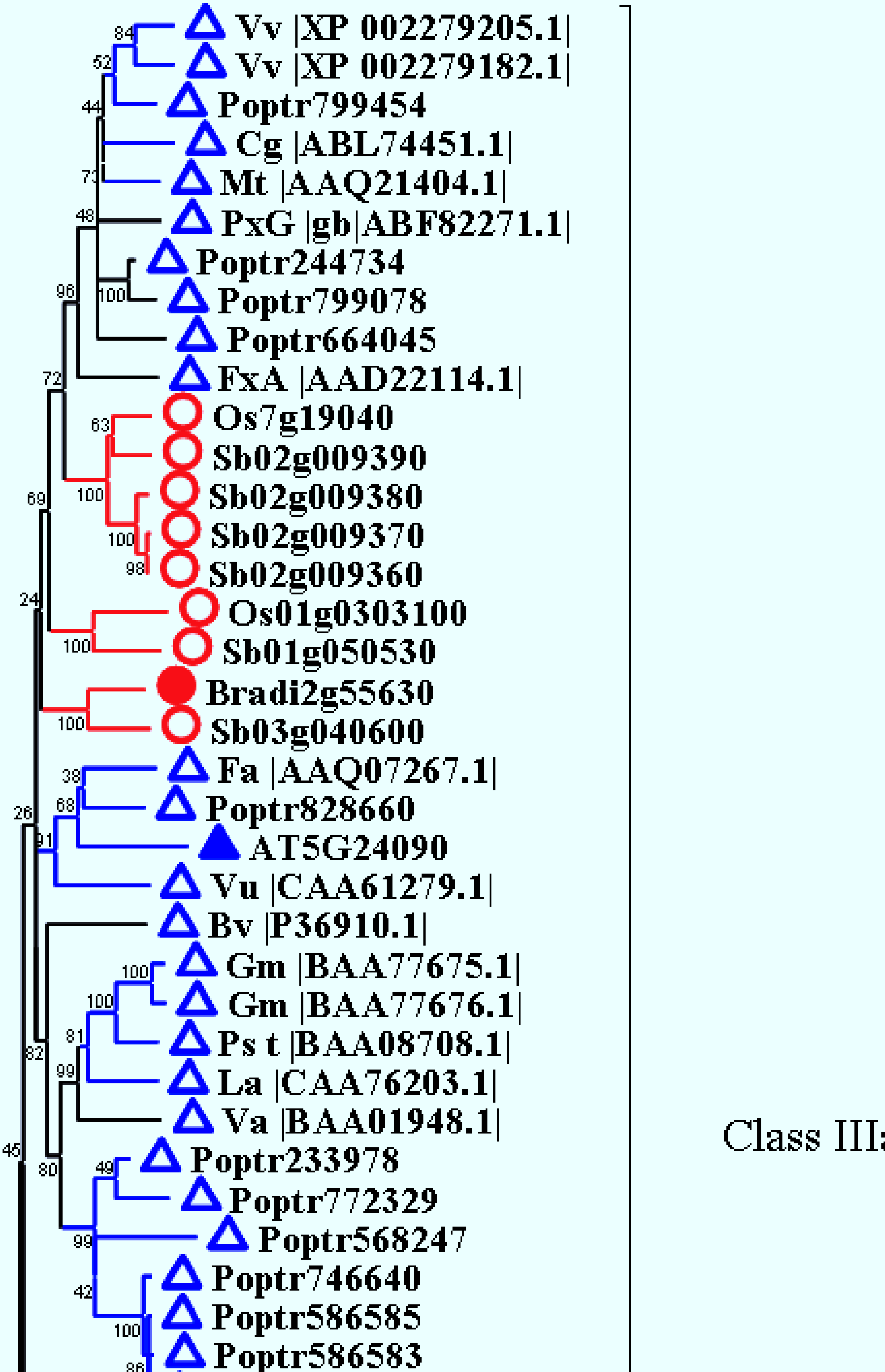

Vv |XP 002279205.1|
Vv |XP 002279182.1|
Poptr799454
Cg |ABL74451.1|
Mt |AAQ21404.1|
PxG |gb|ABF82271.1|
Poptr244734
Poptr799078
Poptr664045
FxA |AAD22114.1|
Os7g19040
Sb02g009390
Sb02g009380
Sb02g009370
Sb02g009360
Os01g0303100
Sb01g050530
Bradi2g55630
Sb03g040600
Fa |AAQ07267.1|
Poptr828660
AT5G24090
Vu |CAA61279.1|
Bv |P36910.1|
Gm |BAA77675.1|
Gm |BAA77676.1|
Ps t |BAA08708.1|
La |CAA76203.1|
Va |BAA01948.1|
Poptr233978
Poptr772329
Poptr568247
Poptr746640
Poptr586585
Poptr586583
Class III

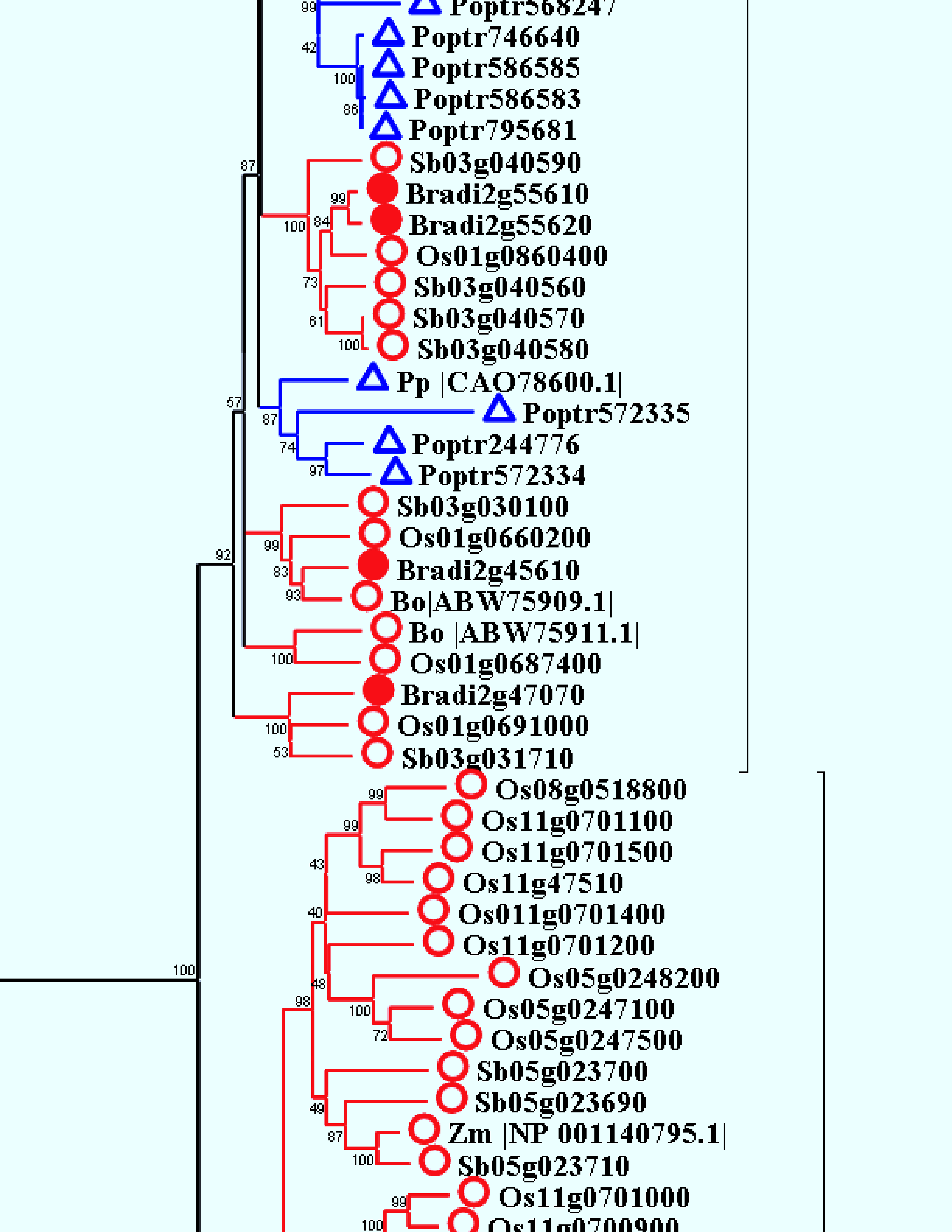
Poptr746640
Poptr586585
Poptr586583
Poptr795681
Sb03g040590
Bradi2g55610
Bradi2g55620
Os01g0860400
Sb03g040560
Sb03g040570
Sb03g040580
Pp |CAO78600.1|
Poptr572335
Poptr244776
Poptr572334
Sb03g030100
Os01g0660200
Bradi2g45610
Bo|ABW75909.1|
Bo |ABW75911.1|
Os01g0687400
Bradi2g47070
Os01g0691000
Sb03g031710
Os08g0518800
Os11g0701100
Os11g0701500
Os11g47510
Os011g0701400
Os11g0701200
Os05g0248200
Os05g0247100
Os05g0247500
Sb05g023700
Sb05g023690
Zm |NP 001140795.1|
Sb05g023710
Os11g0701000

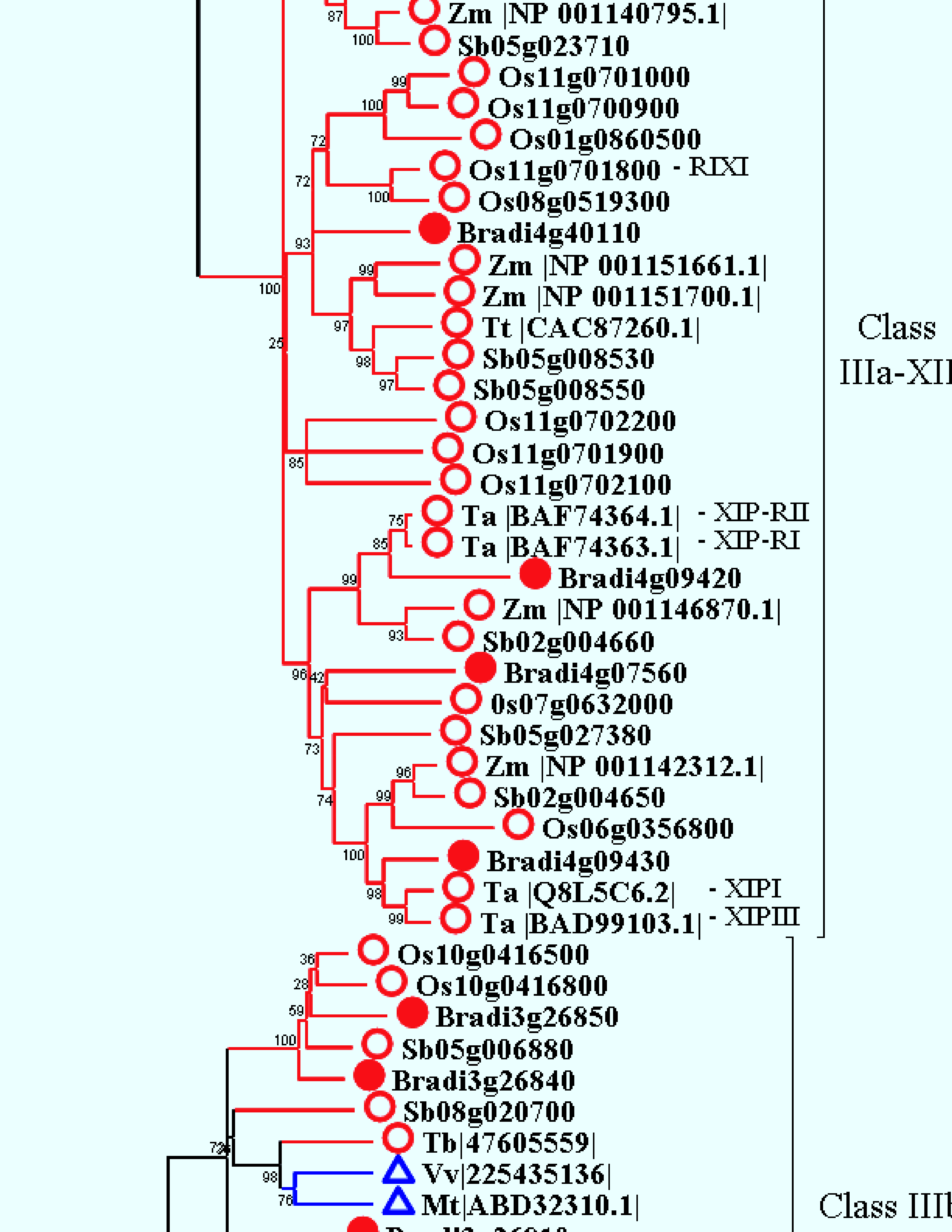
Zm |NP 001140795.1|
Sb05g023710
Os11g0701000
Os11g0700900
Os01g0860500
Os11g0701800 - RIXI
Os08g0519300
Bradi4g40110
Zm |NP 001151661.1|
Zm |NP 001151700.1|
Tt |CAC87260.1|
Sb05g008530
Sb05g008550
Os11g0702200
Os11g0701900
Os11g0702100
Ta |BAF74364.1| - XIP-RII
Ta |BAF74363.1| - XIP-RI
Bradi4g09420
Zm |NP 001146870.1|
Sb02g004660
Bradi4g07560
0s07g0632000
Sb05g027380
Zm |NP 001142312.1|
Sb02g004650
Os06g0356800
Bradi4g09430
Ta |Q8L5C6.2| - XIPI
Ta |BAD99103.1| - XIPIII
Os10g0416500
Os10g0416800
Bradi3g26850
Sb05g006880
Bradi3g26840
Sb08g020700
Tb|47605559|
Vv|225435136|
Mt|ABD32310.1|
Class
IIIa-XII
Class IIIb

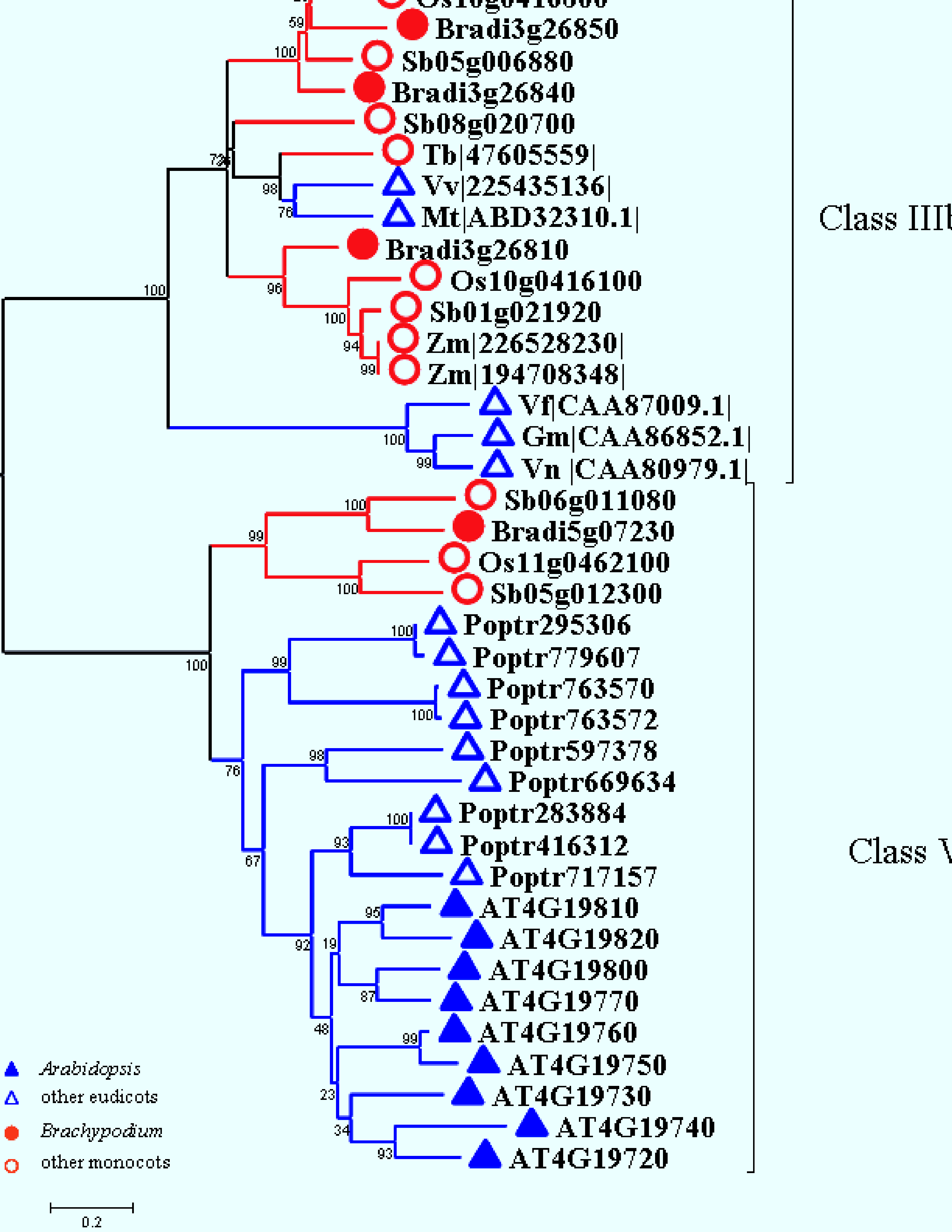

Bradi3g26850
Sb05g006880
Bradi3g26840
Sb08g020700
Tb|47605559|
Vv|225435136|
Mt|ABD32310.1|
Bradi3g26810
Os10g0416100
Sb01g021920
Zm|226528230|
Zm|194708348|
Vf|CAA87009.1|
Gm|CAA86852.1|
Vn |CAA80979.1|
Sb06g011080
Bradi5g07230
Os11g0462100
Sb05g012300
Poptr295306
Poptr779607
Poptr763570
Poptr763572
Poptr597378
Poptr669634
Poptr283884
Poptr416312
Poptr717157
AT4G19810
AT4G19820
AT4G19800
AT4G19770
AT4G19760
AT4G19750
AT4G19730
AT4G19740
AT4G19720
Class IIIb
Class V
Arabidopsis
other eudicots
Brachypodium
other monocots
0.2

Additional file 10 – GH18 Rectangular Tree. GH18 family tree with all branches labeled. The same tree as in Figure 2 is displayed in a rectangular format, with complete branch information. The tree is comprised of GH18 proteins from 23 species, including *Arabidopsis* (AT), rice (Os), *Brachypodium* (Bradi), sorghum (Sb), and poplar (Poptr). Additional plant sequences are indicated by an abbreviation for the genus and species, followed by the GenBank accession number or gene name. The tree was constructed using the Neighbor-Joining method and 1,000 bootstrap replicates. The bootstrap support for each branch is indicated. Distances represent the number of amino-acid substitutions per site. Sequences from eudicots are indicated in blue (*Arabidopsis* with filled triangles, other eudicots with open triangles); sequences from monocots are indicated in red (*Brachypodium* with filled circles, other monocots with open circles). Chitinase classes and specific XIP proteins are labeled on the right. Bo: *Bambusa oldhamii,* Bv: *Beta vulgaris*, Cg: *Casuarina glauca,* Fa: *Ficus awkeotsang*, FxA: *Fragaria x ananassa*, Gm: *Glycine max*, La: *Lupinus albus,* Mt: *Medicago truncatula*, Pp: *Parkia platycephala*, Pst: *Psophocarpus tetragonolobus*, PxG: *Panax ginseng*, Ta: *Triticum aestivum,* Tb: *Tulipa bakeri,* Tt: *Triticum turgidum durum*, Va: *Vigna angularis*, Vu: *Vigna unguiculata*, Vv: *Vitis vinifera*, Zm: *Zea mays*, Common names for these species can be found in additional file 12. Poplar gene names are abbreviated; for the full names see additional file 9.
