# Supplementary material for: Annotation and comparative analysis of the glycoside hydrolase genes in Brachypodium distachyon
Source: BMC Genomics. 2010 Oct 25;11:600. doi: 10.1186/1471-2164-11-600 (PMC3091745; doi:10.1186/1471-2164-11-600)
Supplement: Additional file 12 — GH19 Rectangular Tree. GH19 Rectangular Tree This figure presents the same phylogenetic tree as Figure 4, but in a rectangular format, with complete bootstrap information and branch labels. The tree includes GH19 proteins from Arabidopsis, poplar, rice, Brachypodium, and sorghum. [file 1471-2164-11-600-S12.PDF]

# GH19

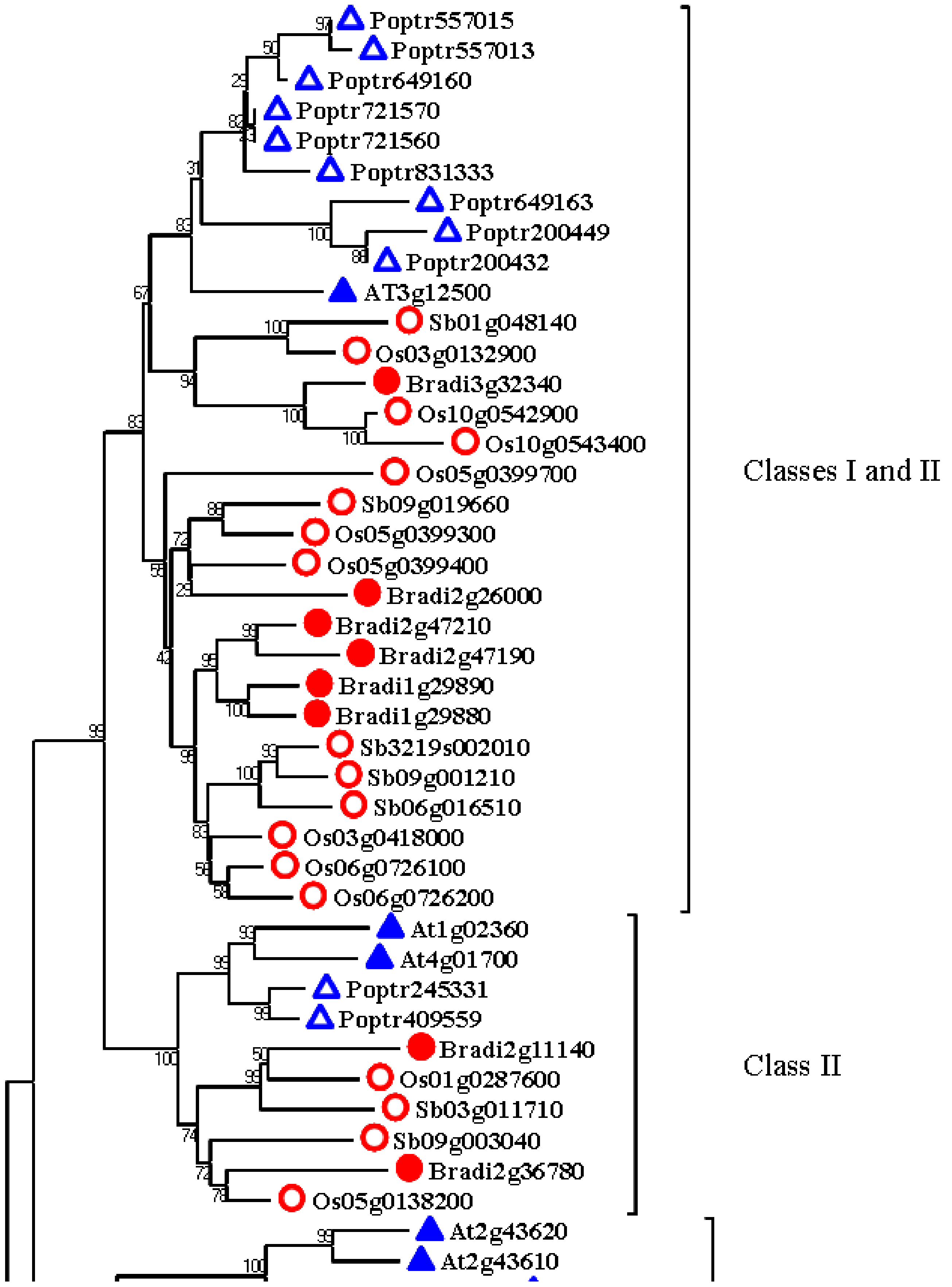

Poptr557015
Poptr557013
Poptr649160
Poptr721570
Poptr721560
Poptr831333
Poptr649163
Poptr200449
Poptr200432
AT3g12500
Sb01g048140
Os03g0132900
Bradi3g32340
Os10g0542900
Os10g0543400
Os05g0399700
Sb09g019660
Os05g0399300
Os05g0399400
Bradi2g26000
Bradi2g47210
Bradi2g47190
Bradi1g29890
Bradi1g29880
Sb3219s002010
Sb09g001210
Sb06g016510
Os03g0418000
Os06g0726100
Os06g0726200
At1g02360
At4g01700
Poptr245331
Poptr409559
Bradi2g11140
Os01g0287600
Sb03g011710
Sb09g003040
Bradi2g36780
Os05g0138200
At2g43620
At2g43610
Classes I and II
Class II

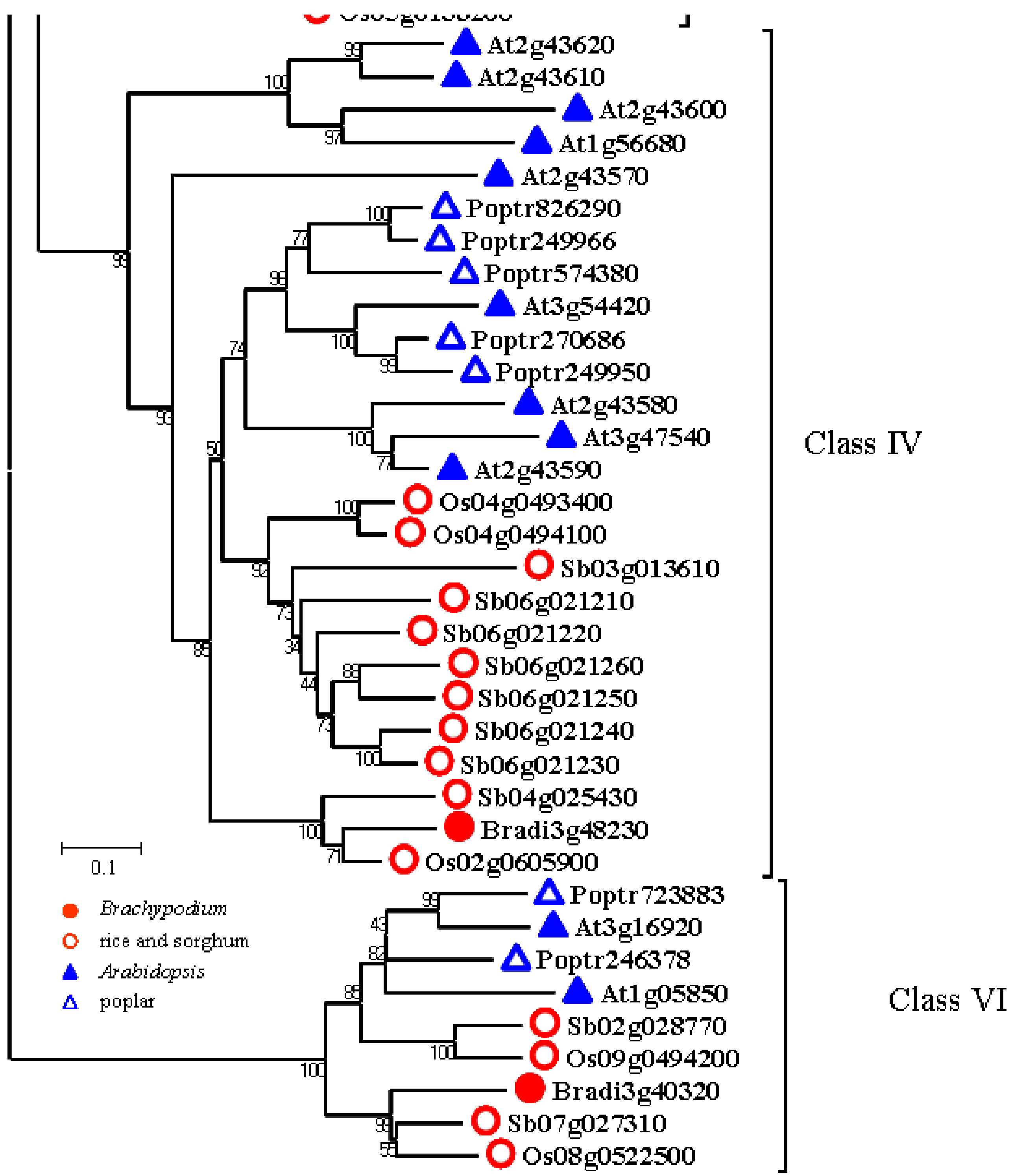

At2g43620
At2g43610
At2g43600
At1g56680
At2g43570
Poptr826290
Poptr249966
Poptr574380
At3g54420
Poptr270686
Poptr249950
At2g43580
At3g47540
At2g43590
Os04g0493400
Os04g0494100
Sb03g013610
Sb06g021210
Sb06g021220
Sb06g021260
Sb06g021250
Sb06g021240
Sb06g021230
Sb04g025430
Bradi3g48230
Os02g0605900
Poptr723883
At3g16920
Poptr246378
At1g05850
Sb02g028770
Os09g0494200
Bradi3g40320
Sb07g027310
Os08g0522500
Class IV
Class VI
0.1
Brachypodium
rice and sorghum
Arabidopsis
poplar

**Additional file 12 – GH19 Rectangular Tree. GH19 family tree with all branches labeled.** The same tree as in Figure 4 is displayed in a rectangular format, with complete branch information. The tree is comprised of GH19 proteins from 5 species, *Arabidopsis* (AT), rice (Os), *Brachypodium* (Bradi), sorghum (Sb), and poplar (Poptr). The tree was constructed using the Neighbor-Joining method and 1,000 bootstrap replicates. The bootstrap support for each branch is indicated. Distances represent the number of amino-acid substitutions per site. Sequences from eudicots are indicated in blue (*Arabidopsis* with filled triangles, poplar with open triangles); sequences from grasses are indicated in red (*Brachypodium* with filled circles, rice and sorghum with open circles). Poplar gene names are abbreviated; for the full names see additional file 9. Chitinase classes are labeled on the right.
