# Supplementary material for: Annotation and comparative analysis of the glycoside hydrolase genes in Brachypodium distachyon
Source: BMC Genomics. 2010 Oct 25;11:600. doi: 10.1186/1471-2164-11-600 (PMC3091745; doi:10.1186/1471-2164-11-600)
Supplement: Additional file 13 — GH5 Rectangular Tree. GH5 Rectangular Tree This figure presents the same phylogenetic tree as Figure 5, but in a rectangular format, with complete bootstrap information and branch labels. The tree includes GH5 proteins from Arabidopsis, poplar, rice, Brachypodium, sorghum, and 23 other species. [file 1471-2164-11-600-S13.PDF]

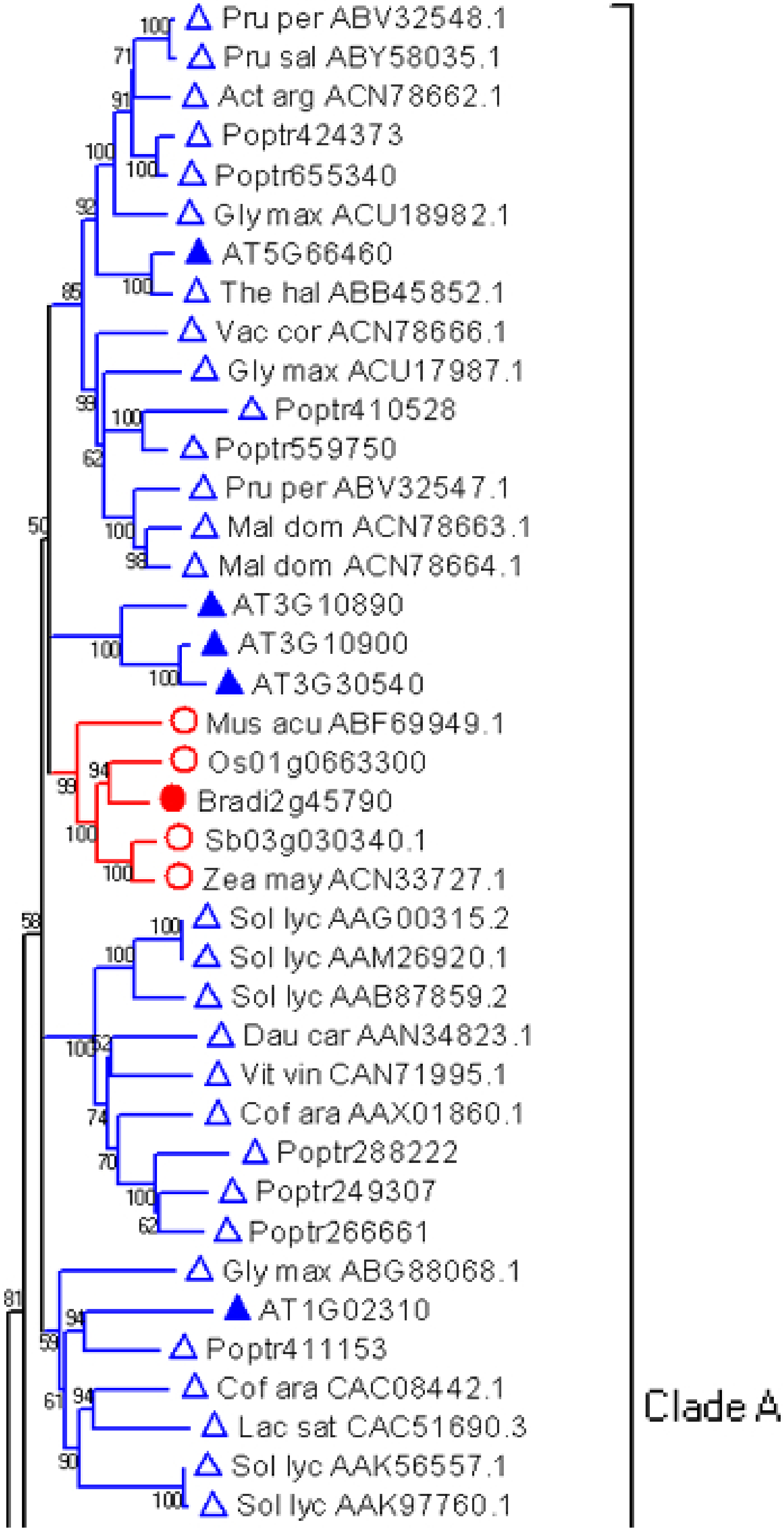

Pru per ABV32548.1
Pru sal ABY58035.1
Act arg ACN78662.1
Poptr424373
Poptr655340
Gly max ACU18982.1
AT5G66460
The hal ABB45852.1
Vac cor ACN78666.1
Gly max ACU17987.1
Poptr410528
Poptr559750
Pru per ABV32547.1
Mal dom ACN78663.1
Mal dom ACN78664.1
AT3G10890
AT3G10900
AT3G30540
Mus acu ABF69949.1
Os01g0663300
Bradi2g45790
Sb03g030340.1
Zea may ACN33727.1
Sol lyc AAG00315.2
Sol lyc AAM26920.1
Sol lyc AAB87859.2
Dau car AAN34823.1
Vit vin CAN71995.1
Cof ara AAX01860.1
Poptr288222
Poptr249307
Poptr266661
Gly max ABG88068.1
AT1G02310
Poptr411153
Cof ara CAC08442.1
Lac sat CAC51690.3
Sol lyc AAK56557.1
Sol lyc AAK97760.1
Clade A

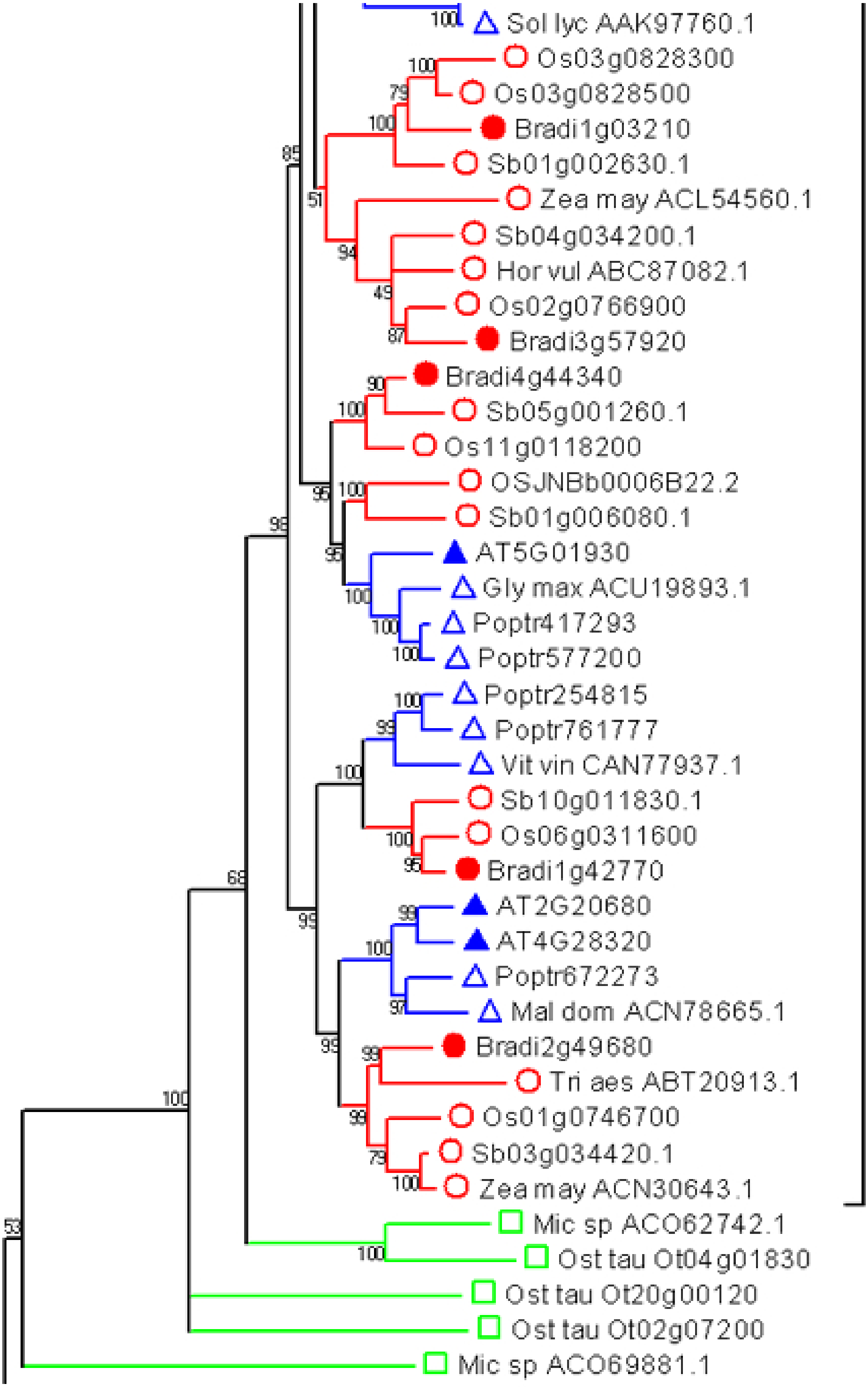
Sol lyc AAK97760.1
Os03g0828300
Os03g0828500
Bradi1g03210
Sb01g002630.1
Zea may ACL54560.1
Sb04g034200.1
Hor vul ABC87082.1
Os02g0766900
Bradi3g57920
Bradi4g44340
Sb05g001260.1
Os11g0118200
OSJNBb0006B22.2
Sb01g006080.1
AT5G01930
Gly max ACU19893.1
Poptr417293
Poptr577200
Poptr254815
Poptr761777
Vit vin CAN77937.1
Sb10g011830.1
Os06g0311600
Bradi1g42770
AT2G20680
AT4G28320
Poptr672273
Mal dom ACN78665.1
Bradi2g49680
Tri aes ABT20913.1
Os01g0746700
Sb03g034420.1
Zea may ACN30643.1
Mic sp ACO62742.1
Ost tau Ot04g01830
Ost tau Ot20g00120
Ost tau Ot02g07200
Mic sp ACO69881.1

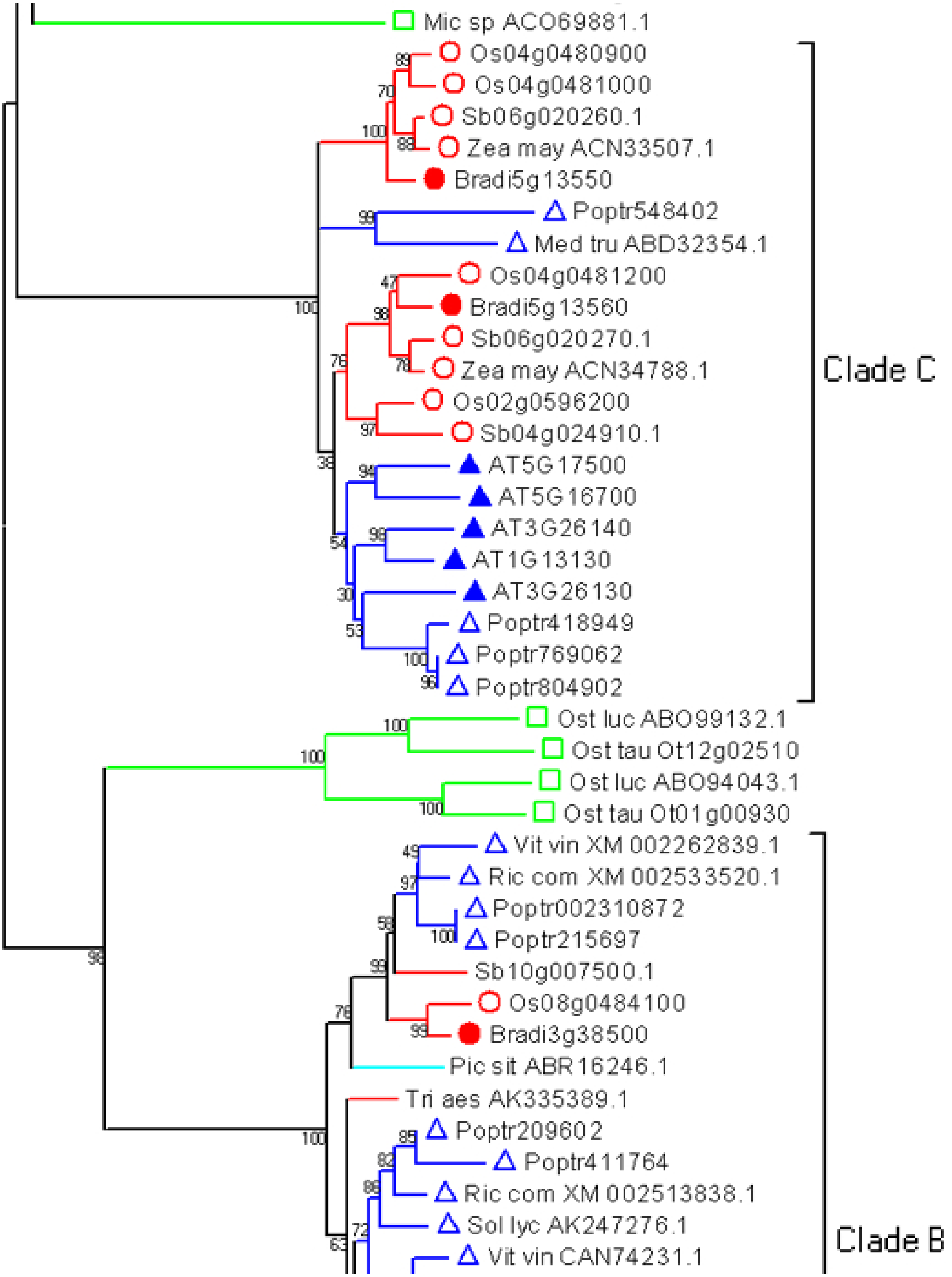
Mic sp ACO69881.1
Os04g0480900
Os04g0481000
Sb06g020260.1
Zea may ACN33507.1
Bradi5g13550
Poptr548402
Med tru ABD32354.1
Os04g0481200
Bradi5g13560
Sb06g020270.1
Zea may ACN34788.1
Os02g0596200
Sb04g024910.1
AT5G17500
AT5G16700
AT3G26140
AT1G13130
AT3G26130
Poptr418949
Poptr769062
Poptr804902
Clade C
Ost luc ABO99132.1
Ost tau Ot12g02510
Ost luc ABO94043.1
Ost tau Ot01g00930
Vit vin XM 002262839.1
Ric com XM 002533520.1
Poptr002310872
Poptr215697
Sb10g007500.1
Os08g0484100
Bradi3g38500
Pic sit ABR16246.1
Tri aes AK335389.1
Poptr209602
Poptr411764
Ric com XM 002513838.1
Sol lyc AK247276.1
Vit vin CAN74231.1
Clade B

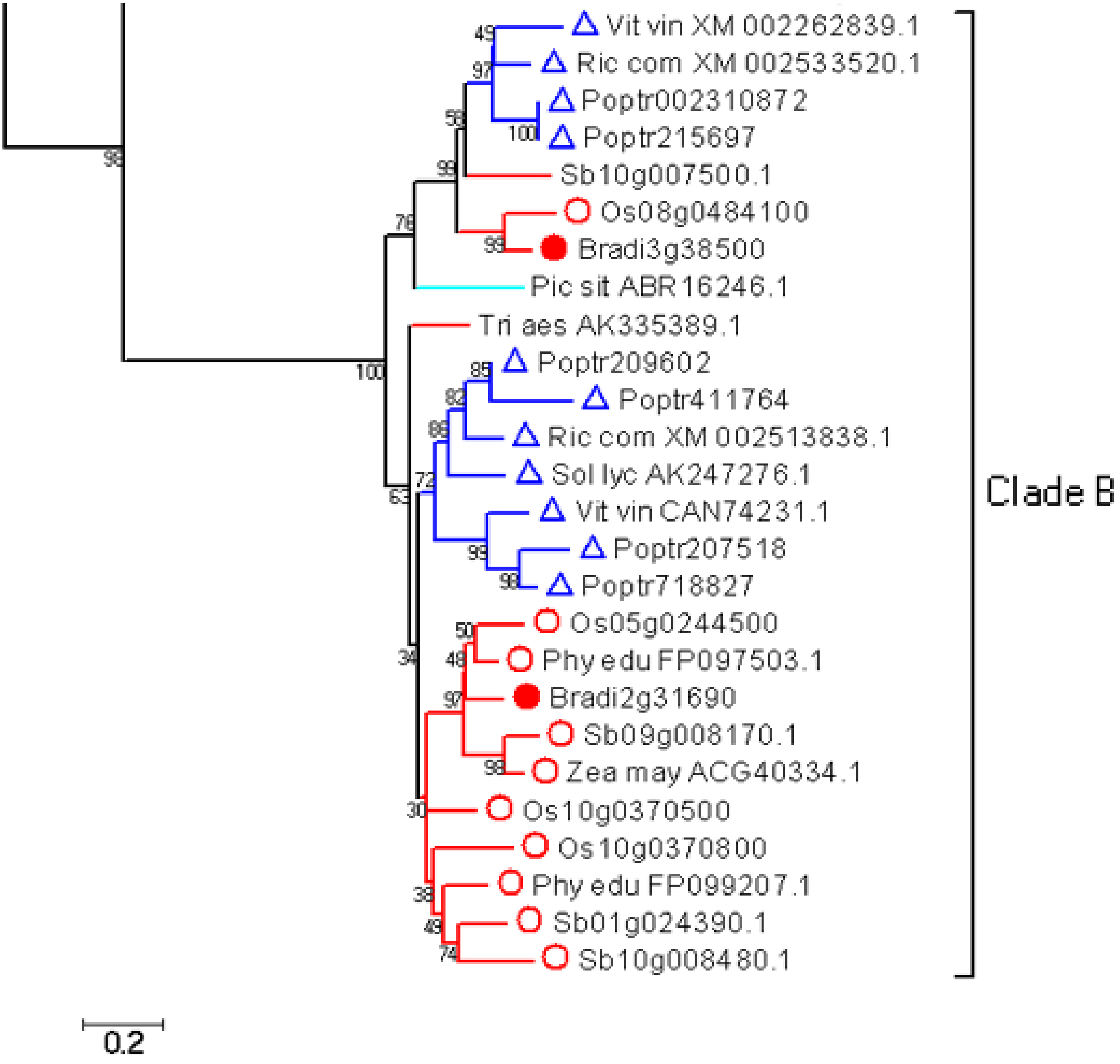
Vit vin XM 002262839.1
Ric com XM 002533520.1
Poptr002310872
Poptr215697
Sb10g007500.1
Os08g0484100
Bradi3g38500
Pic sit ABR16246.1
Tri aes AK335389.1
Poptr209602
Poptr411764
Ric com XM 002513838.1
Sol lyc AK247276.1
Vit vin CAN74231.1
Poptr207518
Poptr718827
Os05g0244500
Phy edu FP097503.1
Bradi2g31690
Sb09g008170.1
Zea may ACG40334.1
Os10g0370500
Os10g0370800
Phy edu FP099207.1
Sb01g024390.1
Sb10g008480.1
Clade B
0.2

Additional file 13 - GH5 Rectangular Tree. GH5 family tree with all branches labeled. The same tree as in Figure 5 is displayed in a rectangular format, with complete branch information. The tree is comprised of GH5 proteins from 27 species, including *Arabidopsis* (AT), rice (Os), *Brachypodium* (Bradi), sorghum (Sb), and poplar (Poptr). The GH5 encoded by Os12g0117300 is likely fragmentary and was omitted from the analysis. Additional plant and green algal sequences are indicated by a six-letter abbreviation for the genus and species, followed by the GenBank accession number or gene name. The tree was constructed using the Neighbor-Joining method and 1,000 bootstrap replicates. The bootstrap support for each branch is indicated. Distances represent the number of amino-acid substitutions per site. Sequences from eudicots are indicated in blue (*Arabidopsis* with filled triangles, other eudicots with open triangles); sequences from monocots are indicated in red (*Brachypodium* with filled circles, other monocots with open circles). A light blue diamond indicates the single gymnosperm sequence; green squares indicate green algal sequences. Brackets indicate the three major plant clades, A, B, and C. Barley HvMAN1 corresponds to Hor vul ABC87082.1, LeMAN4a to Sol lyc AAK97760.1, and rice GH5BG to Os10g0370500. Act arg: *Actinidia arguta*; Cof ara: *Coffea arabica*; Dau car: *Daucus carota*; Gly max: *Glycine max*; Hor vul: *Hordeum vulgare*; Lac sat: *Lactuca sativ*a; Mal dom: *Malus x domestica*; Med tru: *Medicago truncatula*; Mic sp: *Micromonas* sp. RCC299; Mus acu: *Musa acuminata*; Ost luc: *Ostreococcus lucimarinus*; Ost tau: *Ostreococcus tauri*; Phy edu: *Phyllostachys edulis*; Pic sit: *Picea sitchensis*; Pru per: *Prunus persica*; Pru sal: *Prunus salicina*; Ric com: *Ricinus communis*; Sol lyc: *Solanum lycopersicum*; The hal: *Thellungiella halophila*; Tri aes: *Triticum aestivum*; Vac cor: *Vaccinium corymbosum*; Vit vin: *Vitis vinifera*; Zea may: *Zea mays*. Common names for these species can be found in additional file 11. Poplar gene names are abbreviated; for the full names see additional file 9.
