# Supplementary material for: Annotation and comparative analysis of the glycoside hydrolase genes in Brachypodium distachyon
Source: BMC Genomics. 2010 Oct 25;11:600. doi: 10.1186/1471-2164-11-600 (PMC3091745; doi:10.1186/1471-2164-11-600)
Supplement: Additional file 14 — GH28 Rectangular Tree. GH28 Rectangular Tree This figure presents the same phylogenetic tree as Figure 6, but in a rectangular format, with complete bootstrap information and branch labels. The tree includes GH28 proteins from Arabidopsis, poplar, rice, Brachypodium, sorghum, and maize. [file 1471-2164-11-600-S14.PDF]

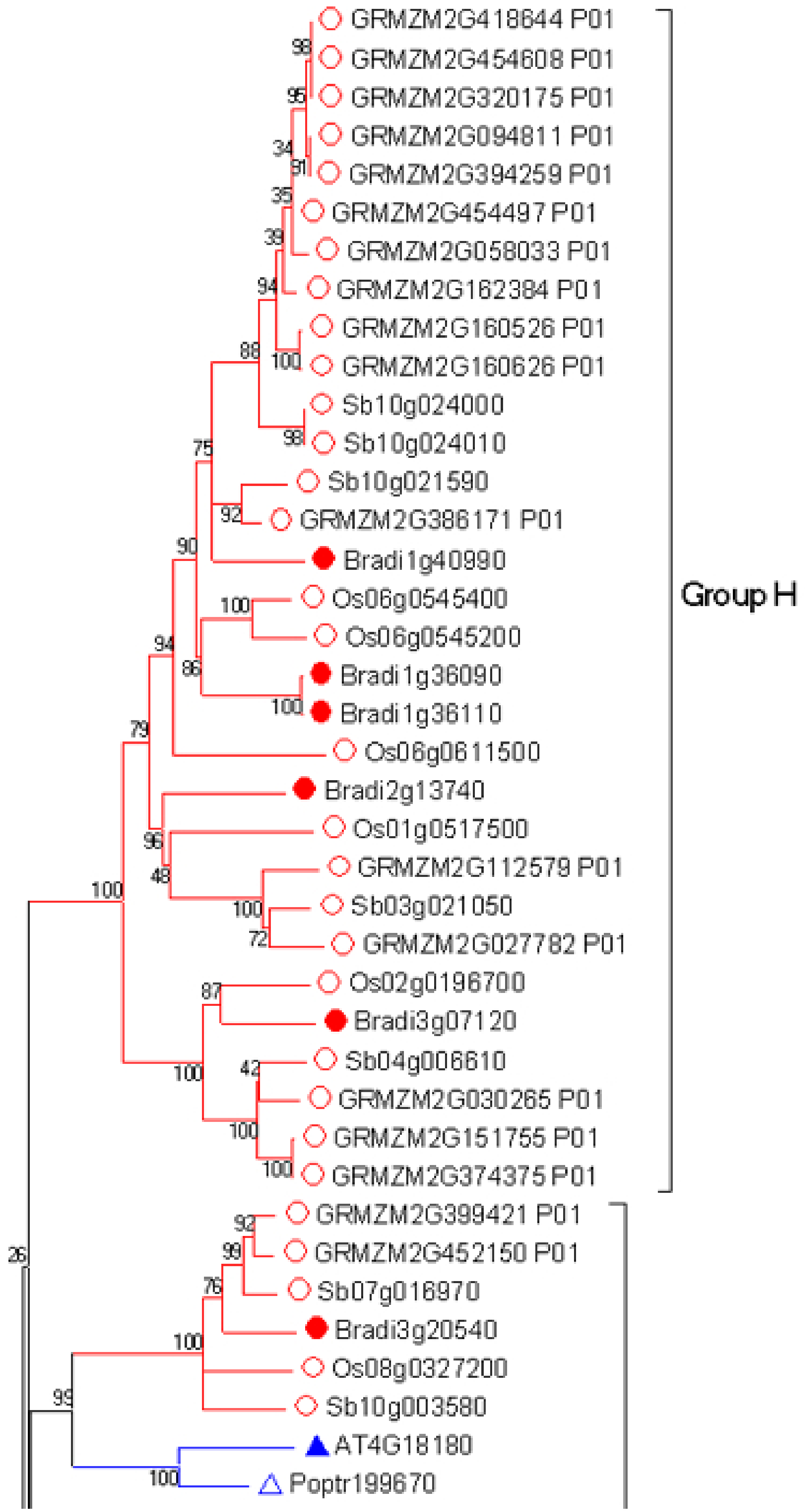

GRMZM2G418644 P01
GRMZM2G454608 P01
GRMZM2G320175 P01
GRMZM2G094811 P01
GRMZM2G394259 P01
GRMZM2G454497 P01
GRMZM2G058033 P01
GRMZM2G162384 P01
GRMZM2G160526 P01
GRMZM2G160626 P01
Sb10g024000
Sb10g024010
Sb10g021590
GRMZM2G386171 P01
Bradi1g40990
Os06g0545400
Os06g0545200
Bradi1g36090
Bradi1g36110
Os06g0611500
Bradi2g13740
Os01g0517500
GRMZM2G112579 P01
Sb03g021050
GRMZM2G027782 P01
Os02g0196700
Bradi3g07120
Sb04g006610
GRMZM2G030265 P01
GRMZM2G151755 P01
GRMZM2G374375 P01
GRMZM2G399421 P01
GRMZM2G452150 P01
Sb07g016970
Bradi3g20540
Os08g0327200
Sb10g003580
AT4G18180
Poptr199670
Group H

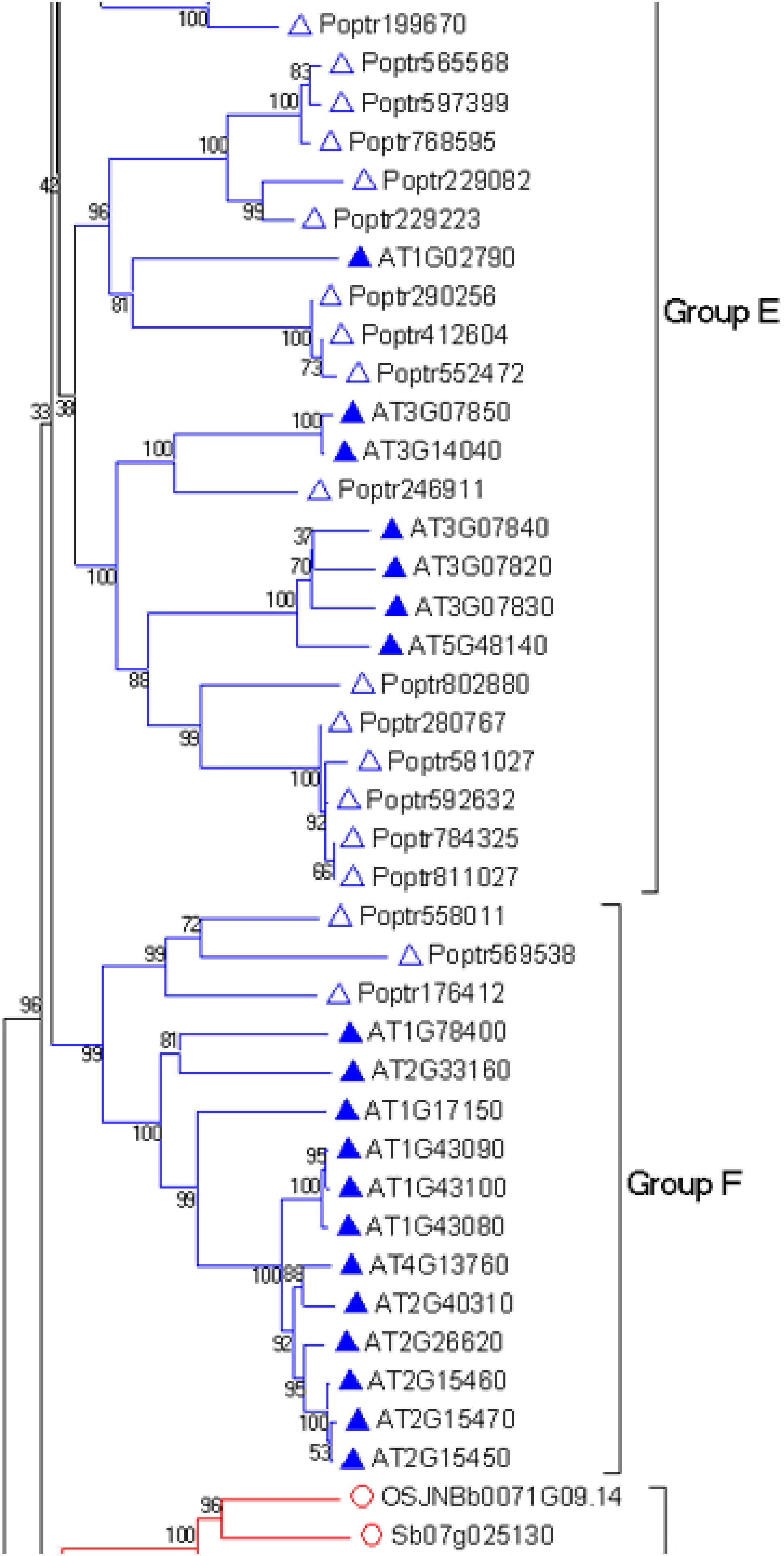
Poptr199670
Poptr585568
Poptr597399
Poptr768595
Poptr229082
Poptr229223
AT1G02790
Poptr290256
Poptr412604
Poptr552472
AT3G07850
AT3G14040
Poptr246911
AT3G07840
AT3G07820
AT3G07830
AT5G48140
Poptr802880
Poptr280767
Poptr581027
Poptr592632
Poptr784325
Poptr811027
Group E
Poptr558011
Poptr569538
Poptr176412
AT1G78400
AT2G33160
AT1G17150
AT1G43090
AT1G43100
AT1G43080
AT4G13760
AT2G40310
AT2G26620
AT2G15460
AT2G15470
AT2G15450
Group F
OSJNBb0071G09.14
Sb07g025130

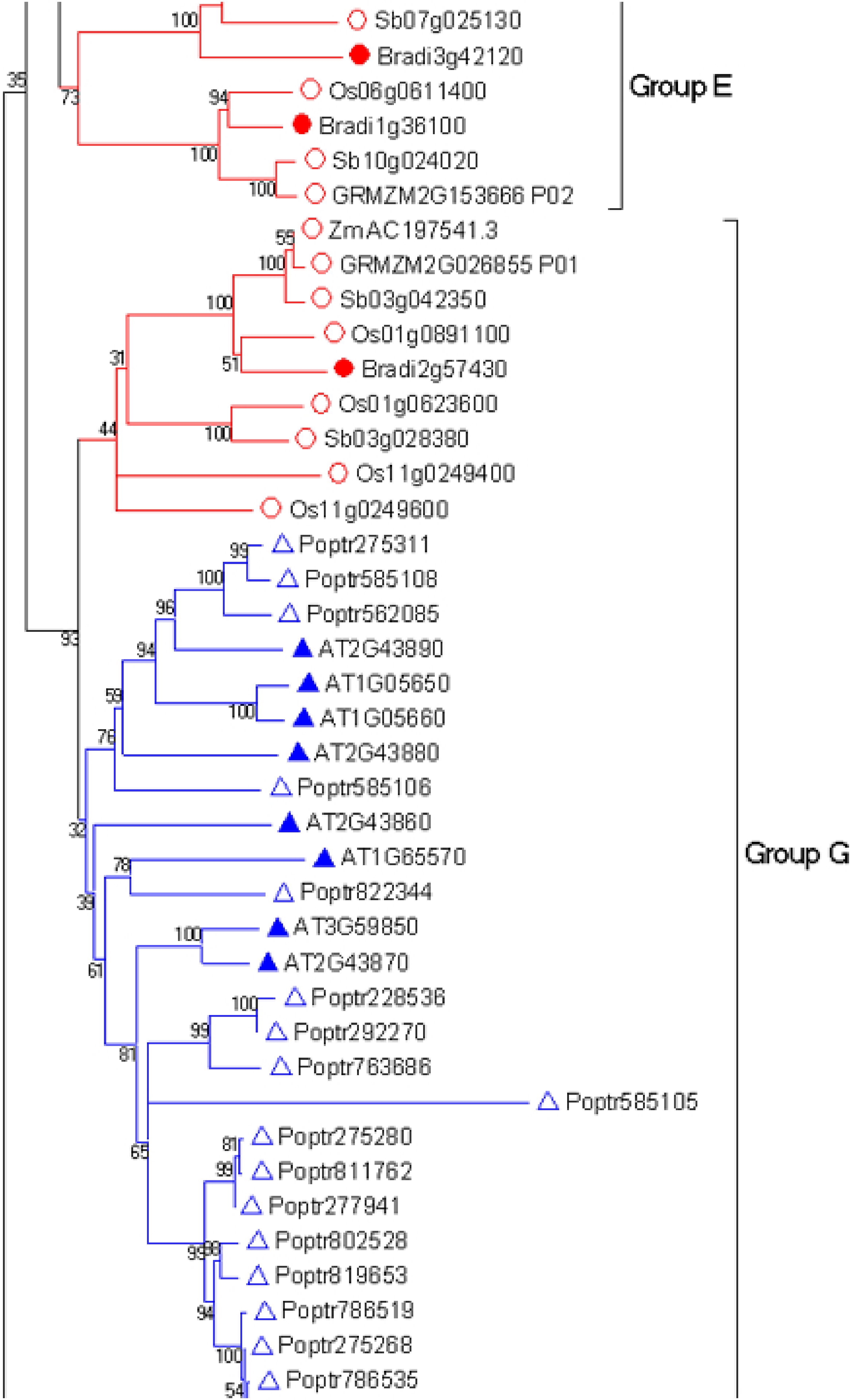

Sb07g025130
Bradi3g42120
Group E
Os06g0611400
Bradi1g36100
Sb10g024020
GRMZM2G153666 P02
ZmAC197541.3
GRMZM2G026855 P01
Sb03g042350
Os01g0891100
Bradi2g57430
Os01g0623600
Sb03g028380
Os11g0249400
Os11g0249600
Poptr275311
Poptr585108
Poptr562085
AT2G43890
AT1G05650
AT1G05660
AT2G43880
Poptr585106
AT2G43860
AT1G65570
Group G
Poptr822344
AT3G59850
AT2G43870
Poptr228536
Poptr292270
Poptr763686
Poptr585105
Poptr275280
Poptr811762
Poptr277941
Poptr802528
Poptr819653
Poptr786519
Poptr275268
Poptr786535

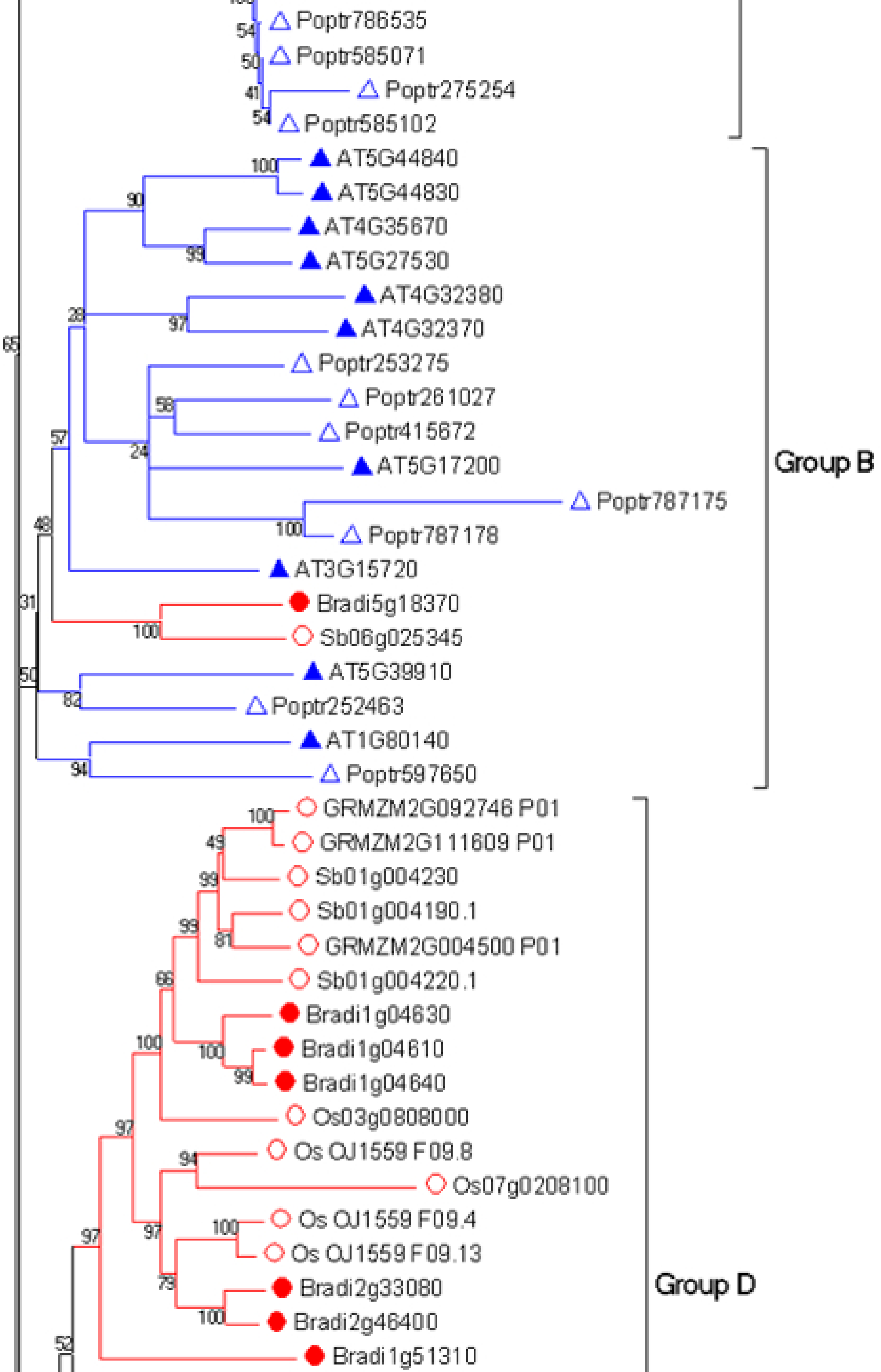

Poptr786535
Poptr585071
Poptr275254
Poptr585102
AT5G44840
AT5G44830
AT4G35670
AT5G27530
AT4G32380
AT4G32370
Poptr253275
Poptr261027
Poptr415672
AT5G17200
Poptr787175
Poptr787178
AT3G15720
Bradi5g18370
Sb06g025345
AT5G39910
Poptr252463
AT1G80140
Poptr597650
Group B
GRMZM2G092746 P01
GRMZM2G111609 P01
Sb01g004230
Sb01g004190.1
GRMZM2G004500 P01
Sb01g004220.1
Bradi1g04630
Bradi1g04610
Bradi1g04640
Os03g0808000
Os OJ1559 F09.8
Os07g0208100
Os OJ1559 F09.4
Os OJ1559 F09.13
Bradi2g33080
Bradi2g46400
Bradi1g51310
Group D

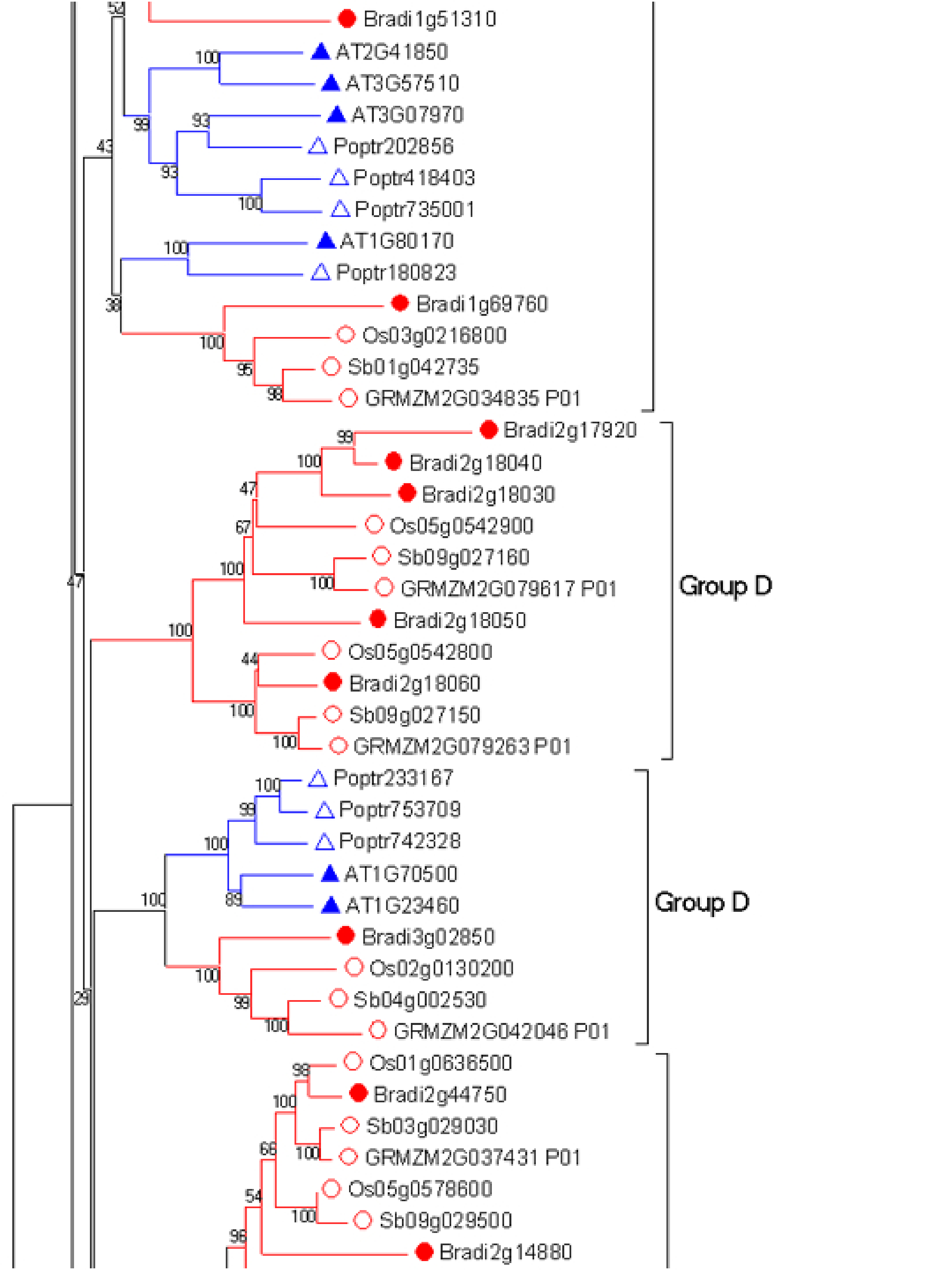
Bradi1g51310
AT2G41850
AT3G57510
AT3G07970
Poptr202856
Poptr418403
Poptr735001
AT1G80170
Poptr180823
Bradi1g69760
Os03g0216800
Sb01g042735
GRMZM2G034835 P01
Bradi2g17920
Bradi2g18040
Bradi2g18030
Os05g0542900
Sb09g027160
GRMZM2G079617 P01
Group D
Bradi2g18050
Os05g0542800
Bradi2g18060
Sb09g027150
GRMZM2G079263 P01
Poptr233167
Poptr753709
Poptr742328
AT1G70500
AT1G23460
Group D
Bradi3g02850
Os02g0130200
Sb04g002530
GRMZM2G042046 P01
Os01g0636500
Bradi2g44750
Sb03g029030
GRMZM2G037431 P01
Os05g0578600
Sb09g029500
Bradi2g14880

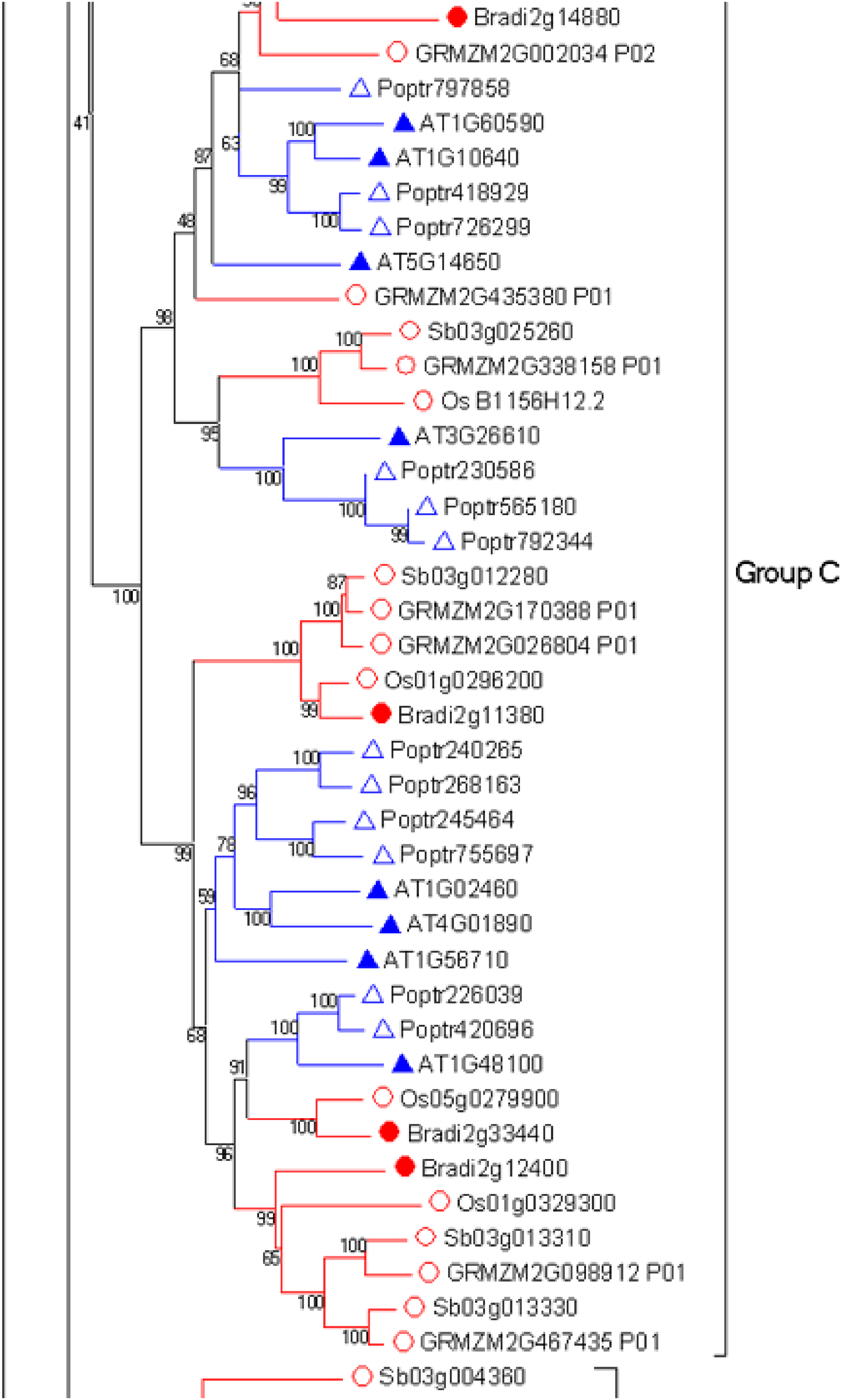
Bradi2g14880
GRMZM2G002034 P02
Poptr797858
AT1G60590
AT1G10640
Poptr418929
Poptr726299
AT5G14650
GRMZM2G435380 P01
Sb03g025260
GRMZM2G338158 P01
Os B1156H12.2
AT3G26610
Poptr230586
Poptr565180
Poptr792344
Sb03g012280
GRMZM2G170388 P01
GRMZM2G026804 P01
Os01g0296200
Bradi2g11380
Poptr240265
Poptr268163
Poptr245464
Poptr755697
AT1G02460
AT4G01890
AT1G56710
Poptr226039
Poptr420696
AT1G48100
Os05g0279900
Bradi2g33440
Bradi2g12400
Os01g0329300
Sb03g013310
GRMZM2G098912 P01
Sb03g013330
GRMZM2G467435 P01
Sb03g004360
Group C

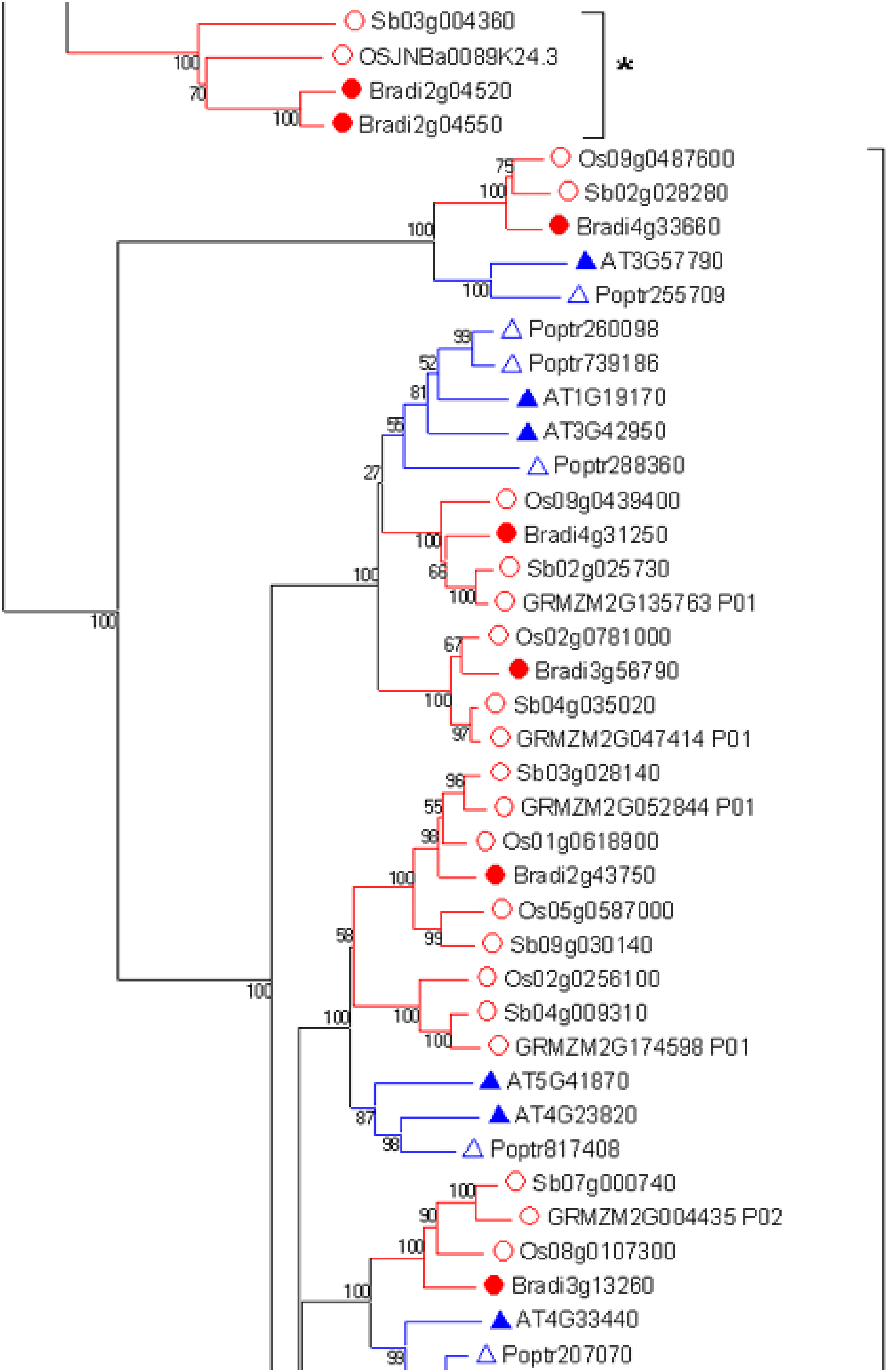

Sb03g004360
OSJNBa0089K24.3
Bradi2g04520
Bradi2g04550
*
Os09g0487600
Sb02g028280
Bradi4g33660
AT3G57790
Poptr255709
Poptr260098
Poptr739186
AT1G19170
AT3G42950
Poptr288360
Os09g0439400
Bradi4g31250
Sb02g025730
GRMZM2G135763 P01
Os02g0781000
Bradi3g56790
Sb04g035020
GRMZM2G047414 P01
Sb03g028140
GRMZM2G052844 P01
Os01g0618900
Bradi2g43750
Os05g0587000
Sb09g030140
Os02g0256100
Sb04g009310
GRMZM2G174598 P01
AT5G41870
AT4G23820
Poptr817408
Sb07g000740
GRMZM2G004435 P02
Os08g0107300
Bradi3g13260
AT4G33440
Poptr207070

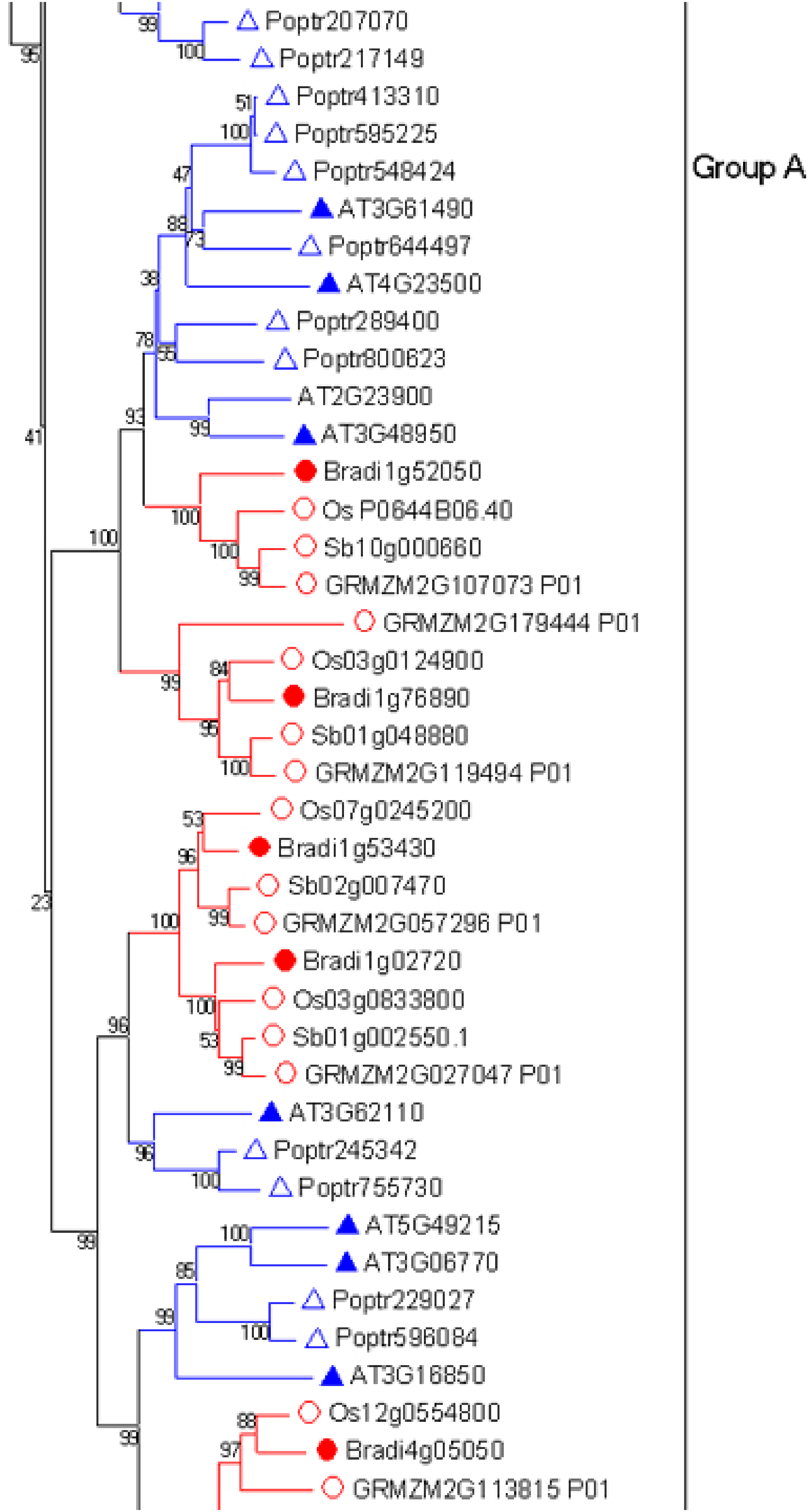
Poptr207070
Poptr217149
Poptr413310
Poptr595225
Poptr548424
AT3G61490
Poptr644497
AT4G23500
Poptr289400
Poptr800623
AT2G23900
AT3G48950
Bradi1g52050
Os P0644B06.40
Sb10g000660
GRMZM2G107073 P01
GRMZM2G179444 P01
Os03g0124900
Bradi1g76890
Sb01g048880
GRMZM2G119494 P01
Os07g0245200
Bradi1g53430
Sb02g007470
GRMZM2G057296 P01
Bradi1g02720
Os03g0833800
Sb01g002550.1
GRMZM2G027047 P01
AT3G62110
Poptr245342
Poptr755730
AT5G49215
AT3G06770
Poptr229027
Poptr596084
AT3G16850
Os12g0554800
Bradi4g05050
GRMZM2G113815 P01
Group A

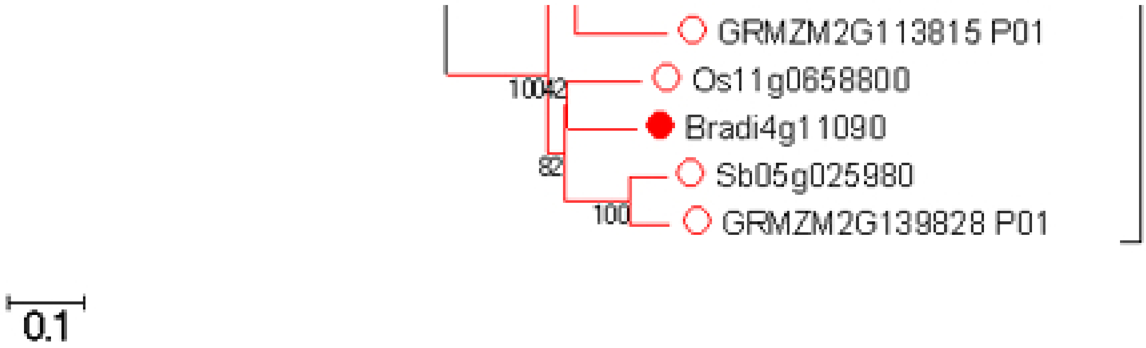
GRMZM2G113815 P01
Os11g0658800
Bradi4g11090
Sb05g025980
GRMZM2G139828 P01
100
42
82
100
0.1

Additional file 14 - GH28 Rectangular Tree. GH28 family tree with all branches labeled. The same tree as in Figure 6 is displayed in a rectangular format, with complete branch information. The tree includes GH28 proteins from *Arabidopsis* (AT), rice (Os), *Brachypodium* (Bradi), sorghum (Sb), poplar (Poptr), and maize (Zm or GRMZM). The GH28 proteins encoded by Poptr274990, Poptr587953, and Poptr640527 are relatively short and were omitted from the analysis. The maize proteins encoded by AC210013.4 and AC231180.2 were reported to be GH28 family members [90] but were omitted from the analysis, because they did not contain Pfam-predicted GH domains. The tree was constructed using the Neighbor-Joining method and 1,000 bootstrap replicates. The bootstrap support for each branch is indicated. Distances represent the number of amino-acid substitutions per site. Sequences from eudicots are indicated in blue (*Arabidopsis* with filled triangles, poplar with open triangles); sequences from grasses are indicated in red (*Brachypodium* with filled circles; rice, sorghum, and maize with open circles). Brackets mark groups according to the designations of Penning *et al* [90]. QRT2 corresponds to At3g07970, ADPG1 to At3g57510, and ADPG2 to At2g41850. Poplar gene names are abbreviated; for the full names, see additional file 9.
