# Supplementary material for: Annotation and comparative analysis of the glycoside hydrolase genes in Brachypodium distachyon
Source: BMC Genomics. 2010 Oct 25;11:600. doi: 10.1186/1471-2164-11-600 (PMC3091745; doi:10.1186/1471-2164-11-600)
Supplement: Additional file 15 — GH51 Rectangular Tree. GH51 Rectangular Tree This figure presents the same phylogenetic tree as Figure 7, but in a rectangular format, with complete bootstrap information. The tree includes GH51 proteins from Arabidopsis, poplar, rice, Brachypodium, sorghum, and 14 other plants. [file 1471-2164-11-600-S15.PDF]

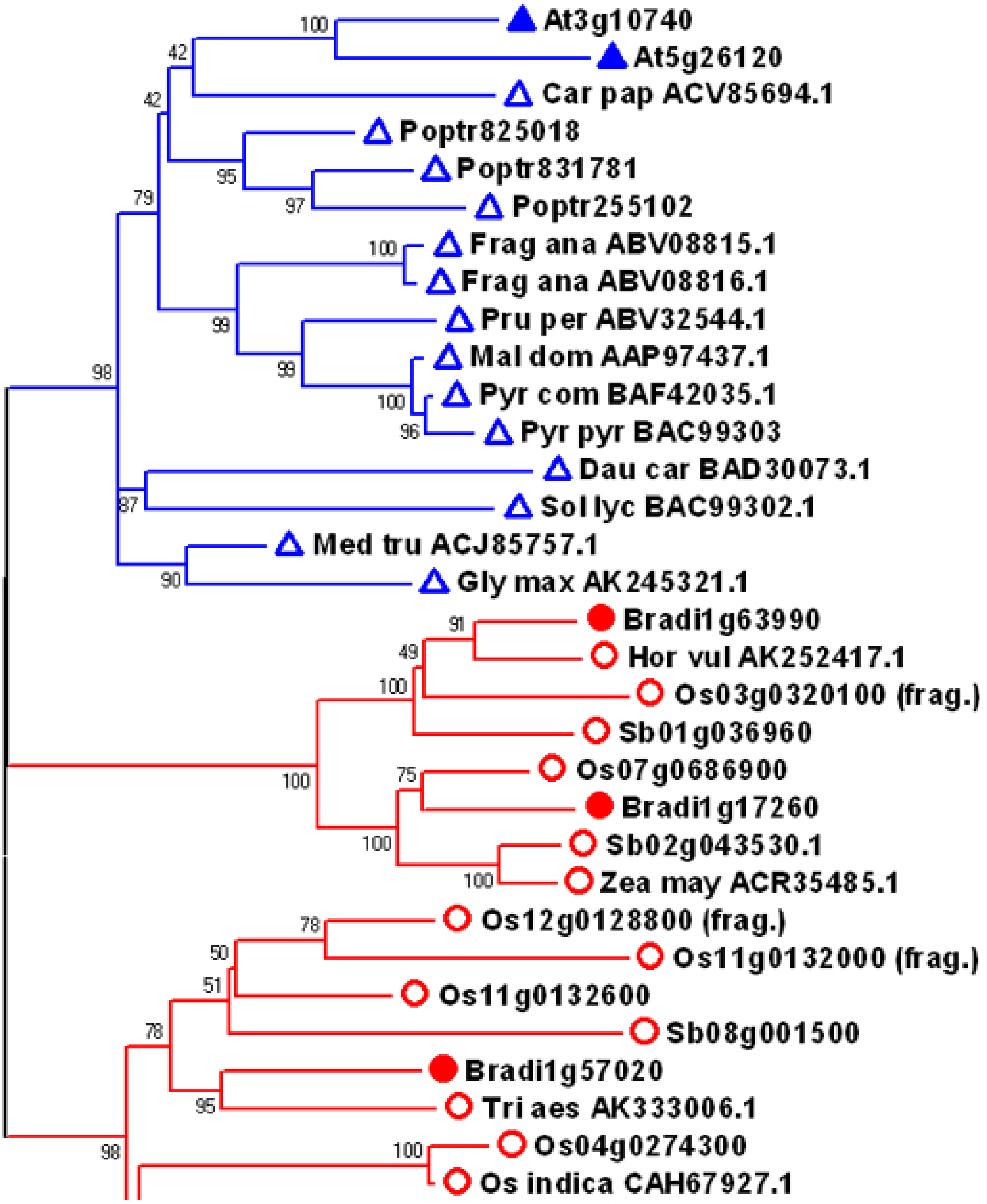

At3g10740
At5g26120
Car pap ACV85694.1
Poptr825018
Poptr831781
Poptr255102
Frag ana ABV08815.1
Frag ana ABV08816.1
Pru per ABV32544.1
Mal dom AAP97437.1
Pyr com BAF42035.1
Pyr pyr BAC99303
Dau car BAD30073.1
Sol lyc BAC99302.1
Med tru ACJ85757.1
Gly max AK245321.1
Bradi1g63990
Hor vul AK252417.1
Os03g0320100 (frag.)
Sb01g036960
Os07g0686900
Bradi1g17260
Sb02g043530.1
Zea may ACR35485.1
Os12g0128800 (frag.)
Os11g0132000 (frag.)
Os11g0132600
Sb08g001500
Bradi1g57020
Tri aes AK333006.1
Os04g0274300
Os indica CAH67927.1
100
42
42
95
97
79
100
99
99
100
96
98
87
90
91
49
100
100
75
100
100
78
50
51
78
95
98
100

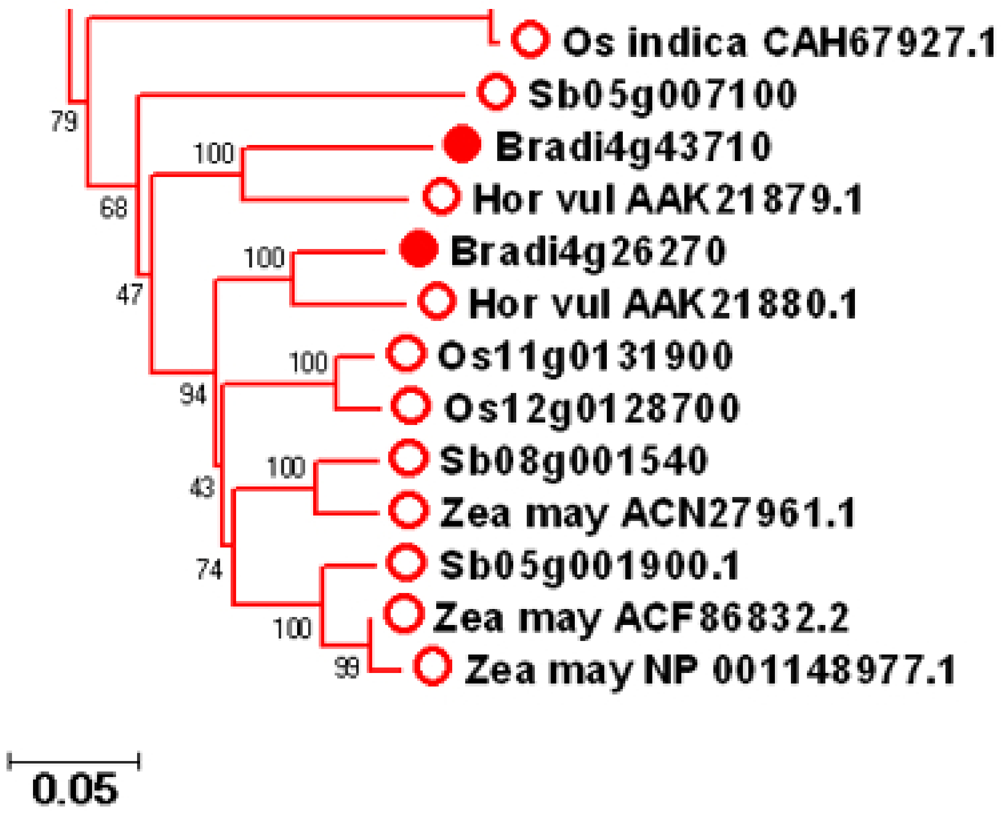
Os indica CAH67927.1
Sb05g007100
Bradi4g43710
Hor vul AAK21879.1
Bradi4g26270
Hor vul AAK21880.1
Os11g0131900
Os12g0128700
Sb08g001540
Zea may ACN27961.1
Sb05g001900.1
Zea may ACF86832.2
Zea may NP 001148977.1
79
68
100
47
100
100
94
100
43
74
100
99
0.05

Additional file 15 - GH51 Rectangular Tree. The GH51 family tree with additional bootstrap values. The same tree as in Figure 7 is displayed in a rectangular format, with additional bootstrap values. The tree includes GH51 proteins from 18 species: *Arabidopsis* (AT), rice (Os), *Brachypodium* (Bradi), sorghum (Sb), and poplar (Poptr), as well as sequences from other eudicots and grasses (labeled with a genus-plus-species abbreviation and the GenBank accession number). The tree was constructed using the Neighbor-Joining method and 1,000 bootstrap replicates. The bootstrap support for each branch is indicated. Distances represent the number of amino-acid substitutions per site. Barley AXAH-I corresponds to Hor vul AAK21879.1, *Arabidopsis* ARAF1 to At3g10740, strawberry Ara1 and 2 to Frag ana ABV08815.1 and ABV08816.1, and peach ARF1 to Pru per ABV32544.1. Sequences from eudicots are indicated in blue (*Arabidopsis* with filled triangles, other eudicots with open triangles); sequences from grasses are indicated in red (*Brachypodium* with filled circles, other grasses with open circles). Car pap: *Carica papaya*; Dau car: *Daucus carota*; Fra ana: *Fragaria x ananassa*; Gly max: *Glycine max*; Hor vul: *Hordeum vulgare*; Mal dom: *Malus x domestica*; Med tru: *Medicago truncatula*; Os indica: *Oryza sativa* Indica Group; Pru per: *Prunus persica*; Pyr com: *Pyrus communis*; Pyr pyr: *Pyrus pyrifolia*; Sol lyc: *Solanum lycopersicum*; Tri aes: *Triticum aestivum*; Zea may: *Zea mays*. Common names for these species can be found in additional file 11. Poplar gene names are abbreviated; for the full names, see additional file 9.
