# Supplementary material for: Annotation and comparative analysis of the glycoside hydrolase genes in Brachypodium distachyon
Source: BMC Genomics. 2010 Oct 25;11:600. doi: 10.1186/1471-2164-11-600 (PMC3091745; doi:10.1186/1471-2164-11-600)
Supplement: Additional file 16 — GH13 Rectangular Tree. GH13 Rectangular Tree This figure presents the same phylogenetic tree as Figure 8, but in a rectangular format, with complete bootstrap information. The tree includes GH13 proteins from Arabidopsis, poplar, rice, Brachypodium, and sorghum. [file 1471-2164-11-600-S16.PDF]

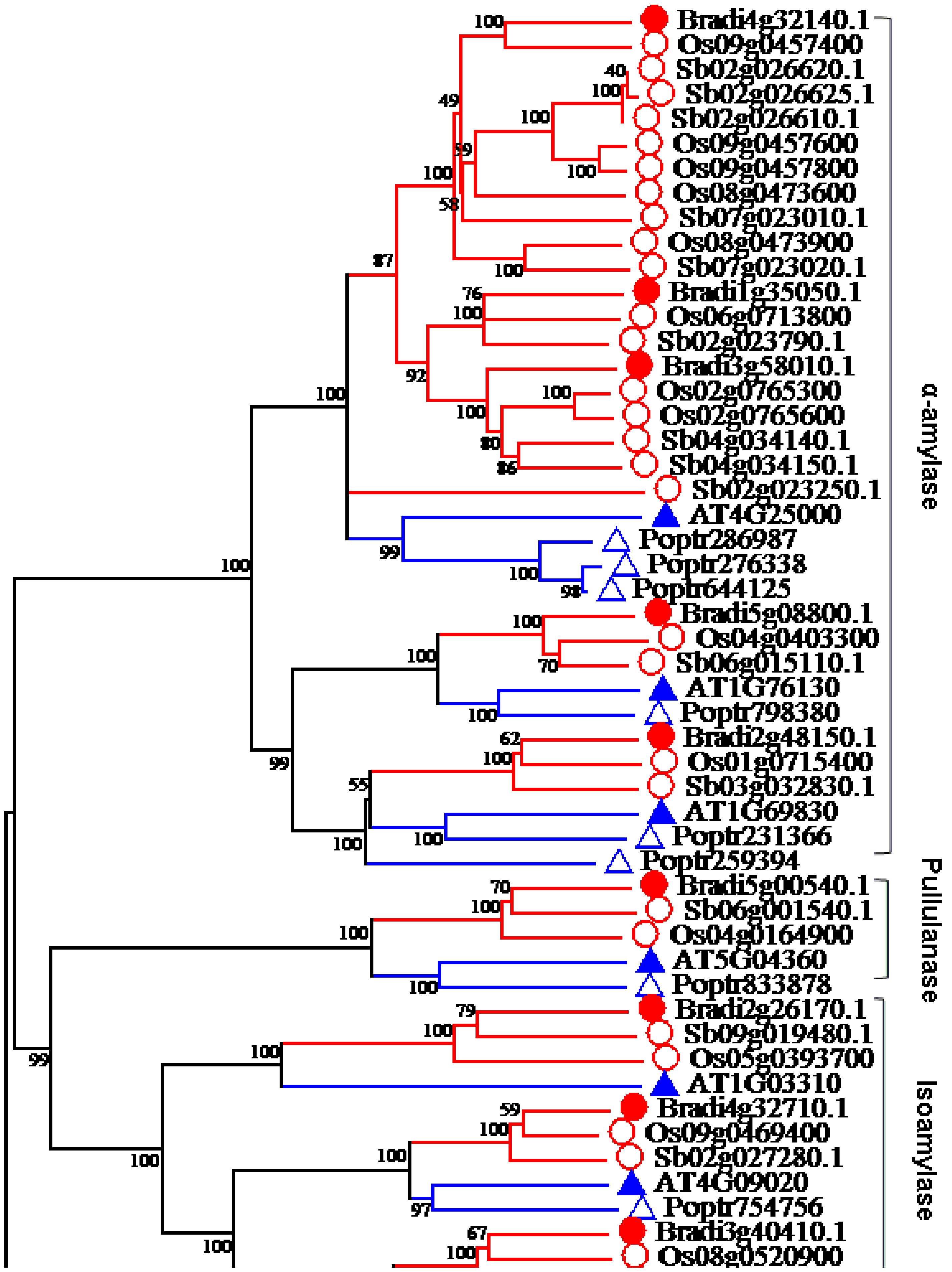
Bradi4g32140.1
Os09g0457400
Sb02g026620.1
Sb02g026625.1
Sb02g026610.1
Os09g0457600
Os09g0457800
Os08g0473600
Sb07g023010.1
Os08g0473900
Sb07g023020.1
Bradi1g35050.1
Os06g0713800
Sb02g023790.1
Bradi3g58010.1
Os02g0765300
Os02g0765600
Sb04g034140.1
Sb04g034150.1
Sb02g023250.1
AT4G25000
Poptr286987
Poptr276338
Poptr644125
Bradi5g08800.1
Os04g0403300
Sb06g015110.1
AT1G76130
Poptr798380
Bradi2g48150.1
Os01g0715400
Sb03g032830.1
AT1G69830
Poptr231366
Poptr259394
Bradi5g00540.1
Sb06g001540.1
Os04g0164900
AT5G04360
Poptr833878
Bradi2g26170.1
Sb09g019480.1
Os05g0393700
AT1G03310
Bradi4g32710.1
Os09g0469400
Sb02g027280.1
AT4G09020
Poptr754756
Bradi3g40410.1
Os08g0520900
α-amylase
Pullulanase
Isoamylase

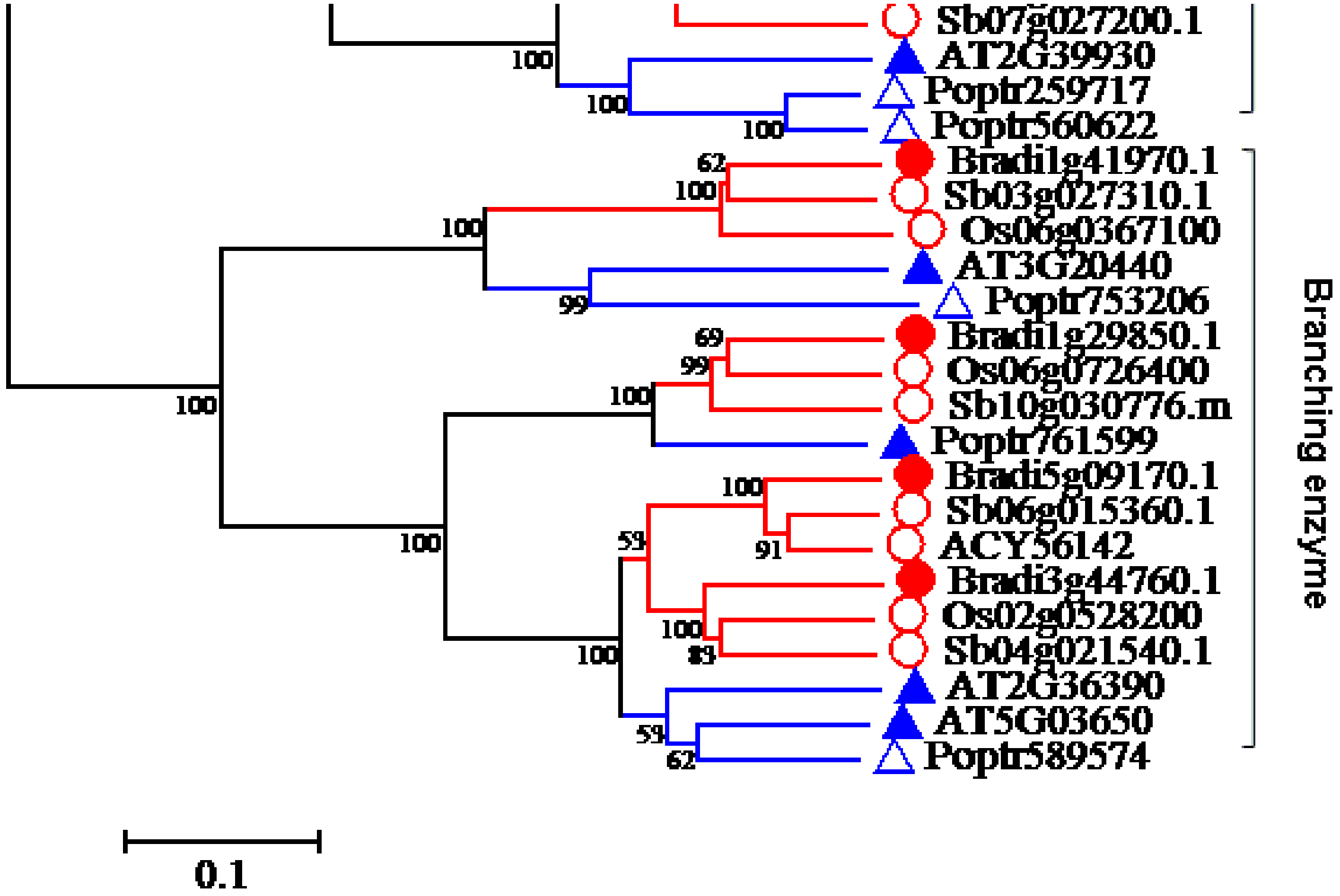

Additional file 16 – GH13 Rectangular Tree. The GH13 family tree with additional bootstrap values. The same tree as in Figure 8 is displayed in a rectangular format. The tree includes proteins from *Arabidopsis* (AT), rice, *Brachypodium* (Bradi), sorghum, and poplar. The tree was constructed using the Neighbor-Joining method and 1,000 bootstrap replicates. The percent of bootstrap replicates supporting each branch is indicated. Distances are proportional to the number of amino-acid substitutions per site. Sequences from eudicots are indicated in blue (*Arabidopsis* with filled triangles, poplar with open triangles); sequences from grasses are indicated in red (*Brachypodium* with filled circles, rice and sorghum with open circles). The three clades, α-amylases, branching enzymes, and debranching enzymes including pullulanases and isoamylases, are marked with brackets.
